# Radiative transfer with reciprocal transactions: Numerical method and its implementation

**DOI:** 10.1371/journal.pone.0210155

**Published:** 2019-01-08

**Authors:** Timo Väisänen, Johannes Markkanen, Antti Penttilä, Karri Muinonen

**Affiliations:** 1 Department of Physics, University of Helsinki, Helsinki, Finland; 2 Max Planck Institute for Solar System Research, Göttingen, Germany; 3 Finnish Geospatial Research Institute FGI, National Land Survey of Finland, Helsinki, Finland; Voevodsky Institute of Chemical Kinetics and Combustion, RUSSIAN FEDERATION

## Abstract

We present a numerical method for solving electromagnetic scattering by dense discrete random media entitled radiative transfer with reciprocal transactions (R^2^T^2^). The R^2^T^2^ is a combination of the Monte Carlo radiative-transfer, coherent-backscattering, and superposition *T*-matrix methods. The applicability of the radiative transfer is extended to dense random media by incorporating incoherent volume elements containing multiple particles. We analyze the R^2^T^2^ by comparing the results with the asymptotically exact superposition *T*-matrix method, and show that the R^2^T^2^ removes the caveats of radiative-transfer methods by comparing it to the RT-CB. We study various implementation choices that result in an accurate and efficient numerical algorithm. In particular, we focus on the properties of the incoherent volume elements and their effects on the final solution.

## 1 Introduction

Computing light scattering by a large dense multi-particle system with exact numerical methods such as the superposition *T*-matrix method (STMM) [1–3] or the finite-difference time-domain method [4] becomes impossible due to the enormous computational time consumption. Therefore, approximate methods for light scattering by dense multi-particle systems need to be developed.

Radiative transfer (RT) (e.g., see [5]) is an approximate method which works for large sparse systems and is widely used, e.g., in the studies of atmospheric sciences [6] and cosmic dust clouds [7]. Still, the RT has relatively good applicability for small sparse media [8]. The radiative transfer equation (RTE) describes how electromagnetic radiation traverses through random media. The RTE can be derived from the Maxwell equations by incorporating multiple approximations such as the independent single-particle scattering and far-field approximations among others [9]. These approximations oversimplify the system making it inapplicable to dense random media [10].

In the Monte Carlo RT (MC-RT), the RTE is solved by integrating the leading light-scattering paths known as the ladder diagrams. In the radiative-transfer coherent-backscattering method (RT-CB), the maximally crossed paths are also included in order to consider the coherent-backscattering effects [11]. These paths are traced inside the system in which scattering events are modelled as single scattering by particles modeled with the Mie theory [12] or with the Rayleigh approximation [13]. The problem with the single-particle scattering is that when the modeled system is dense, independent scattering is not a valid assumption anymore. This is due to the increased complexity of the near zone affected by the close-by scatterers. The RT algorithms have been extended to dense random media by applying the Percus-Yevick approximation (e.g., [14]), and numerically computing the extinction coefficient [15]. In [16, 17], the extinction path lengths were shown to follow the Percus-Yevick approximation experimentally. Our first study to expand RT with the incoherent first-order scattering has also been promising [18].

The scattered electromagnetic field can be mathematically divided into an ensemble-averaged field (coherent or mean field) and to a deviating part (incoherent field). The incoherent field can be used to produce an accurate prediction for the diffuse extinction in dense random media [15]. We develop a method based on this notion called the radiative transfer with reciprocal transactions (R^2^T^2^, see S1 Source Code) [19]. The R^2^T^2^ is an MC-RT method which has been developed to solve multiple-scattering problems of dense random media, and the method was shown to work with irregular particles [20].

In the R^2^T^2^, the applicability of the RT is extended to dense random media by using incoherent volume elements consisting of clusters of particles instead of single scatterers. The rationalization for the incoherent field treatment is that the RTE can be written for the diffuse (incoherent) and coherent specific intensity column vectors [9]. Due to this separation, the RTE should be solved separately for incoherent and coherent parts. The current implementation of the R^2^T^2^ considers the incoherent part. The incoherent volume elements have been used as a base for other simpler methods presented in [18] and [21]. The latter study entails an application to Comet 67P/Churyumov-Gerasimenko. Still, these incoherent volume-element methods lack published studies of the effects of, e.g., the size of the volume element to the results.

The RTE is missing the capability of producing an intensity peak in the forward-scattering direction because the peak derives from diffraction. The intensity peak and the negative polarization observed near the backscattering direction are a product of an effect called coherent backscattering, which is not present in the RTE [9]. Fortunately, the CB can be added to the RT solution separately as in the RT-CB [11] and a similar algorithm is implemented into the R^2^T^2^.

In this paper, the R^2^T^2^ method is presented in detail with effects of the volume element size on the results. The R^2^T^2^ supports any kind of incoherent volume elements, but in this paper, only spherical scatterers are utilized. Some theoretical concepts are described in Sect 2, which is followed by an assessment of the numerical methods in Sect 3. The implementation choices and effects of the volume element size are studied in more detail, as well as the required precomputations. In Sect 4, the R^2^T^2^ is validated by comparing it to the semi-analytical Fast Superposition *T*-matrix Method (FaSTMM) [22] and compared to the radiative-tranfer method RT-CB. The results are discussed in Sect 5 and the conclusions are presented in Sect 6.

## 2 Basic concepts

### 2.1 *T*-matrix

An arbitrary electric field can be represented with the coefficients *a*_*wvu*_ (*u* = 1, 2) as

$$\begin{array}{c} E^{\left(\nu \right)}=\sum_{v=1}^{N_{o}}\sum_{w=-v}^{v}\left(a_{vw1}M_{vw}^{\left(\nu \right)}+a_{vw2}N_{vw}^{\left(\nu \right)}\right). \end{array}$$ (1)

Here $M_{vw}^{\left(\nu \right)}$ and $N_{vw}^{\left(\nu \right)}$ are base vectors composed of vector spherical wave functions and *N*_*o*_ is the degree of expansion needed to assure convergence. The superscript *ν* describes either the incoming or outgoing field expansion based on the spherical Bessel or Hankel functions, respectively. By studying boundary problems, it is possible to find a relation, the so-called *T*-matrix [23], between the incoming (*f*_*vwu*_) and outgoing (*a*_*vwu*_) electric field coefficients,

$$\begin{array}{c} (\begin{array}{c} a_{wv1}\\ a_{wv2} \end{array})=T(\begin{array}{c} f_{wv1}\\ f_{wv2} \end{array}). \end{array}$$ (2)

This *T*-matrix models the scattering properties of a single scatterer or a group of them, and can be used to model any particle or particle group provided that the *T*-matrix can be solved.

### 2.2 Scattering matrix

The electric field components *E*_*θ*_ and *E*_*ϕ*_ derived from Eq 1 can be used to define a vector of 4 elements called the Stokes parameters [24] (from now on Stokes vector),

$$\begin{array}{c} I=[\begin{array}{c} I\\ Q\\ U\\ V \end{array}]=[\begin{array}{c} E_{\theta }E_{\theta }^{*}+E_{\phi }E_{\phi }^{*}\\ E_{\theta }E_{\theta }^{*}-E_{\phi }E_{\phi }^{*}\\ -E_{\theta }E_{\phi }^{*}-E_{\phi }E_{\phi }^{*}\\ i(E_{\phi }E_{\theta }^{*}-E_{\theta }E_{\phi }) \end{array}]. \end{array}$$ (3)

The R^2^T^2^ outputs scattering matrices **S** as a function of scattering angle *θ* and the azimuthal angle *ϕ*. The scattering matrix maps the incident Stokes vector **I**_inc_ to the scattered Stokes vector **I**_sca_ in the same way as in Eq 2,

$$\begin{array}{c} I_{\text{sca}}=\frac{1}{k^{2}r^{2}}SI_{\text{inc}}, \end{array}$$ (4)

in which *r* is the distance to the observation point and *k* is the wave number. With the help of the wavelength λ, the wave number is $k=\frac{2\pi }{\lambda }$. The explanation of the full scattering matrix and the analysis of each element is available in the literature, e.g. [24].

### 2.3 Incoherent scattering properties

The scattered electric field **E**^sca^ can be decomposed into the incoherent field **E**^s,ic^ and the ensemble-averaged field (mean or coherent field) **E**^sca,c^,

$$\begin{array}{c} E^{\text{sca}}=E^{\text{sca},\text{ic}}+E^{\text{sca},c}. \end{array}$$ (5)

The coherent field for a random system is computed by solving the scattered fields multiple times *L* for different configurations and by averaging them:

$$\begin{array}{c} E^{\text{sca},c}=\lim_{L\to \infty }\frac{1}{L}\sum_{i=1}^{L}E_{i}^{\text{sca}}. \end{array}$$ (6)

It is also possible to find the coherent field coefficients in terms of the *T*-matrix, i.e., averaging over multiple *T*-matrices, which results in the coherent *T*^c^-matrix:

$$\begin{array}{c} T^{c}=\lim_{L\to \infty }\frac{1}{L}\sum_{i=1}^{L}T_{i}. \end{array}$$ (7)

Thus, the incoherent *T*-matrix for the realization *i* reads

$$\begin{array}{c} T_{i}^{\text{ic}}=T_{i}-T^{c}. \end{array}$$ (8)

The scattering cross sections are computed for the incoherent ($C_{\text{sca}}^{\text{ic}}$) and the free-space fields (*C*_sca_) using the corresponding coefficients ($a_{wvu}^{\text{ic}}$, *a*_*wvu*_) from

$$\begin{array}{c} \begin{array}{c} C_{\text{sca}}^{\text{ic}}=\frac{1}{k^{2}}\sum_{v=1}^{N_{o}}\sum_{w=-v}^{v}\left(\right|a_{wv1}^{\text{ic}}{|}^{2}+|a_{wv2}^{\text{ic}}{|}^{2}),\\ C_{\text{sca}}=\frac{1}{k^{2}}\sum_{v=1}^{N_{o}}\sum_{w=-v}^{v}\left(\right|a_{wv1}{|}^{2}+|a_{wv2}{|}^{2}). \end{array} \end{array}$$ (9)

The absorption cross section (*C*_abs_) is defined by

$$\begin{array}{c} C_{\text{abs}}=\sum_{k=1}^{N_{V}}C_{\text{abs},k}, \end{array}$$ (10)

in which *C*_abs,*k*_ is the total absorption of the single scatterer and *N*_*V*_ is the number of volume elements. These cross-sections can be used to define the incoherent extinction mean free path length *ℓ* (from now on, briefly mean free path) with the scalar extinction coefficient $\kappa_{\text{ext}}^{\text{ic}}$,

$$\begin{array}{c} \begin{array}{c} \kappa_{\text{ext}}^{\text{ic}}=\frac{C_{\text{ext}}^{\text{ic}}}{V}=\frac{C_{\text{sca}}^{\text{ic}}+C_{\text{abs}}}{V},\\ \ell =\frac{1}{\kappa_{\text{ext}}^{\text{ic}}},\;\;\;\; \end{array} \end{array}$$ (11)

in which *V* is the volume of the volume element.

The incoherent albedo $\tilde{\omega }$ can be defined for a volume element *i* as

$$\begin{array}{c} {\tilde{\omega }}_{i}=\frac{C_{\text{sca},i}^{\text{ic}}}{C_{\text{sca},i}^{\text{ic}}+C_{\text{abs},i}}, \end{array}$$ (12)

with the corresponding incoherent scattering cross section $C_{\text{sca},i}^{\text{ic}}$.

## 3 Numerical methods

### 3.1 Algorithm for radiative transfer with reciprocal transactions

The R^2^T^2^ is based on the MC-RT, in which the RTE is solved by integrating the light-scattering paths associated with the ladder diagrams inside the medium. *N* rays are traced inside the medium with different initial polarization states. In order to generate the entire Mueller matrix, six polarization states, linear (vertical, horizontal, +45°, -45°) and circular (right- and left-handed) [24], are traced, meaning that in a system without symmetry, 6*N* light-scattering paths need to be generated. The number of the required polarization states can be reduced if there are symmetries in the system or if the user is not interested in some of the Mueller matrix elements. For example, spherical media require only two initial polarization states (linear and circular). In this paper, only the phase function and degree of linear polarization of a spherical system are studied and circular polarization is omitted. This reduces the number of traced rays to *N*.

The overall algorithm is summarized in Algorithm 1, which is almost identical to the scheme present in the RT-CB [11]. For simplicity, the term “intensity” is used for “flux density”.

**Algorithm 1**: **Pseudocode for the R^2^T^2^**.

**foreach** Polarization state (*Q*, *U*, *V*) **do**

 **for** *i* = 0 **to** *N* **do**

  Set intensity *I* = 1 (see Sect 3); Set initial polarization state (see Sect 3.1.1);

  Compute direct transmission *I*_d_ (see Sect 3.1.1);

  Set intensity *I* ⇐ *I* − *I*_d_ (see Sect 3.1.1);

  Randomize the first scattering location (see Sect 3.1.1);

  **while** *I* < *I*_cutoff_ **do**

   Move the ray to the new location;

   Compute volume-element interaction (see Sect 3.1.2);

   Compute absorption (see Sect 3.1.2);

   Compute how much intensity escapes from the medium (*I*_p_), (peel-off, see Sect 3.1.3);

   Generate new scattering location (see Sect 3.1.4);

   Compute coherent backscattering (see Sect 3.1.5);

   Set intensity *I* ⇐ *I* − *I*_p_;

  **end**

 **end**

**end**

Form output from the peel-off and coherent backscattering data;

#### 3.1.1 Direct transmission and first scattering location

**Fig 1:**
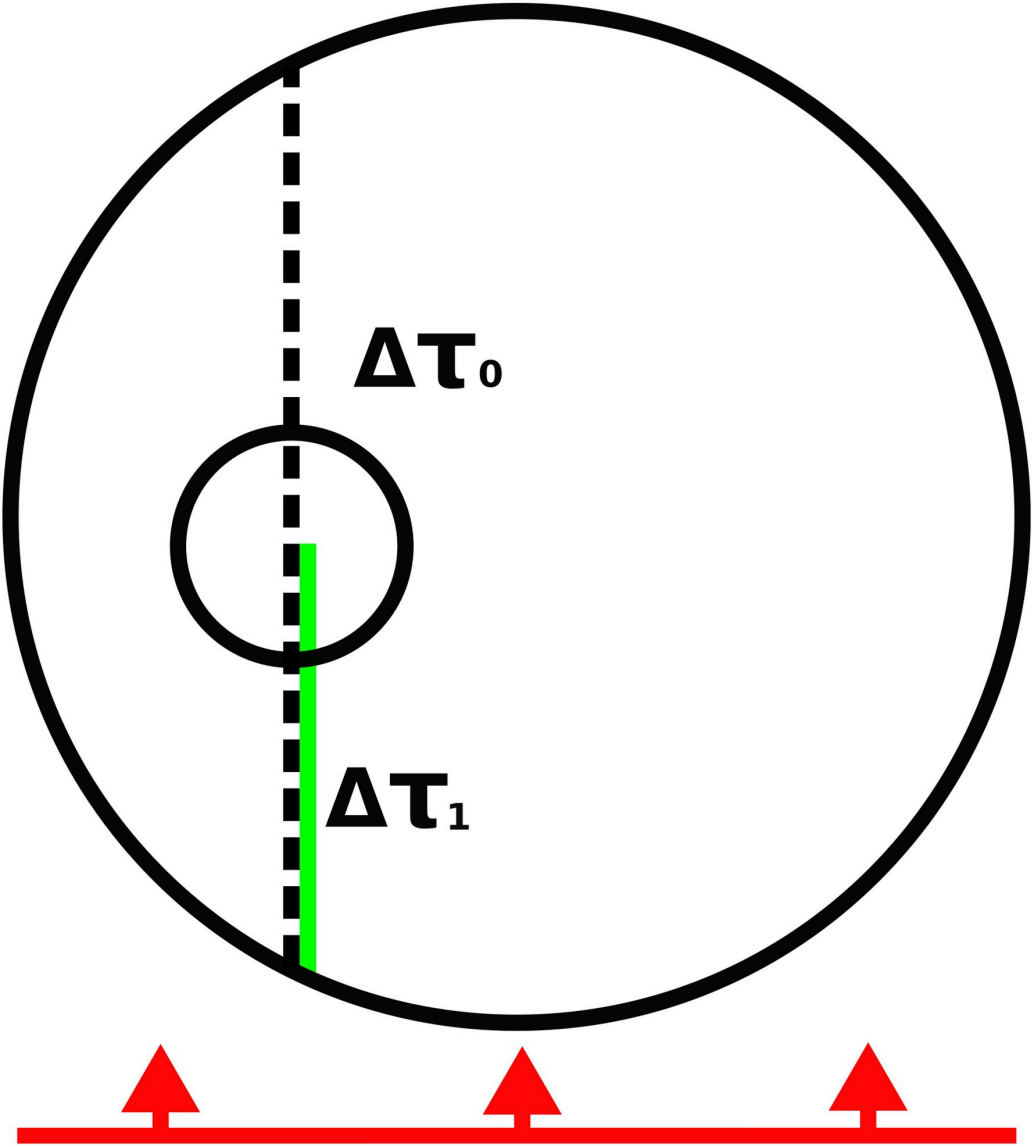
Incoming wave (red) is incident on the medium (large circle), and a random position on the surface of the medium is generated. The direct transmission is computed along the dashed line (length Δ*τ*_0_) and the initial scattering location is generated in the distance Δ*τ*_1_ from the entry point (solid green line). The volume element (small circle) is generated to the initial scattering location.

The incident field is a plane wave, and thus the initial location of the ray is generated from a uniform distribution on the plane. The ray is then given an initial polarization state and projected to the surface of the studied medium (red line with arrows, Fig 1). Let us call this an entry point. Initially, every ray has intensity *I*_0_ = 1. Some of the intensity can escape from the medium without interacting with the system (direct transmission) and this needs to be addressed separately. The intensity which is left inside the system is

$$\begin{array}{c} I_{1}=I_{0}\left(1-e^{-\Delta \tau_{0}}\right). \end{array}$$ (13)

In Eq 13, Δ*τ*_0_ is the optical depth (optical depth is dimensionless, $\tau =\frac{\Delta x}{\ell }$, where Δ*x* is the distance and *ℓ* is the incoherent mean free path) between the entry and the direct exit point acquired by letting the ray move along the initial direction. From now on, *I*_*n*_ is the intensity of the ray after *n* scattering processes.

After the entry point is selected, the interaction distance to the first scattering process inside the medium is drawn from

$$\begin{array}{c} \Delta \tau_{1}=-\ln\left(1-u\left(1-e^{-\Delta \tau_{0}}\right)\right), \end{array}$$ (14)

in which *u* is a uniform random number within [0,1[.

#### 3.1.2 Volume-element interactions

The ray is accompanied with the exact presentations of the incoherent incoming and outgoing waves presented with the coefficients $f_{wvu}^{n}$ and $a_{wvu}^{n}$ at each scattering event *n* (see Eq 1). In the first scattering event, the coefficients $f_{wvu}^{1}$ correspond to the well-known plane wave coefficients according to the initial polarization state of the ray (see Algorithm 1, step 1). The incoherent scattering coefficients are thus calculated as

$$\begin{array}{c} a_{wvu}^{1}=T_{i}^{\text{ic}}f_{wvu}^{1}, \end{array}$$ (15)

where *i* depicts a volume element 1, …, *L*. The volume element is chosen from a distribution of incoherent cross sections $C_{\text{ext},\text{i}}^{\text{ic}}$ (*i* = 1, …, *L*). In the next scattering event *n* + 1, the incident field coefficients can be computed by applying the translation addition theorem for the VSWFs, namely

$$\begin{array}{c} f_{wvu}^{n+1}=H_{n}^{n+1}a_{wvu}^{n}, \end{array}$$ (16)

in which ${\text{H}}_{n}^{n+1}$ is the matrix that maps the scattered coefficients associated to the origin of the scattering event at *n* to the incident coefficients at the origin *n* + 1. To calculate the matrix ${\text{H}}_{n}^{n+1}$, we apply the recursive point-and-shoot algorithm [25, 26].

The intensity in the MC-RT-algorithm is independent of the intensity of the exact presentation. The MC-RT can produce scattering paths of thousands of scattering processes, and due to this consecutive scattering, the coefficients can underflow if not normalized from time to time.

The absorption is accounted for by using the precomputed incoherent albedos $\tilde{\omega_{i}}$ (see Eq 12). The albedo scales the available intensity $I_{n}⇐I_{n}\tilde{\omega_{i}}$.

#### 3.1.3 Peel-off

**Fig 2:**
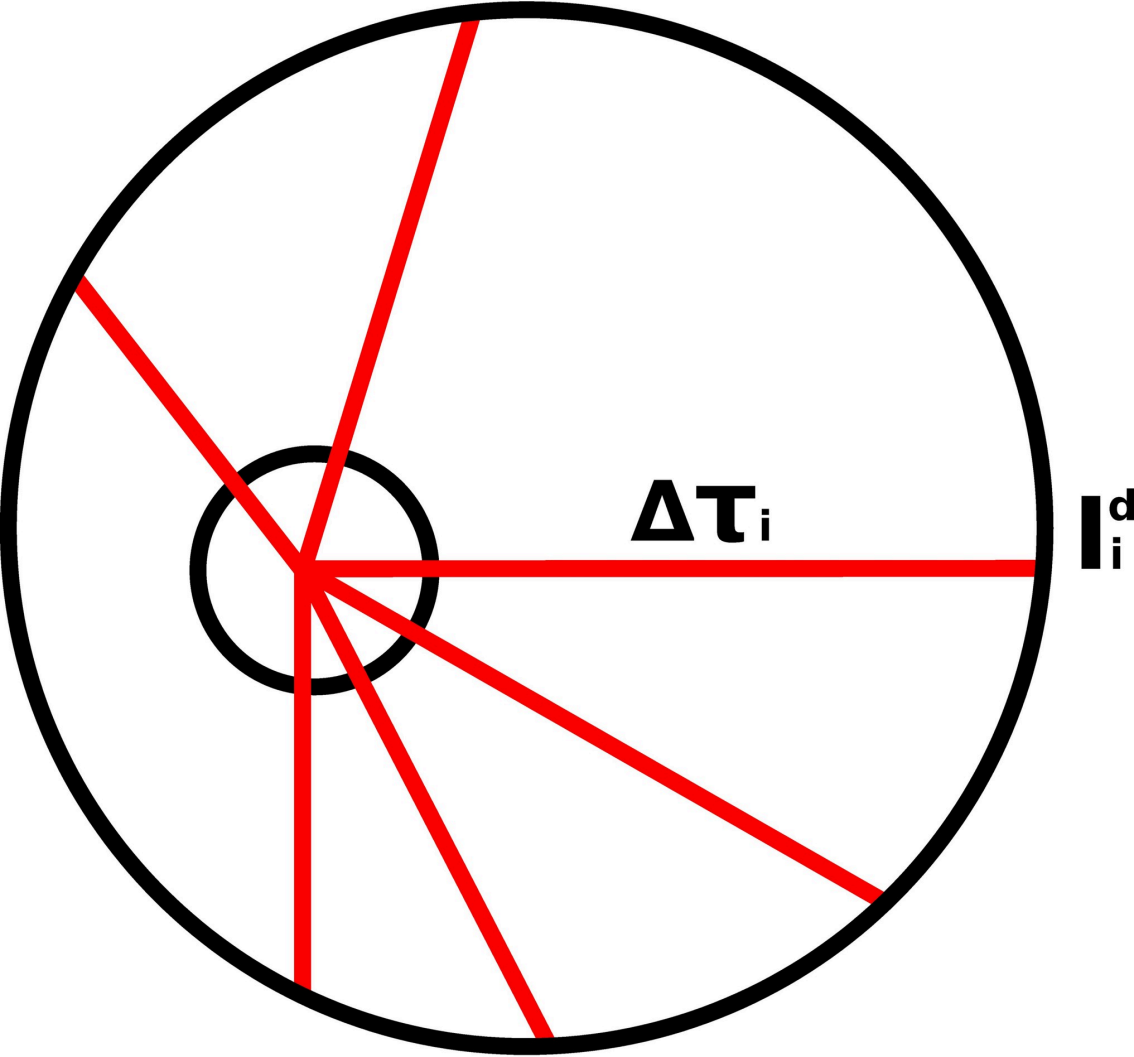
Visual presentation of peel-off. Rays (red lines) are initiated from the current volume element (small circle). They are traced toward the detectors located at infinity (escaping intensity $I_{i}^{\text{d}}$). Each red line presents these rays and the attenuation is computed along the line (Δ*τ*_*i*_).

Instead of tracing light-scattering paths and collecting individual rays which eventually escape the medium, the rays are forced to stay inside and only Stokes vectors are collected after each scattering process. This is a so-called peel-off technique developed by Yusef-Zadeh et al. [27] which accelerates the convergence of the MC-RT. After each scattering process, the intensity is scattered in the direction of the detector (bin) located at infinity (See Fig 2). By solving the attenuation Δ*τ*(*θ*, *ϕ*) along the interaction path, it is possible to compute the intensity *I*_d_ which manages to escape from the medium using

$$\begin{array}{c} I_{d}=I_{n}\sum_{i=1}^{N}\frac{I_{i}^{d}}{\sum_{j=1}^{N}I_{j}^{d}}e^{-\Delta \tau_{i}}A_{i}, \end{array}$$ (17)

in which $I_{i}^{\text{d}}$ is the incoherent intensity detected at the bin *i* without attenuation and the surface area covered by the bin is *A*_*i*_. After this, the intensity left for the next scattering is *I*_*n*+1_ = *I*_*n*_ − *I*_d_. The bins gather data cumulatively and are used to form the final output. The optical depth Δ*τ*_*i*_ can be computed from the surface or origin of the volume element and the way to do this is studied in Sect 3.2.1.

In the R^2^T^2^, there are bins along *θ*- and *ϕ*-angles covering the full solid angle. The origin of this far-field unit sphere is always at the origin of the current volume element. The full Stokes vector at the detector is solved from the electric-field coefficients obtained from Eq 3.

#### 3.1.4 Next scattering location

**Fig 3:**
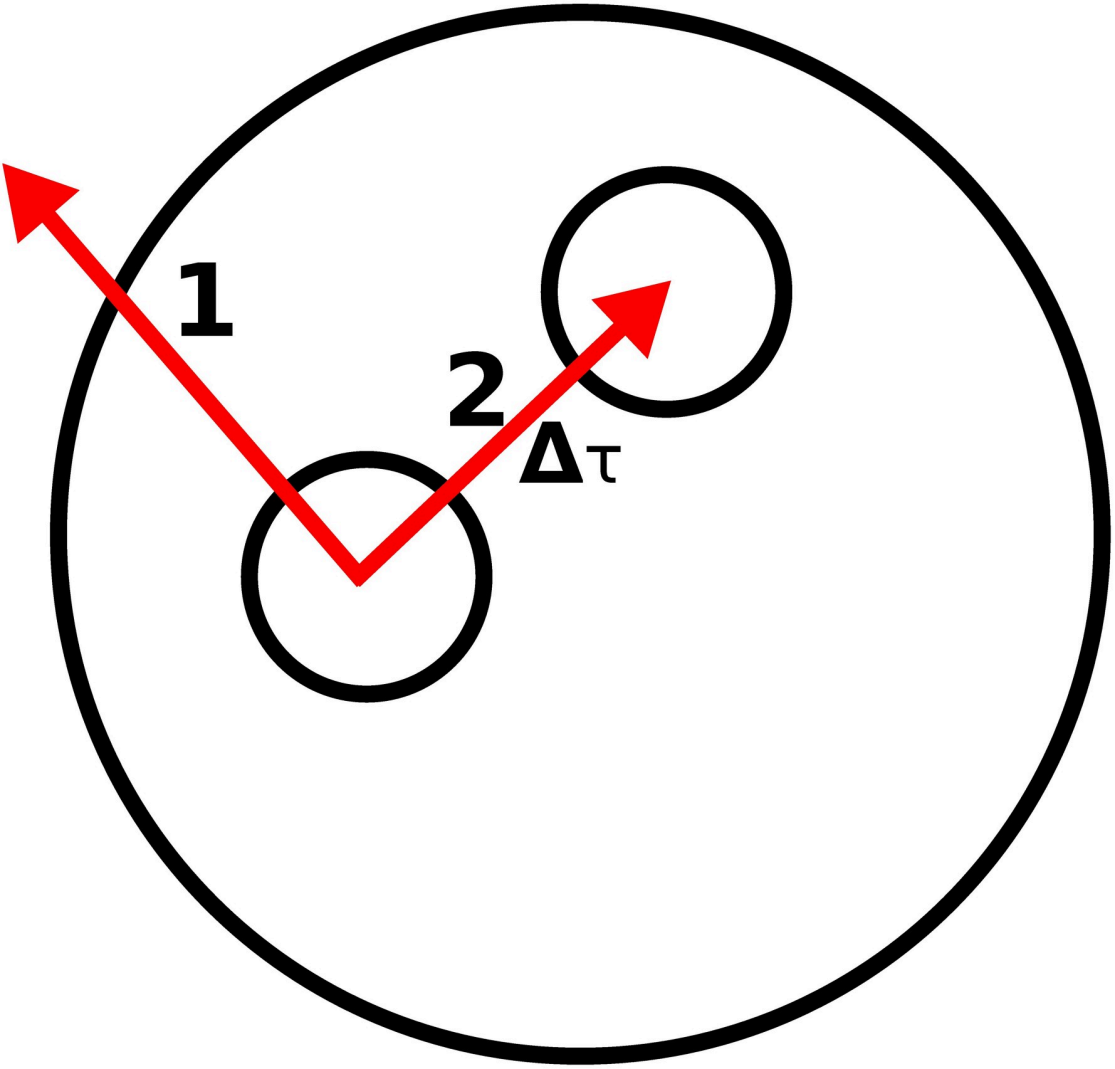
The scattering location drawn first (1) would be outside the medium, and thus a new scattering location is drawn (2). The new volume element is generated in the distance Δ*τ*. The direction distribution is the same as in the unattenuated intensity map generated for the peel-off (see Fig 2).

The new scattering direction can be drawn by using the intensity map (see Eq 17) but without attenuation $e^{-\Delta \tau_{i}}$. A cumulative distribution function *f*(*u*) is generated by summing up the intensities of bins and their weights (areas) cumulatively one by one. Now this function can be used to find the next scattering direction by generating a uniform random number *u* in [0,1[ and finding the bin *j* from

$$\begin{array}{c} \frac{1}{F}\sum_{i=0}^{j-1}I_{i}A_{i}\leq f\left(u\right)\leq \frac{1}{F}\sum_{i=0}^{j}I_{i}A_{i},\;F=\sum_{i=1}^{N}I_{i}A_{i}, \end{array}$$ (18)

in which *I*_*i*_ is the incoherent intensity in the bin *i*, *N* is the total bin count (1≤*j*≤*N*), *A*_*i*_ is the area around the bin *i*, and, to keep the algorithm consistent, *A*_0_ is set to 0. The area needs to be considered because the bins do not cover equal surface areas. To avoid using discrete scattering directions, a new random direction is drawn within the selected bin (See Fig 3).

After the scattering direction is generated, the distance to a new interaction point is estimated from

$$\begin{array}{c} \Delta \tau =-\ln(1-u), \end{array}$$ (19)

which is selected as a candidate for the next scattering location. If the location is outside the medium, a new scattering direction and distance is generated until a valid location is found inside the medium.

While the RT part of the R^2^T^2^ considers only infinitesimal points, the STMM part uses volume elements. The STMM part does not allow the volume elements to overlap which causes problems when the medium is small. For small or dense random media, the next scattering is likely to happen inside the current volume element (see Eq 17). To make near-field scattering possible, the volume elements are allowed to overlap in the RT part, but, for the STMM computation, volume elements are moved so that they do not overlap. The RT does not see the translated exact field.

In the valid location, a peel-off is executed again which is followed by the generation of a new scattering location. Tracing ends when the intensity *I*_*n*_ is less than the given cutoff intensity or the number of scattering processes reaches a given limit. A new ray is generated and the algorithm starts to trace a new light-scattering path and accumulate data in the bins. After *N* rays are traced, a new initial polarization state is selected (see Algorithm 1, step 1) and a new set of *N* rays are traced until all polarization states are completed. The peel-off data are then collected, combined, and printed for the user.

#### 3.1.5 Coherent backscattering

**Fig 4:**
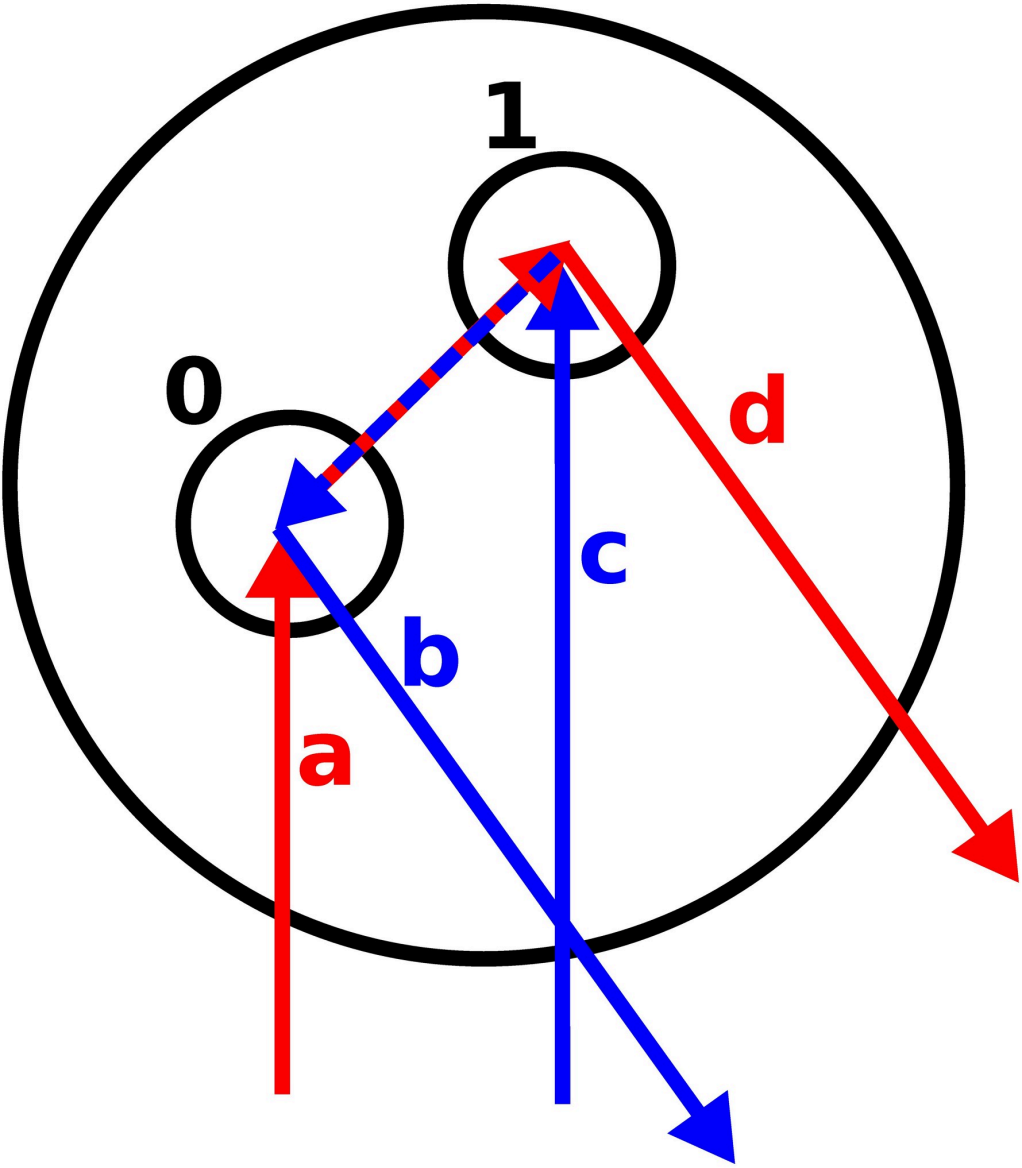
Two paths are traced for the electric fields: The original path (red, a to d) and the reversed path (blue, c to b). The direction in a and c is **k**_*i*_ and in b and d **k**_*f*_. The partial path a is pointing to **r**_0_ and partial path c to **r**_*n*_. The interference between the two paths is constructive in the backscattering direction.

The CB is added separately to the RT solution as in the RT-CB [11]. After each scattering process, the light-scattering path is traced in the reversed order. Then the reciprocity is enforced between the waves in the backscattering direction. The electric fields from both of these paths are then computed in the selected bins located in the far-field region. Phase difference needs to be accounted for by using the formula [11]

$$\begin{array}{c} \Delta \phi =k\left(k_{f}+k_{i}\right)\cdot \left(r_{n}-r_{0}\right), \end{array}$$ (20)

in which **k**_*f*_ and **k**_*i*_ are the directions of the outgoing (where the bin is located) and incident field. **r**_0_ and **r**_*n*_ are the locations of the start and the end points of the light-scattering path (see Fig 4) and after that they undergo exponential attenuation.

### 3.2 Implementation

The R^2^T^2^ is written in Fortran and parallelized using the Message Parsing Interface (MPI). Pseudo-random numbers are generated with the Fast Mersenne Twister [28] and independent streams of random numbers for each MPI process are obtained by jumping ahead [29].

Computing *T*-matrices during run-time turned out to be too compute-intensive a task before each scattering process. Instead, a set of incoherent *T*-matrices are pre-computed (see Eq 23) and sent to parallel processes in the beginning of the program. In order to prevent fixed orientations, the selected *T*-matrix is oriented using random quaternions [30] before the scattering process. The user should generate as many *T*-matrices as possible to increase variety between the scattering processes, especially, if the studied medium is large. Usually the available memory is the limiting factor for the number of the *T*-matrices.

When the *T*-matrices are computed, also albedos and scattering cross sections are stored for later use (see Sect 3.1.2).

#### 3.2.1 Peel-off distance and valid scattering location

**Fig 5:**
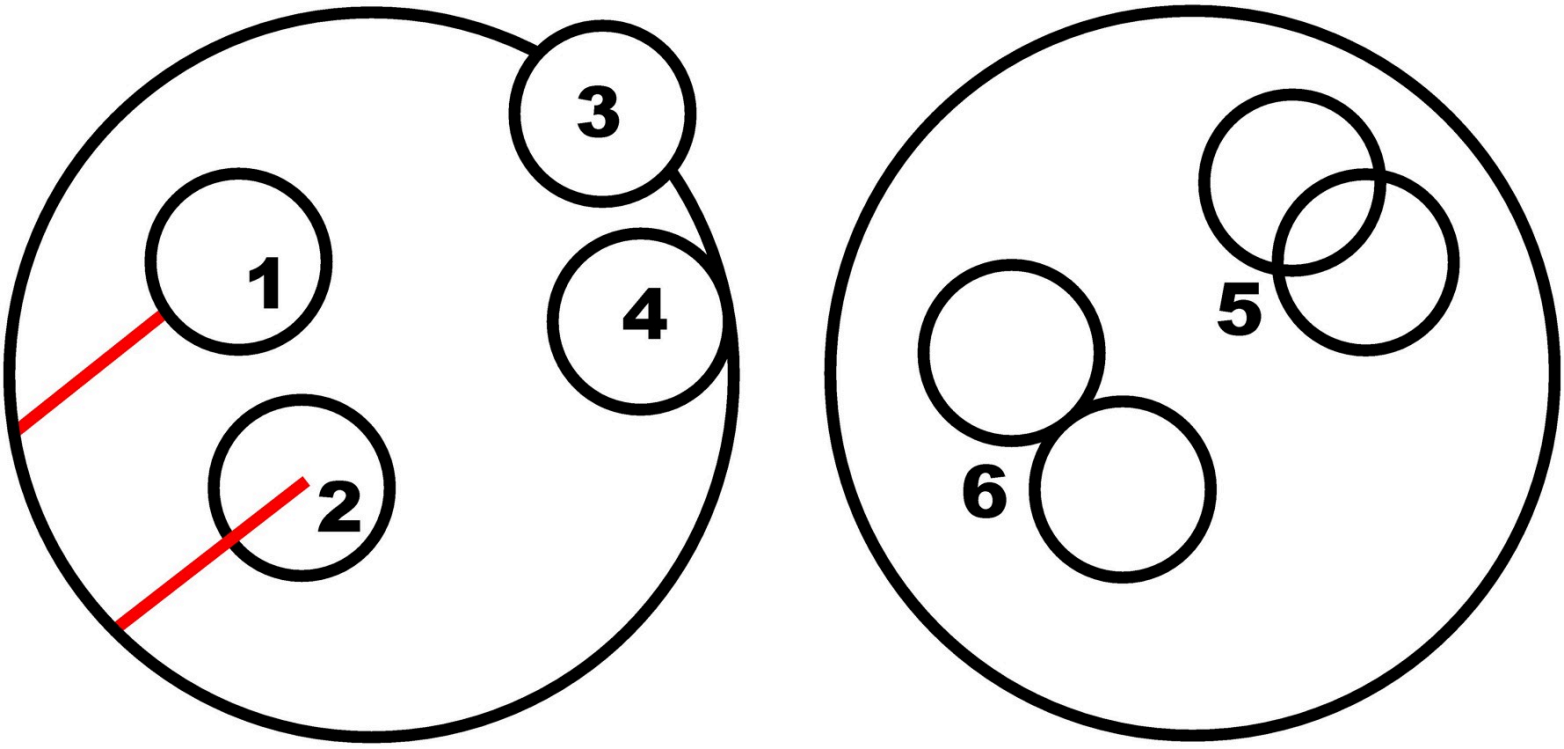
Clarification for all the different cases in Sect. 3.2.1. In #1, the peel-off is taken from the **surface** of the volume element. The other option is to start from the center of the volume element (#2). In #4 (**forced**), the volume element is fully inside the medium, whereas it can also be allowed to cross the surface of the medium (#3). #5 presents the options, in which volume elements can overlap sometimes (**probability**) or always (**allow**). In #6, the volume elements are not allowed (**deny**) to overlap.

There are different options for how to compute the distance Δ*τ*(*θ*, *ϕ*) for Eq 17. The distance can be computed from the surface of the volume element or its origin. Another implementation choice has to be made regarding how to handle the situations, in which the volume elements overlap, because the MC-RT utilizes infinitesimal points, whereas the exact computation entails volume elements. Also, if the MC-RT traces the point to the surface of the medium, the medium actually appears larger because the volume element crosses the surface of the medium (see #3 in Fig 5).

**Fig 6:**
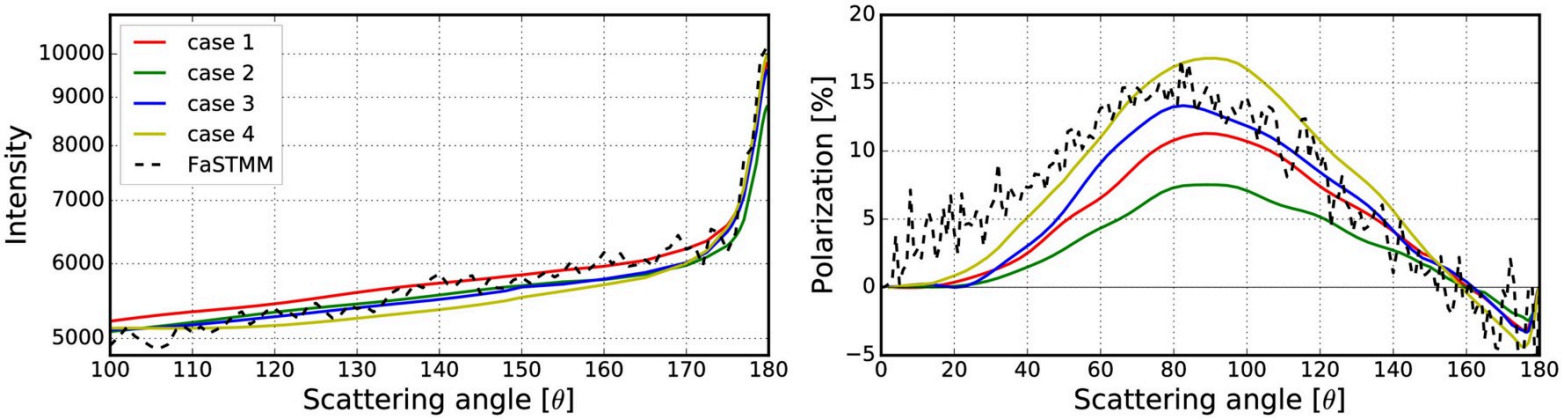
Phase function and polarization with varying sizes of the volume element. Comparison between the exact solution and the different cases (1–4) of how to account for overlap, position inside the random media, and the peel-off in the R^2^T^2^. See Table 1 for explanations.

**Fig 7:**
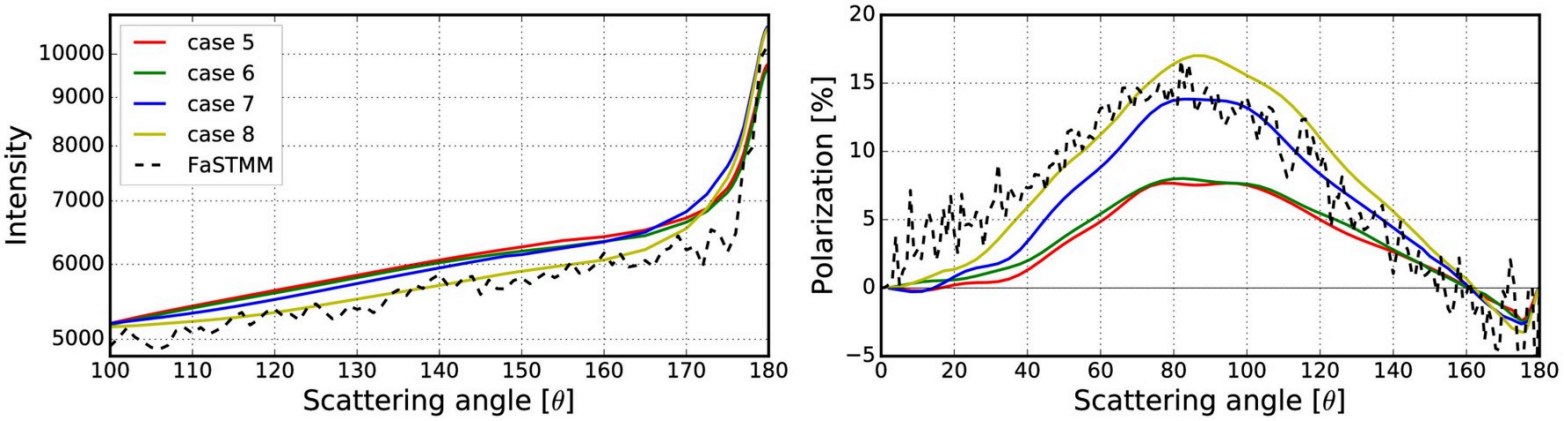
Same as Fig 6, but with different cases (5–8). See Table 1 for explanations.

**Fig 8:**
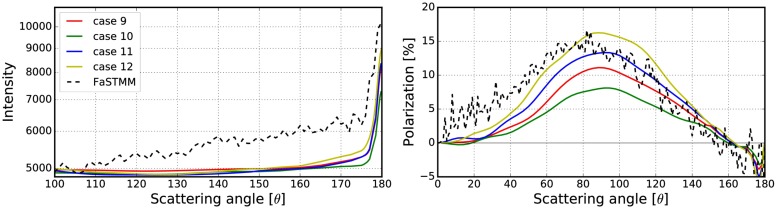
Same as Fig 6, but with different cases (9–12). See Table 1 for explanations.

Different combinations of these options are described in Table 1 and the results of studying the options are shown in Figs 6–8. The phase function and the degree of linear polarization are computed for a spherical random medium with size parameter *kR* = 139.69 and volume fraction *v* = 0.2. The random medium is composed of equisized scatterers with size parameter *ka* = 1.76 and refractive index *m* = 1.5 + 0.0001i. The size parameter of the volume element was *kR*_0_ = 10, with incoherent mean free path *kℓ* = 22.2 and mean albedo $\tilde{\omega }$ = 0.9985. The exact solution was derived with the FaSTMM by generating 16 samples and averaging each over 128 scattering planes. The outcome of the simulation is noisy due to a small number of generated samples. The data could be smoothened by increasing the number of samples but the trend would stay the same.

**Table 1:** Explanations for the cases shown in Figs 6–8.

	Overlap	Forced	Surface
**case 1**	Probability	x	x
**case 2**	Probability	x	
**case 3**	Probability		
**case 4**	Probability		x
**case 5**	Allow	x	x
**case 6**	Allow	x	
**case 7**	Allow		
**case 8**	Allow		x
**case 9**	Deny	x	x
**case 10**	Deny	x	
**case 11**	Deny		
**case 12**	Deny		x

**Overlap**, how to account for the overlapping volume elements. **Forced**, force the volume element to stay fully inside the medium. **Surface**, attenuation starts from the volume-element surface and not from the origin.

The overlap column refers to how to account for overlapping volume elements. The overlap **probability** means that the overlap is allowed sometimes by drawing a uniform random number *u* ∈ [0, 1[ and checking whether the condition

$$\begin{array}{c} u<1-\frac{V_{\text{intersection}}}{V}. \end{array}$$ (21)

is true. In Eq 21, *V* is the volume of the volume element and *V*_intersection_ is the intersection of the two overlapping volume elements. Overlap **allow** means that the volume elements are allowed to overlap always while in **deny**-cases that is never allowed (see #5 and #6 in Fig 5).

In rows in which the **forced** column is ticked, the volume elements are fully forced to stay inside the medium so that the surface of the volume element does not cross the surface of the medium (see #3 and #4 in Fig 5). A tick in the **surface** column means that the exponential attenuation is computed from the surface of the volume element to the surface of the random medium. The other choice is to start from the origin of the volume element (see #1 and #2 in Fig 5).

Figs 6–8 show that the best results are obtained polarization-wise in the cases 3, 7, and 11. In all of these, the polarization is close to the exact solution whereas in the case 3 the phase function is also close. By never allowing the overlap, the CB produces a steeper polarization surge, lower phase function, and narrower backscattering spike compared to the cases, in which the overlap is always allowed. Thus the correct way to account for the overlap must be somewhere between **deny** and **allow**. The use of **forced** and **surface** are not producing desirable results and are thus omitted for simplicity in the rest of the paper. From now on, everything will be studied using the option **probability**.

#### 3.2.2 Volume-element size

**Fig 9:**
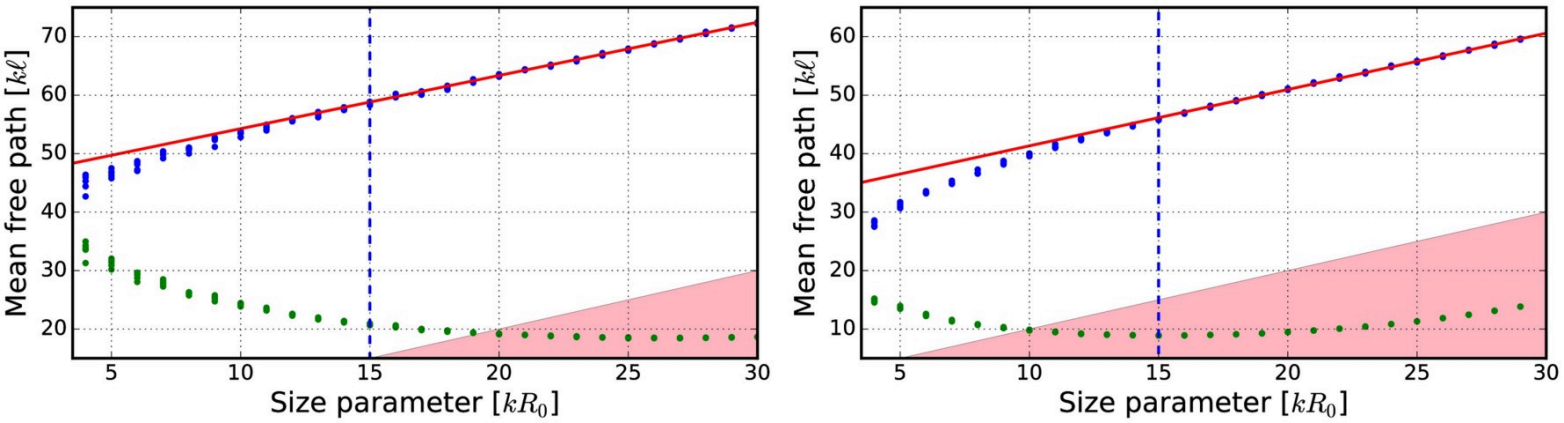
Mean free path as a function of the volume-element size parameter. Blue points are incoherent mean free paths (Eq 11) and green points are mean free paths computed by using the free-space extinction cross sections. The spherical particles have *m* = 1.31 and *ka* = 2.0. Volume fractions *v* = 0.125 (left), and *v* = 0.25 (right). The fitted line is shown in red and the area *ℓ* < *R*_0_ is highlighted.

**Fig 10:**
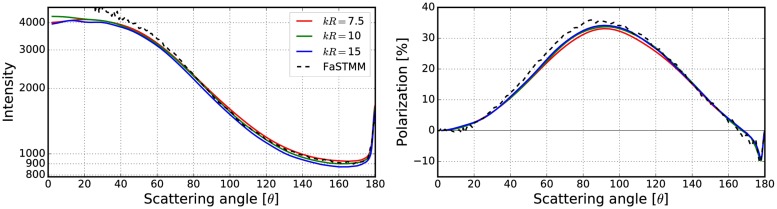
Intensity (left) and degree of linear polarization (right) for the spherical medium with *kR* = 100, and *v* = 0.125. The spherical particles have *m* = 1.31 and *ka* = 2.0. The volume-element properties are in Fig 9 (left).

**Fig 11:**
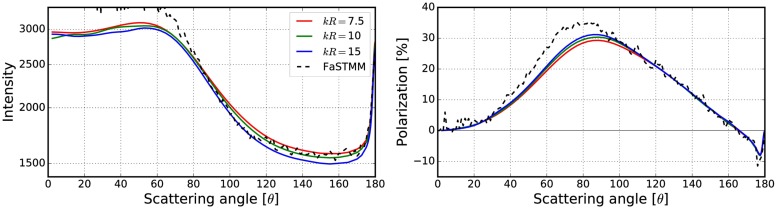
As in Fig 10 for *kR* = 100, *v* = 0.25, *m* = 1.31, and *ka* = 2.0. The volume-element properties are in Fig 9 (right).

**Fig 12:**
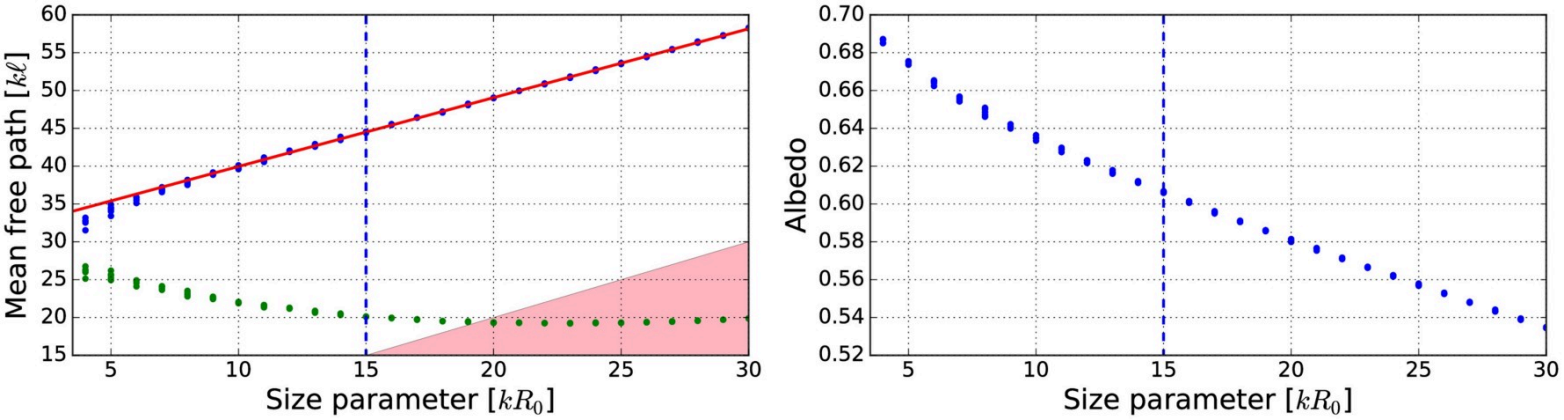
Mean free path (left) and albedo (right) as a function of the volume-element size parameter. In the left, blue points depict incoherent mean free paths (Eq 11) and green points the mean free paths obtained by using free-space extinction cross sections. The spherical particles have *m* = 1.31 + 0.03i and *ka* = 2.0. The volume fraction is *v* = 0.125. The fitted line is shown in red and the area *ℓ* < *R*_0_ is highlighted.

**Fig 13:**
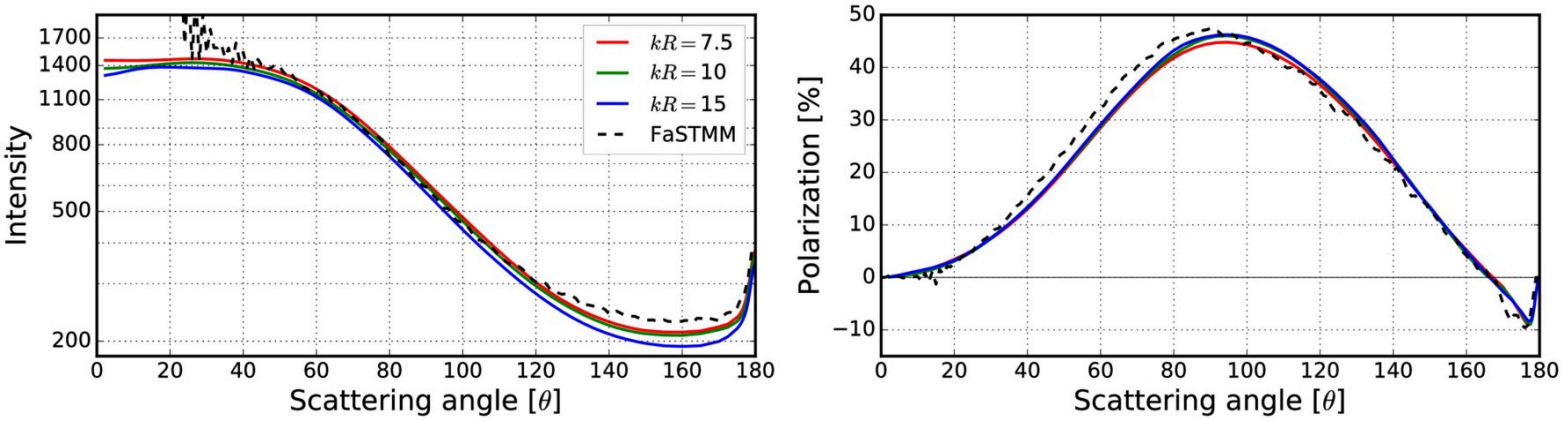
As in Fig 10 for *kR* = 100, *v* = 0.125, *m* = 1.31 + 0.03i, and *ka* = 2.0. The volume-element properties are in Fig 12.

**Fig 14:**
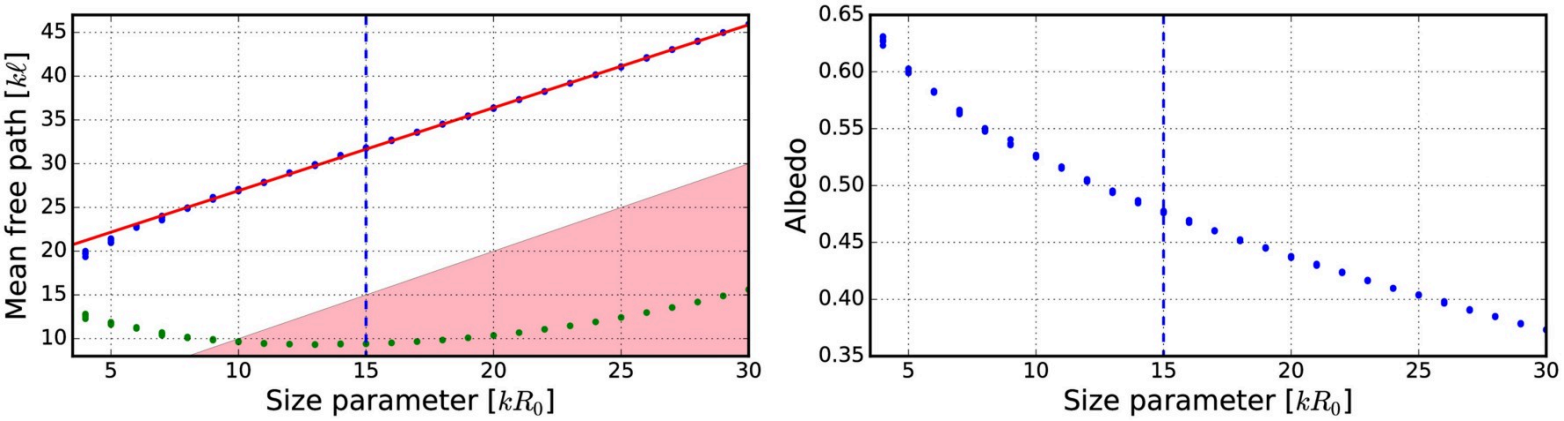
As in Fig 12 for *m* = 1.31 + 0.03i, *ka* = 2.0, and *v* = 0.25.

**Fig 15:**
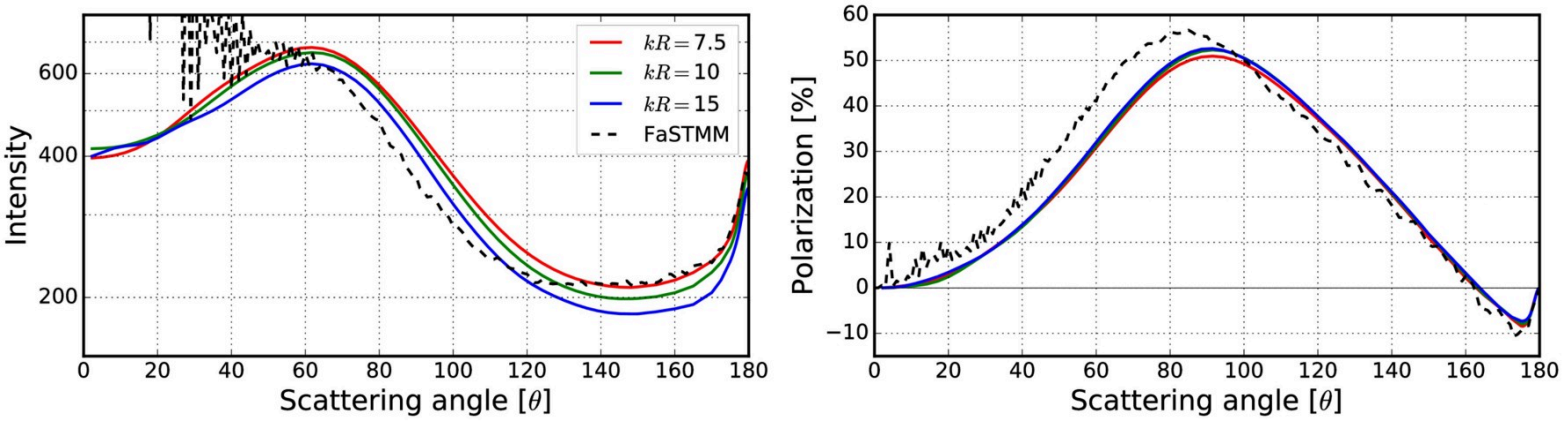
As in Fig 10 for *kR* = 100, *v* = 0.25, *m* = 1.31 + 0.03i, and *ka* = 2.0. The volume-element properties are in Fig 14.

**Fig 16:**
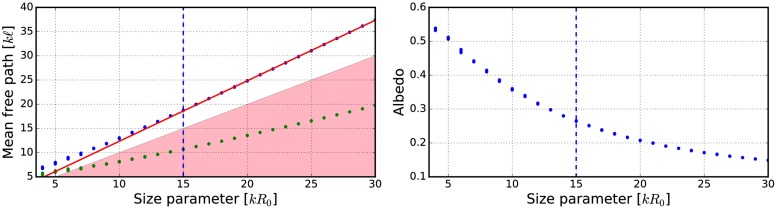
As in Fig 12 for *m* = 2.0 + 0.2i, *ka* = 2.0, and *v* = 0.125.

**Fig 17:**
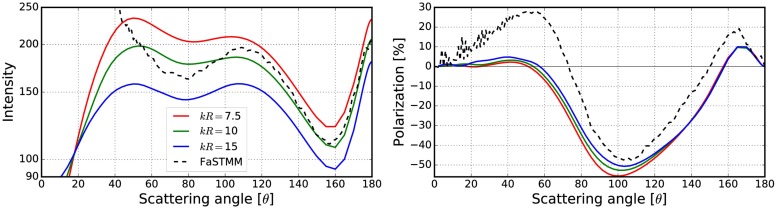
As in Fig 10 for *kR* = 100, *v* = 0.125, *m* = 2.0 + 0.2i and *ka* = 2.0. The volume-element properties are in Fig 16.

**Fig 18:**
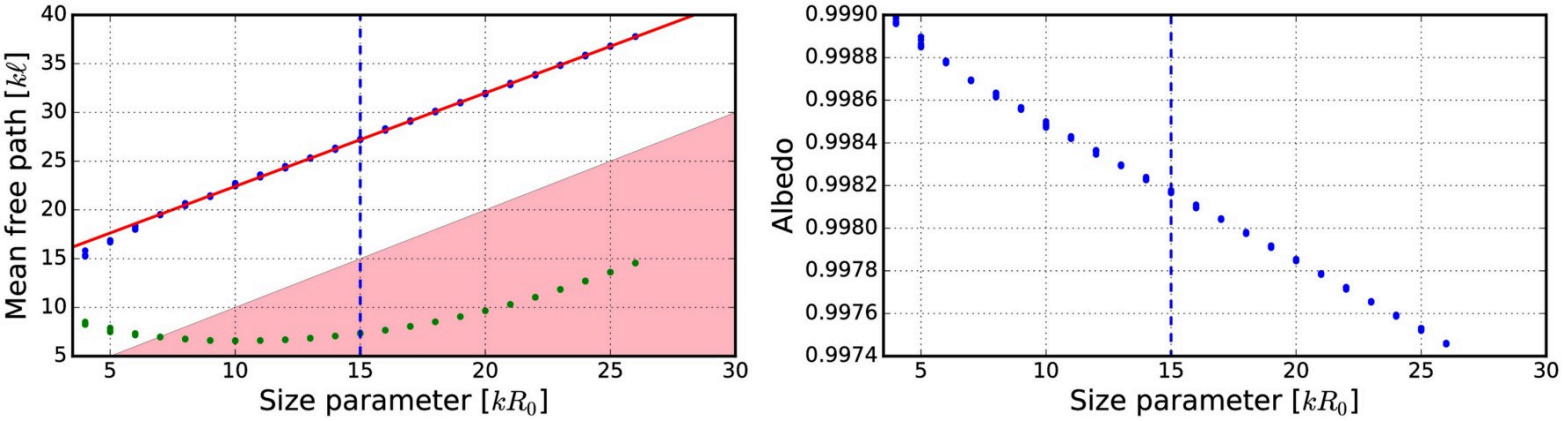
As in Fig 12 for *m* = 1.5 + 0.0001i, *ka* = 1.76, and *v* = 0.2.

**Fig 19:**
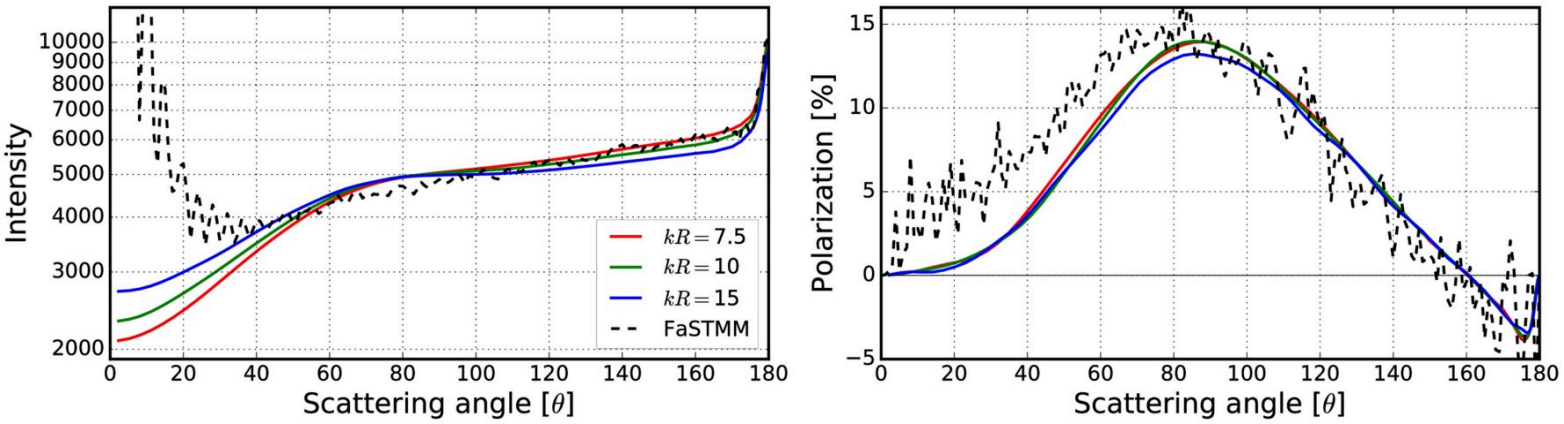
As in Fig 10 for *kR* = 136.69, *v* = 0.2, *m* = 1.5 + 0.0001i, and *ka* = 1.76. The volume-element properties are in Fig 18.

Currently, the size of the volume element is not fixed and thus the effects of the volume-element sizes on the mean free path, the albedo, and the final output of the R^2^T^2^ were studied (Table 2, Figs 9–19). The size parameter of the volume element was varied from *kR*_0_ = 4 to *kR*_0_ = 30 with different parameters (see Table 2) and the properties of the incoherent volume element were solved. The non-absorbing cases are the same as presented in [19] (see Figs 10 and 11).

**Table 2:** Parameters used in Figs 9–19.

Figs	*kR*	*m*	*v*	*ka*	*c*	*b*	Best (*kR*_0_)
9,10	100	1.31 + 0.0i	0.125	2.00	0.910	45.17	10.0
9,11	100	1.31 + 0.0i	0.25	2.00	0.963	31.70	10.0
12,13	100	1.31 + 0.03i	0.125	2.00	0.910	30.86	7.5
14,15	100	1.31 + 0.03i	0.25	2.00	0.948	17.42	7.5
16,17	100	2.0 + 0.2i	0.125	2.00	1.250	-0.16	10
18,19	136.69	1.5 + 0.0001i	0.2	1.76	0.957	12.84	10

Parameters (size parameter of the medium *kR*, refractive index *m*, volume fraction *v*, size parameter of the scatterer *ka*) and coefficients (slope *c*, constant *b*) used in Figs 9–19.

The mean free paths and albedos were studied in Figs 9, 12, 14, 16, and 18. In these figures, the area *ℓ* < *R*_0_ is highlighted to show where the mean free path is too short compared to the size of the volume element. In the cases in which absorption is present, the albedo was studied as well. The mean free path computed by using the free-space extinction cross section (see Eqs 11 and 10) is plotted with green points. For each case, the exact simulation was computed with the FaSTMM by averaging over 96 samples and 128 scattering planes (*kR* = 100), while the *kR* = 136.69 case was computed using only 16 samples. The R^2^T^2^ results were computed using size parameters *kR*_0_ = (7.5, 10, and 15) from which the best sizes were selected (See column “Best (*kR*_0_)” in Table 2) and used in Sect 4.

The albedo does not show any asymptotic behaviour at least between the size parameters *kR*_0_ = 4–30. A linear trend (*kℓ* = *ckR*_0_ + *b*) can be found by plotting the mean free paths as a function of the size parameter. The linear equation was fitted using the linear least squares method for the mean free paths obtained with *kR*_0_ > 15 (marked with blue vertical line). The fitted coefficients (*c* and *b*) are shown in Table 2. The linear trend testifies to the fact that the incoherent extinction cross section becomes proportional to the projected area of the volume element, that is, a typical limit of geometric optics. Reaching this region can indicate that the volume element is too large to be used, while the lower limit can be drawn from the condition that the volume element must be large enough to contain a large number of particles. [19]. It is also interesting that, in Fig 16, the mean free paths approach a linear trend from above instead of approaching the trend from below as in Figs 9, 12, 14, and 18.

The effect of the size parameter on the outcome of the R^2^T^2^ is shown in Figs 10, 11, 13, 15, 17, and 19. The overall trend is that the increasing volume-element size causes lower intensities, and stronger absorption if no absorption is present. With the absorption, the increase of the size parameter causes lower polarization. The best match does not seem to coincide with any particular point in the mean free path function so it is hard to point out any underlying rule. Nevertheless, *kR*_0_ = 7.5–10 produces the best phase functions and these are highlighted in Table 2 and used in the Sect 4. One can argue convergence with increasing size parameter by using different rules discussed in Sect 3.2.1, e.g., allowing overlap always.

The choice of the correct configuration is hard to make due to multiple parameters affecting the results, such as, the size of the volume element, direct transmission (Sect 3.1.1), refractive index, and the choices of underlying rules (Sect 3.1.3). Especially the direct transmission should be handled carefully, because it dictates how much intensity is left for diffuse scattering and absorption. This part should not depend on the size of the volume element, but in the current implementation, the rate of the direct transmission is affected by it. This is not a problem especially in the case of Fig 19, because the amount of direct transmission was only about 1–2%. In smaller media with longer mean free paths, the rate of the direct transmission will be more prominent.

### 3.3 Precomputations

#### 3.3.1 Incoherent *T*-matrix

In order to obtain the incoherent scattering properties of the volume element, the coherent light-scattering characteristics need to be solved for. We approximate the coherent *T*-matrix (Eq 7) by averaging *T*_*i*_ matrices over *L* randomly packed volume-element realizations as

$$\begin{array}{c} T^{c}=\frac{1}{L}\sum_{i=1}^{L}T_{i}. \end{array}$$ (22)

Now this coherent *T*-matrix can be used to compute the incoherent scattered field from Eq 5. The coherent *T*-matrix is composed of ensemble-averaged values which means that it is constant and needs to be computed once.

This coherent *T*-matrix can be then used to generate multiple incoherent $T_{i}^{\text{ic}}$-matrices

$$\begin{array}{c} T_{i}^{\text{ic}}=T_{i}-T^{c},\;\;\;\forall i=1,...,L. \end{array}$$ (23)

which can be used to compute the electric field coefficients for the electric field without the need to compute the electric field in Eq 2. Another advantage of this form comes from the fact that multiple *T*-matrices have to be computed anyway for Eq 22, so these *T*-matrices can be re-used to extract the *T*^ic^-matrices without additional runtime costs. The other precomputed parameters are the incoherent mean free path and albedo (see Eqs 11 and 12). As demonstrated in [20], the current implementation of the R^2^T^2^ is general in a sense that the user can use any kind of volume elements provided that the user is able to produce multiple $T_{i}^{\text{ic}}$-matrices.

#### 3.3.2 Random packing of spheres

The incoherent *T*-matrices are computed with a modified version of the FaSTMM (see S2 Source Code), which is designed to compute the incoherent volume-element characteristics. The program incorporates a random packing algorithm for spheres and produces *T*-matrices.

**Fig 20:**
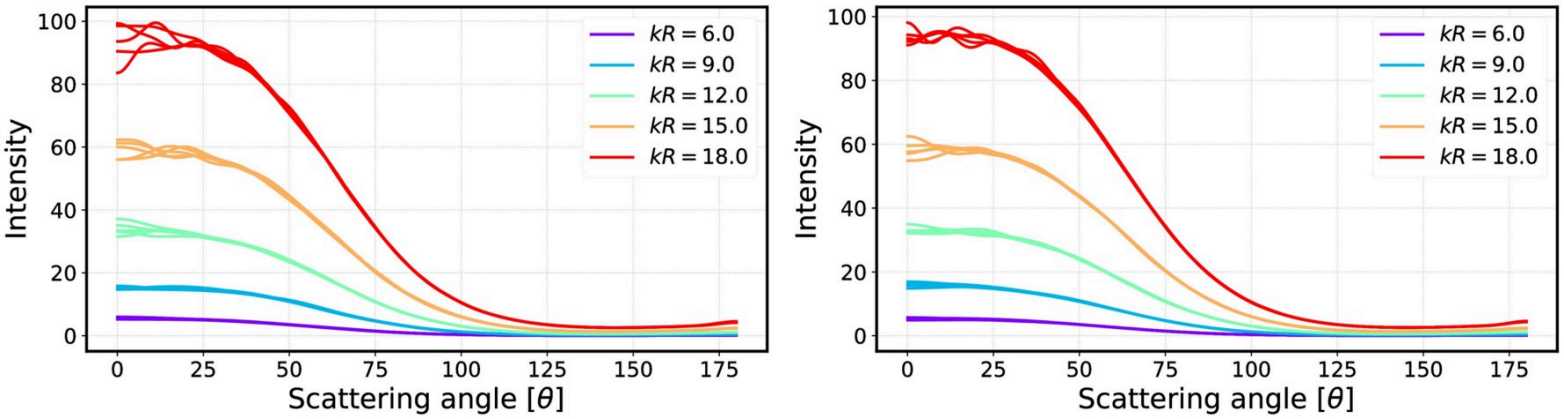
Multiple phase functions computed for varying size parameters. Results obtained by averaging over 512 (left) and 1024 (right) samples.

**Fig 21:**
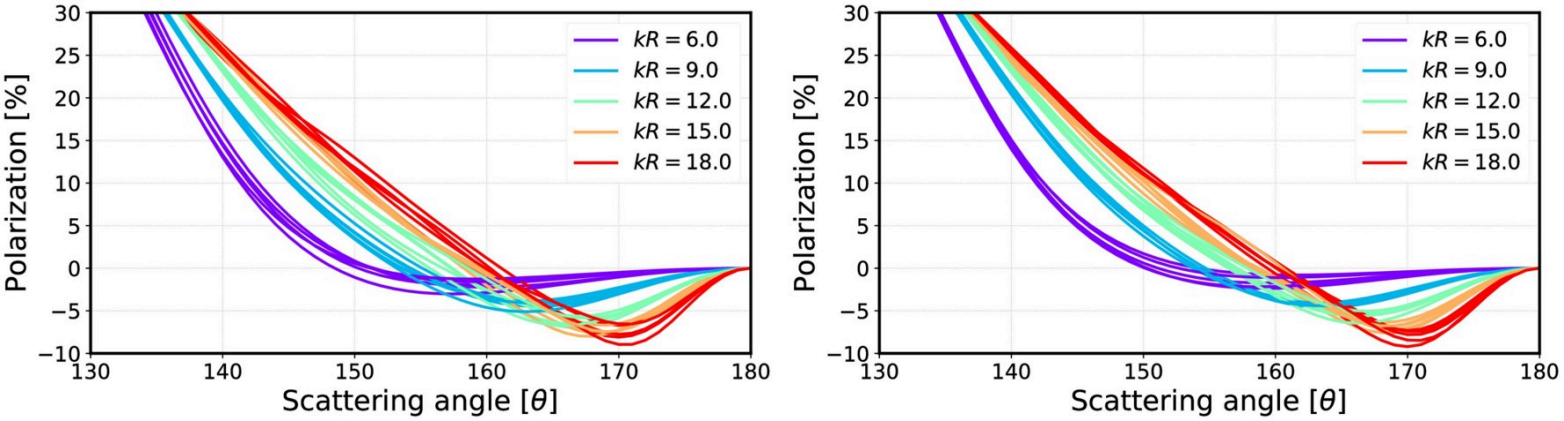
Same as Fig 20 but for the degree of linear polarization.

An important aspect of how to compute the incoherent *T*-matrices is the packing algorithm. We generate a large periodic box from which a spherical volume element is drawn. The periodic box is used to ensure correct statistical sampling of volume elements. In Fig 20, phase functions are computed multiple times for different volume elements of varying size parameters and plotted as a function of size parameter using 512 and 1024 configurations. In Fig 21, the corresponding linear polarizations are plotted to show fluctuations in the backscattering angles between the samples. The number of particles inside the box was 10^5^. Fig 20 shows that the forward-scattering part fluctuates in the same way as in [18], in which the fluctuation was handled empirically.

## 4 Results

**Fig 22:**
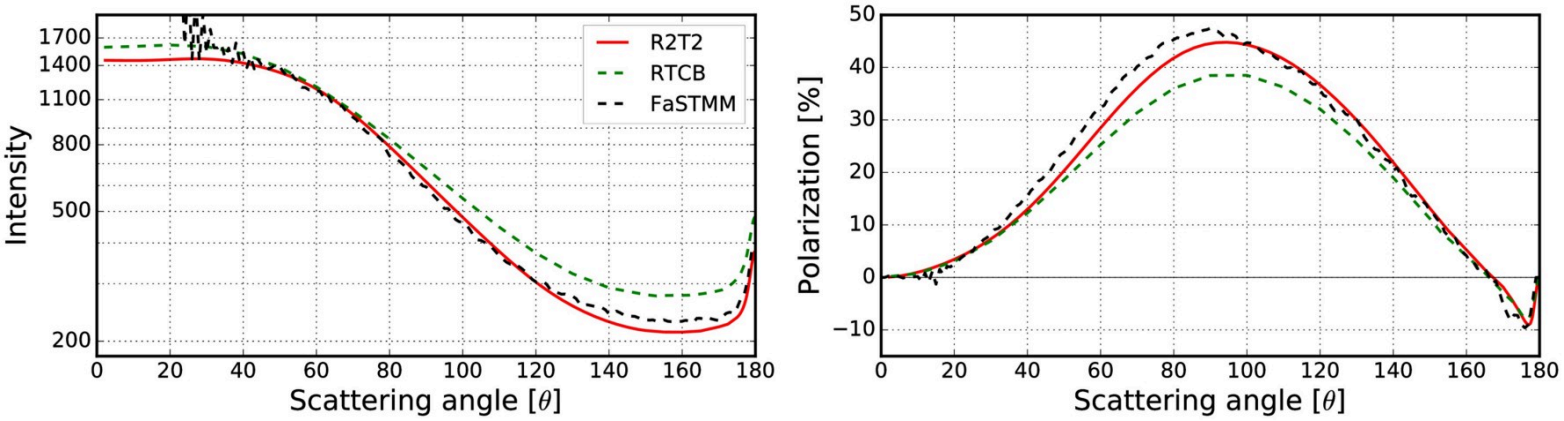
Intensity (left) and degree of linear polarization (right) for the spherical medium with *kR* = 100, and *v* = 0.125. The spherical particles have *m* = 1.31 + 0.03i, and *ka* = 2.0. The volume-element size is *kR*_0_ = 7.5.

**Fig 23:**
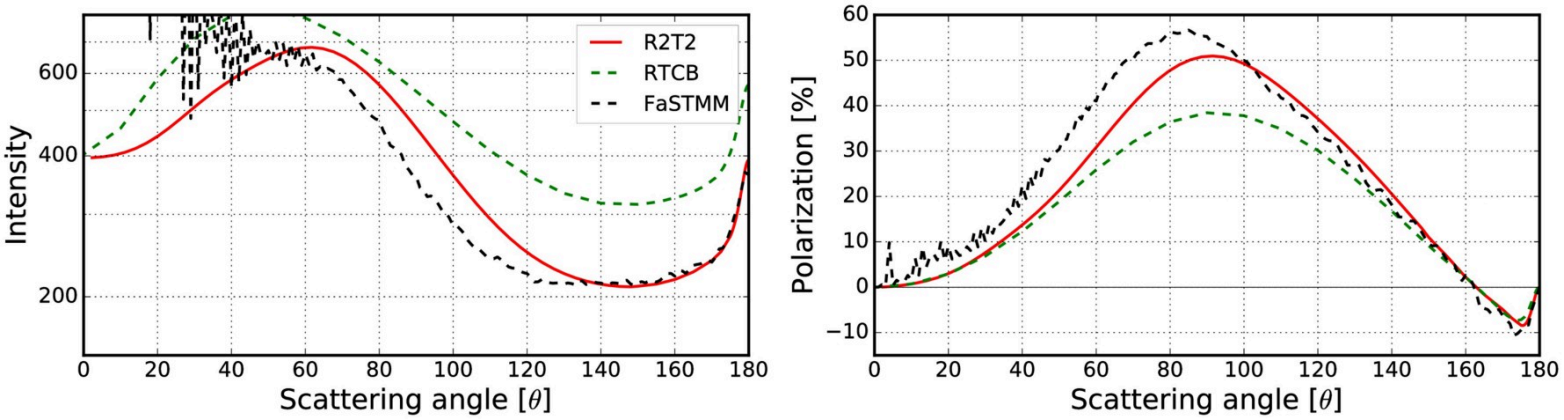
As in Fig 22 for *kR* = 100, *v* = 0.25, *m* = 1.31 + 0.03i, *ka* = 2.0, and *kR*_0_ = 7.5.

**Fig 24:**
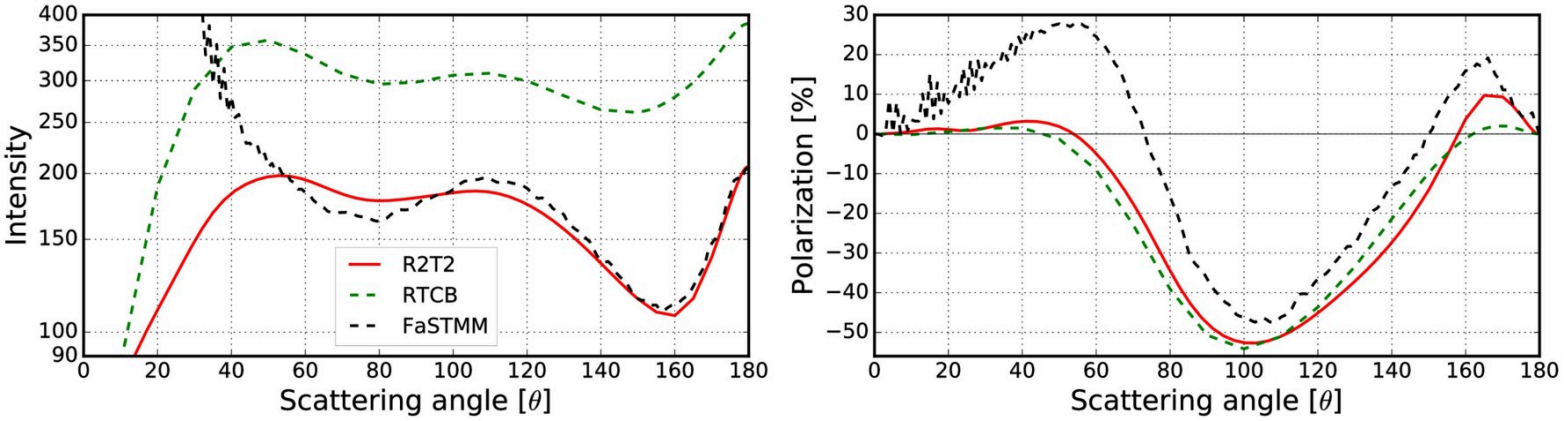
As in Fig 22 for *kR* = 100, *v* = 0.125, *m* = 2.0 + 0.2i, *ka* = 2.0, and *kR*_0_ = 10.

**Fig 25:**
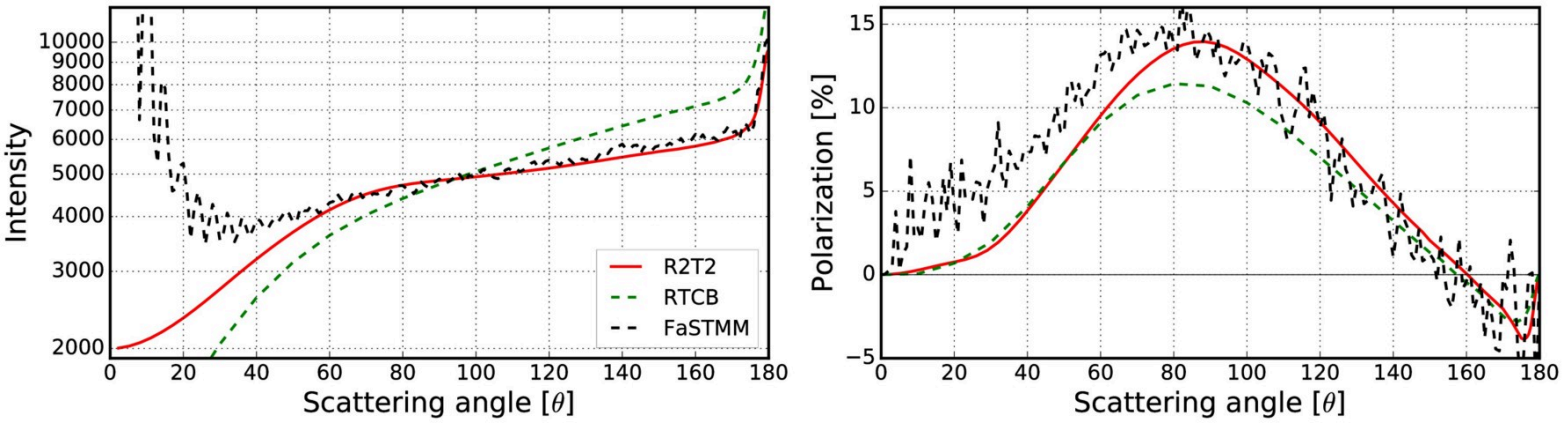
As in Fig 22 for *kR* = 136.69, *v* = 0.2, *m* = 1.5 + 0.0001i, *ka* = 1.76, and *kR*_0_ = 10.

**Fig 26:**
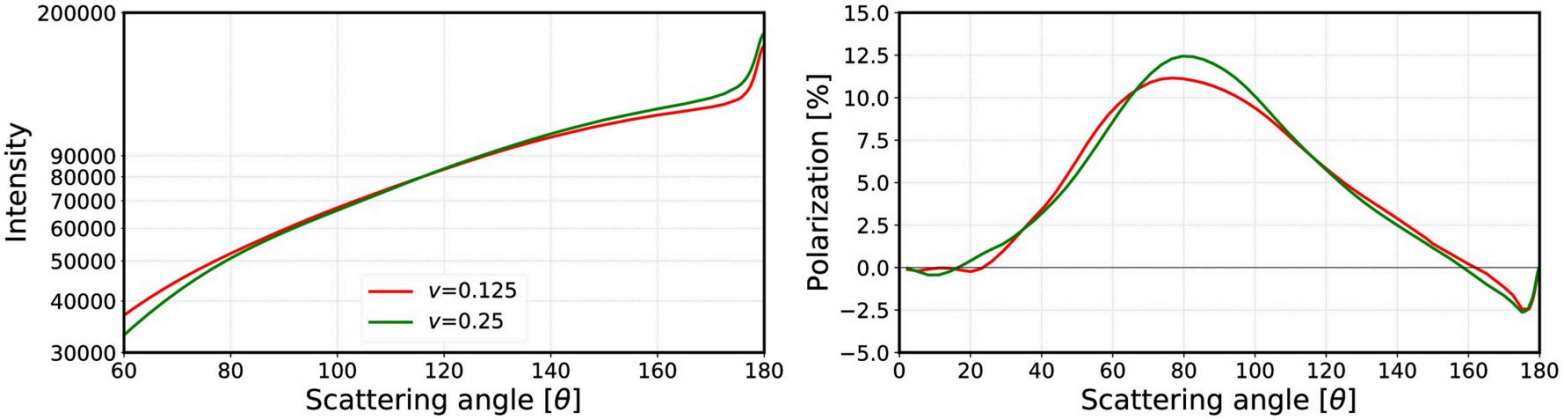
As in Fig 22 for *kR* = 500, *m* = 2.0 + 0.2i, *ka* = 2.0, and *kR*_0_ = 10. The volume fractions are *v* = 0.125 and *v* = 0.25.

To expand the validations in [19] which considered only non-absorbing cases, the best results obtained in Sect 3.2.2 are compared against the RT-CB. The phase function and the degree of linear polarization were computed for different cases with the R^2^T^2^, RT-CB [31], and the FaSTMM [22] (see Figs 22–26). The parameters are shown in Table 3. Also, scattering from larger *kR* = 500 random media with *v* = 0.125 and *v* = 0.25 composed of spherical particles (*ka* = 1.76 and *m* = 1.5 + 0.0001i) were computed (see Fig 26) to show capabilities of the R^2^T^2^ and to provide results which can be used to validate our method. Contrary to larger cases *kR* = 10000 [19] or *kR* = 1.2 × 10^13^ [20], future numerical validation should be possible for the *kR* = 500 case with exact methods. The phase functions are normalized so that the integration over the full solid angle produces the scattering cross section.

**Table 3:** Parameters for Figs 22–26.

Fig	*kR*	*m*	*v*	*ka*	*kℓ*,r^2^t^2^	$\bar{\omega }$,r^2^t^2^	*kℓ*,rt-cb	$\bar{\omega }$,rt-cb
22	100.0	1.31 + 0.03i	0.125	2.00	38.1	0.65	27.6	0.74
23	100.0	1.31 + 0.03i	0.25	2.00	24.4	0.56	13.8	0.74
24	100.0	2.0 + 0.2i	0.125	2.00	10.3	0.43	5.5	0.63
25	136.69	1.5 + 0.0001i	0.2	1.76	22.2	0.999	9.4	0.999
26	500.0	1.5 + 0.0001i	0.125	1.76	26.6	0.999		
26	500.0	1.5 + 0.0001i	0.25	1.76	21.2	0.998		

Parameters for Figs 22–26. $\bar{\omega }$ and *kℓ* are the albedo and mean free path used in the R^2^T^2^/RT-CB.

The exact computations took around 0.7 CPU years. Computing the incoherent *T*-matrices consumed about 24–72 CPU hours, whereas the R^2^T^2^ consumed around 96–192 CPU hours to compute the *kR* = 100 cases. The larger *kR* = 500 cases were computed using 0.4 CPU years.

**Fig 27:**
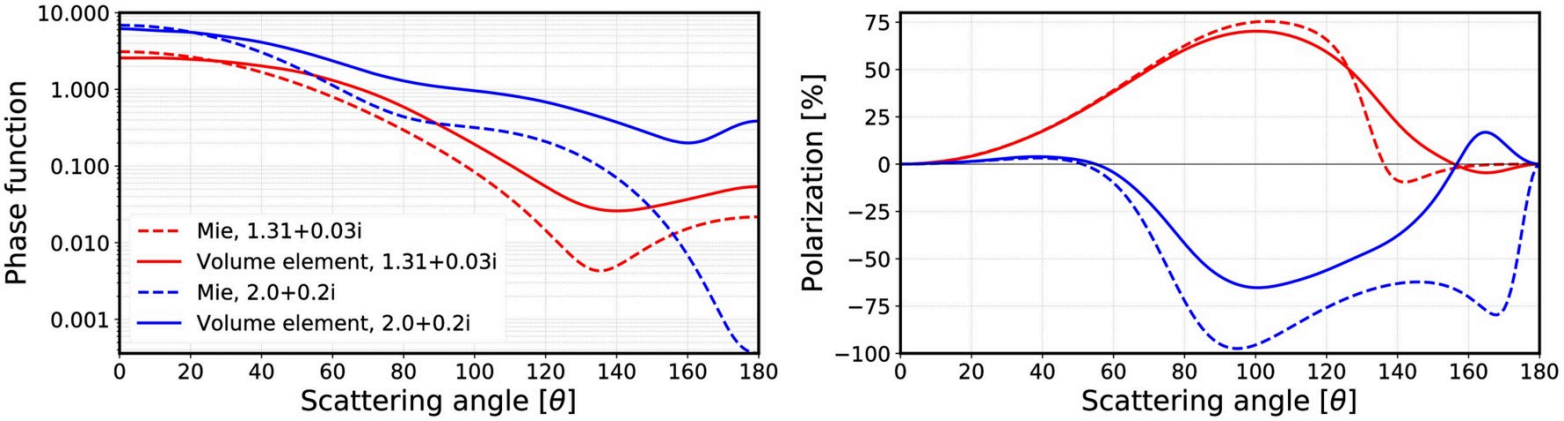
Mie spheres (dashed lines) compared to averaged incoherent volume elements (*kR*_0_ = 10 and *v* = 0.125, solid lines). The phase functions (left) of the *m* = 2.0 + 0.2i cases are multiplied by 2 for better display. The phase function yields 4*π* when integrated over the full solid angle.

Fig 27 highlights the differences between inputs by comparing the incoherent volume element (R^2^T^2^) and Mie spheres [12] (RT-CB).

## 5 Discussion

The R^2^T^2^ succeeds in producing results similar to the FaSTMM even in the absorbing cases (see Figs 22–25), consuming less computing resources than the FaSTMM. There are only minor differences in all of the studied cases and the results are significantly more accurate than those with the RT-CB. The volume element should be allowed to overlap always or sometimes but the option of denying it cannot be the correct way as was seen in Figs 6–8.

The size of the volume elements should be studied further in the future (e.g., studying the behaviour of light-scattering characteristics of incoherent volume elements as was done in Sect 3.2.2). The size of the volume element affects the intersection of the volume elements which is a strong source of error in dense media. The next scattering is likely to take place inside the current volume element and it should be allowed as was seen in Sect 3.2.1. The problem is that the rigorous computation does not allow overlapping volume elements. Another source of error is the assumed exponential attenuation scheme present. Each ray is attenuated exponentially which is valid for sparse media, but might be too simple for dense media. A better model should be developed and implemented into the R^2^T^2^.

There are also some differences which are independent of the used overlap option and the size of the volume element. The polarization peak shifts more to the forward-scattering direction when the system is dense (compare Figs 22 and 23, which was also visible in Figs 10 and 11). This might be due to coherent field reflections and refractions not included in the R^2^T^2^. Also the level of the phase function seems to be higher in the forward scattering angles, although, some of it is due to diffraction. The phase function in the forward scattering direction seems to be shifted to the forward scattering angles (see Fig 23). In [21], the coherent reflections and refractions were taken into account by employing geometric optics for the coherent field in combination with the incoherent volume elements as diffused scatterers in the RT method. Nonetheless, it is not trivial to implement such a correction in the R^2^T^2^ and it omitted in the present work.

Incoherent volume elements smoothened the scattering characteristics of the Mie spheres, but there are still some drastic changes especially in the polarization functions as seen in Fig 21. The volume element (*m* = 2.0 + 0.2i) has strong positive backscattering polarization, which can also be seen in the results from the R^2^T^2^ (compare to Fig 25). The Mie sphere does not have this feature, although the RT-CB is able to produce slightly positive polarization. It is notable that the polarization of the incoherent volume element has similarities to the polarization from the R^2^T^2^ (compare Fig 21 to Figs 22 and 25) but it is not possible to make similar conclusions from the phase function.

## 6 Conclusions

In the paper, the radiative transfer with reciprocal transactions R^2^T^2^ was presented in detail and some of the implementation choices were studied. The tests were completed using spherical scatterers, but the R^2^T^2^ is capable of using volume elements of nonspherical scatterers provided that the user can supply multiple incoherent *T*-matrices. The R^2^T^2^ was compared to the asymptotically exact FaSTMM and the results are promising especially in the backscattering angles. There are still some differences for extremely dense random media with high real or imaginary parts of the refractive index. In such cases, the mean free path is close to the size of the volume element, and therefore, scattering distances are short and the volume elements often intersect. Thus, we expect to obtain improved results if the intersecting elements are considered more rigorously. The size dependency should be studied further but this would require a study of the attenuation inside the media. We have shown that the small size for the volume element produces relatively accurate results. But finding the criteria for the volume element size should be studied to make the R^2^T^2^ more robust.

## Supporting information

S1 Source Code Source code for the R^2^T^2^. A link to the latest version: https://bitbucket.org/planetarysystemresearch/r2t2_pub.
(ZIP)

S2 Source Code Source code for the incoherent volume element generator. A link to the latest version: https://bitbucket.org/planetarysystemresearch/ivegen_pub.
(ZIP)
