# Supplementary material for: Radiative transfer with reciprocal transactions: Numerical method and its implementation
Source: PLoS One. 2019 Jan 8;14(1):e0210155. doi: 10.1371/journal.pone.0210155 (PMC6324827; doi:10.1371/journal.pone.0210155)
Supplement: S1 Source Code — A link to the latest version: https://bitbucket.org/planetarysystemresearch/r2t2_pub. (ZIP) [file pone.0210155.s001.zip › r2t2_pub/src/dsfmt/dsfmt/html/union_w128___t.html]

dSFMT: W128\_T Union Reference


|  |
| --- |
| dSFMT  2.2 |

- Main Page
- Data Structures
- Files

- Data Structures
- Data Fields

Data Fields

W128\_T Union Reference

128-bit data structure
More...

`#include <dSFMT.h>`

|  |  |
| --- | --- |
| Data Fields | |
| uint64\_t | u [2] |
| uint32\_t | u32 [4] |
| double | d [2] |

---

## Detailed Description

128-bit data structure

---

## Field Documentation

|  |
| --- |
| double W128\_T::d[2] |

Referenced by convert\_c0o1(), convert\_o0c1(), convert\_o0o1(), dsfmt\_genrand\_close1\_open2(), and dsfmt\_genrand\_open\_open().

|  |
| --- |
| uint64\_t W128\_T::u[2] |

Referenced by convert\_o0o1(), do\_recursion(), initial\_mask(), and period\_certification().

|  |
| --- |
| uint32\_t W128\_T::u32[4] |

Referenced by dsfmt\_chk\_init\_by\_array(), and dsfmt\_chk\_init\_gen\_rand().

---

The documentation for this union was generated from the following file:

- dSFMT.h


---

Generated on Fri Jun 29 2012 16:17:32 for dSFMT by  

 1.8.0
