# Supplementary material for: Radiative transfer with reciprocal transactions: Numerical method and its implementation
Source: PLoS One. 2019 Jan 8;14(1):e0210155. doi: 10.1371/journal.pone.0210155 (PMC6324827; doi:10.1371/journal.pone.0210155)
Supplement: S1 Source Code — A link to the latest version: https://bitbucket.org/planetarysystemresearch/r2t2_pub. (ZIP) [file pone.0210155.s001.zip › r2t2_pub/src/dsfmt/dsfmt/html/struct_d_s_f_m_t___t.html]

dSFMT: DSFMT\_T Struct Reference


|  |
| --- |
| dSFMT  2.2 |

- Main Page
- Data Structures
- Files

- Data Structures
- Data Fields

Data Fields

DSFMT\_T Struct Reference

the 128-bit internal state array
More...

`#include <dSFMT.h>`

|  |  |
| --- | --- |
| Data Fields | |
| w128\_t | status [DSFMT\_N+1] |
| int | idx |

---

## Detailed Description

the 128-bit internal state array

---

## Field Documentation

|  |
| --- |
| int DSFMT\_T::idx |

Referenced by dsfmt\_chk\_init\_by\_array(), dsfmt\_chk\_init\_gen\_rand(), dsfmt\_genrand\_close1\_open2(), and dsfmt\_genrand\_open\_open().

|  |
| --- |
| w128\_t DSFMT\_T::status[DSFMT\_N+1] |

Referenced by dsfmt\_chk\_init\_by\_array(), dsfmt\_chk\_init\_gen\_rand(), dsfmt\_gen\_rand\_all(), dsfmt\_genrand\_close1\_open2(), dsfmt\_genrand\_open\_open(), gen\_rand\_array\_c0o1(), gen\_rand\_array\_c1o2(), gen\_rand\_array\_o0c1(), gen\_rand\_array\_o0o1(), initial\_mask(), and period\_certification().

---

The documentation for this struct was generated from the following file:

- dSFMT.h


---

Generated on Fri Jun 29 2012 16:17:32 for dSFMT by  

 1.8.0
