# Supplementary material for: Radiative transfer with reciprocal transactions: Numerical method and its implementation
Source: PLoS One. 2019 Jan 8;14(1):e0210155. doi: 10.1371/journal.pone.0210155 (PMC6324827; doi:10.1371/journal.pone.0210155)
Supplement: S1 Source Code — A link to the latest version: https://bitbucket.org/planetarysystemresearch/r2t2_pub. (ZIP) [file pone.0210155.s001.zip › r2t2_pub/src/dsfmt/dsfmt/html/index.html]

dSFMT: Main Page


|  |
| --- |
| dSFMT  2.2 |

- Main Page
- Data Structures
- Files

dSFMT Documentation

This is double precision SIMD oriented Fast Mersenne Twister pseudorandom number generator (dSFMT). This program is based on the IEEE Standard for Binary Floating-Point Arithmetic (ANSI/IEEE Std 754-1985) format.

dSFMT ver. 2.xx is completely different from dSFMT ver. 1.xx. The recursion formula is changed. And the output sequences are changed.

This version uses **structure** of C language. Don't use different **DSFMT\_MEXP** for compiling dSFMT.c and your program.

This Project provides pseudorandom number generators of various Mersenne Prime Period: from 2521-1 to 2216091-1.

To get dSFMT which has the period of 2521-1, you have to specify -DMEXP=521 as a compile option. For example,

```
 gcc -DDSFMT_MEXP=521 -msse2 -DHAVE_SSE2 -c dSFMT.c
```

will make dSFMT which has the period of 2521-1 and is optimized for SSE2.

See How to compile to compile your program with SFMT optimized for SIMD.

- void dsfmt\_init\_gen\_rand() initializes the generator with a 32-bit integer seed.
  - void dsfmt\_init\_by\_array() initializes the generator with an array of 32-bit integers as the seeds.
  - const char \* dsfmt\_get\_idstring() returns the IDSTRING which identify the generator.
  - int dsfmt\_get\_min\_array\_size() returns the minimum size of array used for **fill\_array** functions.
  - inline double dsfmt\_genrand\_close1\_open2() generates and returns a double precision pseudorandom number which distributes uniformly in the range [1, 2). This is the primitive and faster than generating numbers in other ranges.
  - inline double dsfmt\_genrand\_close\_open() generates and returns a double precision pseudorandom number which distributes uniformly in the range [0, 1).
  - inline double dsfmt\_genrand\_open\_close() generates and returns a double precision pseudorandom number which distributes uniformly in the range (0, 1].
  - inline double dsfmt\_genrand\_open\_open() generates and returns a double precision pseudorandom number which distributes uniformly in the range (0, 1).
  - void dsfmt\_fill\_array\_close1\_open2() fills the user-specified array with double precision pseudorandom numbers which distribute uniformly in the range [1, 2).
  - void dsfmt\_fill\_array\_close\_open() fills the user-specified array with double precision pseudorandom numbers which distribute uniformly in the range [0, 1).
  - void dsfmt\_fill\_array\_open\_close() fills the user-specified array with double precision pseudorandom numbers which distribute uniformly in the range (0, 1].
  - void dsfmt\_fill\_array\_open\_open() fills the user-specified array with double precision pseudorandom numbers which distribute function uniformly in the range (0, 1).
  - uinit32\_t dsfmt\_genrand\_uint32() generates and returns a 32 bit unsigned integer. This function is useful for generating a small number of integers among a large number of floating point numbers. This function does not have corresponding fill\_array function, because the purpose of fill\_array function is to generate a large number of pseudorandom numbers.

Old names init\_gen\_rand, init\_by\_array, etc. are also defined unless you define DSFMT\_DO\_NOT\_USE\_OLD\_NAMES.

Author:
:   Mutsuo Saito (saito@our-domain) Hiroshima University
:   Makoto Matsumoto (m-mat@our-domain) Hiroshima University

Please change **our-domain** to **math.sci.hiroshima-u.ac.jp**

Date:
:   2012-06-29

Copyright (C) 2007, 2008, 2009 Mutsuo Saito, Makoto Matsumoto and Hiroshima University. Copyright (c) 2011, 2012 Mutsuo Saito, Makoto Matsumoto, Hiroshima University and The University of Tokyo. All rights reserved.

The new BSD License is applied to this software.

```
Copyright (c) 2007, 2008, 2009 Mutsuo Saito, Makoto Matsumoto
and Hiroshima University.
Copyright (c) 2011, 2002 Mutsuo Saito, Makoto Matsumoto, Hiroshima
University and The University of Tokyo.
All rights reserved.

Redistribution and use in source and binary forms, with or without
modification, are permitted provided that the following conditions are
met:

    * Redistributions of source code must retain the above copyright
      notice, this list of conditions and the following disclaimer.
    * Redistributions in binary form must reproduce the above
      copyright notice, this list of conditions and the following
      disclaimer in the documentation and/or other materials provided
      with the distribution.
    * Neither the name of the Hiroshima University nor the names of
      its contributors may be used to endorse or promote products
      derived from this software without specific prior written
      permission.

THIS SOFTWARE IS PROVIDED BY THE COPYRIGHT HOLDERS AND CONTRIBUTORS
"AS IS" AND ANY EXPRESS OR IMPLIED WARRANTIES, INCLUDING, BUT NOT
LIMITED TO, THE IMPLIED WARRANTIES OF MERCHANTABILITY AND FITNESS FOR
A PARTICULAR PURPOSE ARE DISCLAIMED. IN NO EVENT SHALL THE COPYRIGHT
OWNER OR CONTRIBUTORS BE LIABLE FOR ANY DIRECT, INDIRECT, INCIDENTAL,
SPECIAL, EXEMPLARY, OR CONSEQUENTIAL DAMAGES (INCLUDING, BUT NOT
LIMITED TO, PROCUREMENT OF SUBSTITUTE GOODS OR SERVICES; LOSS OF USE,
DATA, OR PROFITS; OR BUSINESS INTERRUPTION) HOWEVER CAUSED AND ON ANY
THEORY OF LIABILITY, WHETHER IN CONTRACT, STRICT LIABILITY, OR TORT
(INCLUDING NEGLIGENCE OR OTHERWISE) ARISING IN ANY WAY OUT OF THE USE
OF THIS SOFTWARE, EVEN IF ADVISED OF THE POSSIBILITY OF SUCH DAMAGE.
```


---

Generated on Fri Jun 29 2012 16:17:32 for dSFMT by  

 1.8.0
