# Supplementary material for: Radiative transfer with reciprocal transactions: Numerical method and its implementation
Source: PLoS One. 2019 Jan 8;14(1):e0210155. doi: 10.1371/journal.pone.0210155 (PMC6324827; doi:10.1371/journal.pone.0210155)
Supplement: S1 Source Code — A link to the latest version: https://bitbucket.org/planetarysystemresearch/r2t2_pub. (ZIP) [file pone.0210155.s001.zip › r2t2_pub/src/dsfmt/dsfmt/html/howto-compile.html]

xml version="1.0" encoding="UTF-8" ?


How to compile dSFMT


## How to compile dSFMT

This document explains how to compile dSFMT for users who
are using UNIX like systems (for example Linux, Free BSD,
cygwin, osx, etc) on terminal. I can't help those who use IDE
(Integrated Development Environment,) please see your IDE's help
to use SIMD feature of your CPU.

### 1. First Step: Compile test programs using Makefile.

#### 1-1. Compile standard C test program.

Check if dSFMT.c and Makefile are in your current directory.
If not, **cd** to the directory where they exist.
Then, type

> ```
> make std
> ```

If it causes an error, try to type

> ```
> cc -DDSFMT_MEXP=19937 -o test-std-M19937 dSFMT.c test.c
> ```

or try to type

> ```
> gcc -DDSFMT_MEXP=19937 -o test-std-M19937 dSFMT.c test.c
> ```

If success, then check the test program. Type

> ```
> ./test-std-M19937 -v
> ```

You will see many random numbers displayed on your screen.
If you want to check these random numbers are correct output,
redirect output to a file and **diff** it with
**dSFMT.19937.out.txt**, like this:

> ```
> ./test-std-M19937 -v > foo.txt
> diff -w foo.txt dSFMT.19937.out.txt
> ```

Silence means they are the same because **diff**
reports the difference of two files.

If you want to know the generation speed of dSFMT, type

> ```
> ./test-std-M19937 -s
> ```

It is very slow. To make it fast, compile it
with **-O3** option. If your compiler is gcc, you
should specify **-fno-strict-aliasing** option
with **-O3**. type

> ```
> gcc -O3 -fno-strict-aliasing -DDSFMT_MEXP=19937 -o test-std-M19937 dSFMT.c test.c
> ./test-std-M19937 -s
> ```

If you are using gcc 4.0, you will get more performance of dSFMT
by giving additional options
**--param max-inline-insns-single=1800**,
**--param inline-unit-growth=500** and
**--param large-function-growth=900**.

#### 1-2. Compile SSE2 test program.

If your CPU supports SSE2 and you can use gcc version 3.4 or later,
you can make test-sse2-M19937. To do this, type

> ```
> make sse2
> ```

or type

> ```
> gcc -O3 -msse2 -fno-strict-aliasing -DHAVE_SSE2=1 -DDSFMT_MEXP=19937 -o test-sse2-M19937 dSFMT.c test.c
> ```

If everything works well,

> ```
> ./test-sse2-M19937 -s
> ```

shows much shorter time than **test-std-M19937 -s**.

#### 1-3. Compile AltiVec test program.

If you are using Macintosh computer with PowerPC G4 or G5, and
your gcc version is later 3.3, you can make test-alti-M19937. To
do this, type

> ```
> make osx-alti
> ```

or type

> ```
> gcc -O3 -faltivec -fno-strict-aliasing -DHAVE_ALTIVEC=1 -DDSFMT_MEXP=19937 -o test-alti-M19937 dSFMT.c test.c
> ```

If everything works well,

> ```
> ./test-alti-M19937 -s
> ```

shows much shorter time than **test-std-M19937 -s**.

#### 1-4. Compile and check output automatically.

To make test program and check output
automatically for all supported SFMT\_MEXPs of dSFMT, type

> ```
> make std-check
> ```

To check test program optimized for SSE2, type

> ```
> make sse2-check
> ```

To check test program optimized for OSX PowerPC AltiVec, type

> ```
> make osx-alti-check
> ```

These commands may take some time.

### 2. Second Step: Use dSFMT pseudorandom number generator with your C program.

#### 2-1. Use sequential call and static link.

Here is a very simple program **sample1.c** which
calculates PI using Monte-Carlo method.

> ```
> #include <stdio.h>
> #include <stdlib.h>
> #include "dSFMT.h"
>
> int main(int argc, char* argv[]) {
>     int i, cnt, seed;
>     double x, y, pi;
>     const int NUM = 10000;
>     dsfmt_t dsfmt;
>
>     if (argc >= 2) {
> 	seed = strtol(argv[1], NULL, 10);
>     } else {
> 	seed = 12345;
>     }
>     cnt = 0;
>     dsfmt_init_gen_rand(&dsfmt, seed);
>     for (i = 0; i < NUM; i++) {
> 	x = dsfmt_genrand_close_open(&dsfmt);
> 	y = dsfmt_genrand_close_open(&dsfmt);
> 	if (x * x + y * y < 1.0) {
> 	    cnt++;
> 	}
>     }
>     pi = (double)cnt / NUM * 4;
>     printf("%f\n", pi);
>     return 0;
> }
> ```

To compile **sample1.c** with dSFMT.c with the period of
2607, type

> ```
> gcc -DDSFMT_MEXP=521 -o sample1 dSFMT.c sample1.c
> ```

If your CPU supports SSE2 and you want to use optimized dSFMT for
SSE2, type

> ```
> gcc -msse2 -DDSFMT_MEXP=521 -DHAVE_SSE2 -o sample1 dSFMT.c sample1.c
> ```

If your Computer is Apple PowerPC G4 or G5 and you want to use
optimized dSFMT for AltiVec, type

> ```
> gcc -faltivec -DDSFMT_MEXP=521 -DHAVE_ALTIVEC -o sample1 dSFMT.c sample1.c
> ```

#### 2-2. Use block call and static link.

Here is **sample2.c** which modifies sample1.c.
The block call **dsfmt\_fill\_array\_close\_open** is
much faster than sequential call, but it needs an aligned
memory. The standard function to get an aligned memory
is **posix\_memalign**, but it isn't usable in every
OS.

> ```
> #include <stdio.h>
> #define _XOPEN_SOURCE 600
> #include <stdlib.h>
> #include "dSFMT.h"
>
> int main(int argc, char* argv[]) {
>     int i, j, cnt, seed;
>     double x, y, pi;
>     const int NUM = 10000;
>     const int R_SIZE = 2 * NUM;
>     int size;
>     double *array;
>     dsfmt_t dsfmt;
>
>     if (argc >= 2) {
> 	seed = strtol(argv[1], NULL, 10);
>     } else {
> 	seed = 12345;
>     }
>     size = dsfmt_get_min_array_size();
>     if (size < R_SIZE) {
> 	size = R_SIZE;
>     }
> #if defined(__APPLE__) || \
>     (defined(__FreeBSD__) && __FreeBSD__ >= 3 && __FreeBSD__ <= 6)
>     printf("malloc used\n");
>     array = malloc(sizeof(double) * size);
>     if (array == NULL) {
> 	printf("can't allocate memory.\n");
> 	return 1;
>     }
> #elif defined(_POSIX_C_SOURCE)
>     printf("posix_memalign used\n");
>     if (posix_memalign((void **)&array, 16, sizeof(double) * size) != 0) {
> 	printf("can't allocate memory.\n");
> 	return 1;
>     }
> #elif defined(__GNUC__) && (__GNUC__ > 3 || (__GNUC__ == 3 && __GNUC_MINOR__ >= 3))
>     printf("memalign used\n");
>     array = memalign(16, sizeof(double) * size);
>     if (array == NULL) {
> 	printf("can't allocate memory.\n");
> 	return 1;
>     }
> #else /* in this case, gcc doesn't suppport SSE2 */
>     array = malloc(sizeof(double) * size);
>     if (array == NULL) {
> 	printf("can't allocate memory.\n");
> 	return 1;
>     }
> #endif
>     cnt = 0;
>     j = 0;
>     dsfmt_init_gen_rand(&dsfmt, seed);
>     dsfmt_fill_array_close_open(&dsfmt, array, size);
>     for (i = 0; i < NUM; i++) {
> 	x = array[j++];
> 	y = array[j++];
> 	if (x * x + y * y < 1.0) {
> 	    cnt++;
> 	}
>     }
>     free(array);
>     pi = (double)cnt / NUM * 4;
>     printf("%f\n", pi);
>     return 0;
> }
> ```

To compile **sample2.c** with dSFMT.c with the period of
22281, type

> ```
> gcc -DDSFMT_MEXP=2203 -o sample2 dSFMT.c sample2.c
> ```

If your CPU supports SSE2 and you want to use optimized dSFMT for
SSE2, type

> ```
> gcc -msse2 -DDSFMT_MEXP=2203 -DHAVE_SSE2 -o sample2 dSFMT.c sample2.c
> ```

If your computer is Apple PowerPC G4 or G5 and you want to use
optimized dSFMT for AltiVec, type

> ```
> gcc -faltivec -DDSFMT_MEXP=2203 -DHAVE_ALTIVEC -o sample2 dSFMT.c sample2.c
> ```

#### 2-3. Initialize dSFMT using dsfmt\_init\_by\_array function.

Here is **sample3.c** which modifies sample1.c.
The 32-bit integer seed can only make 232 kinds of
initial state, to avoid this problem, dSFMT
provides **dsfmt\_init\_by\_array** function. This sample
uses dsfmt\_init\_by\_array function which initialize the internal state
array with an array of 32-bit. The size of an array can be
larger than the internal state array and all elements of the
array are used for initialization, but too large array is
wasteful.

> ```
> #include <stdio.h>
> #include <string.h>
> #include "dSFMT.h"
>
> int main(int argc, char* argv[]) {
>     int i, cnt, seed_cnt;
>     double x, y, pi;
>     const int NUM = 10000;
>     uint32_t seeds[100];
>     dsfmt_t dsfmt;
>
>     if (argc >= 2) {
> 	seed_cnt = 0;
> 	for (i = 0; (i < 100) && (i < strlen(argv[1])); i++) {
> 	    seeds[i] = argv[1][i];
> 	    seed_cnt++;
> 	}
>     } else {
> 	seeds[0] = 12345;
> 	seed_cnt = 1;
>     }
>     cnt = 0;
>     dsfmt_init_by_array(&dsfmt, seeds, seed_cnt);
>     for (i = 0; i < NUM; i++) {
> 	x = dsfmt_genrand_close_open(&dsfmt);
> 	y = dsfmt_genrand_close_open(&dsfmt);
> 	if (x * x + y * y < 1.0) {
> 	    cnt++;
> 	}
>     }
>     pi = (double)cnt / NUM * 4;
>     printf("%f\n", pi);
>     return 0;
> }
> ```

To compile **sample3.c**, type

> ```
> gcc -DDSFMT_MEXP=1279 -o sample3 dSFMT.c sample3.c
> ```

Now, seed can be a string. Like this:

> ```
> ./sample3 your-full-name
> ```
