# Supplementary material for: Radiative transfer with reciprocal transactions: Numerical method and its implementation
Source: PLoS One. 2019 Jan 8;14(1):e0210155. doi: 10.1371/journal.pone.0210155 (PMC6324827; doi:10.1371/journal.pone.0210155)
Supplement: S1 Source Code — A link to the latest version: https://bitbucket.org/planetarysystemresearch/r2t2_pub. (ZIP) [file pone.0210155.s001.zip › r2t2_pub/src/dsfmt/dsfmt/html/globals_func.html]

dSFMT: Globals


|  |
| --- |
| dSFMT  2.2 |

- Main Page
- Data Structures
- Files

- File List
- Globals

- All
- Functions
- Variables
- Typedefs
- Defines

- c
- d
- f
- g
- i
- p

### - c -

- convert\_c0o1()
  : dSFMT.c
- convert\_o0c1()
  : dSFMT.c
- convert\_o0o1()
  : dSFMT.c

### - d -

- do\_recursion()
  : dSFMT-common.h
- dsfmt\_chk\_init\_by\_array()
  : dSFMT.h
  , dSFMT.c
- dsfmt\_chk\_init\_gen\_rand()
  : dSFMT.h
  , dSFMT.c
- dsfmt\_fill\_array\_close1\_open2()
  : dSFMT.h
  , dSFMT.c
- dsfmt\_fill\_array\_close\_open()
  : dSFMT.h
  , dSFMT.c
- dsfmt\_fill\_array\_open\_close()
  : dSFMT.h
  , dSFMT.c
- dsfmt\_fill\_array\_open\_open()
  : dSFMT.h
  , dSFMT.c
- dsfmt\_gen\_rand\_all()
  : dSFMT.h
  , dSFMT.c
- dsfmt\_genrand\_close1\_open2()
  : dSFMT.h
- dsfmt\_genrand\_close\_open()
  : dSFMT.h
- dsfmt\_genrand\_open\_close()
  : dSFMT.h
- dsfmt\_genrand\_open\_open()
  : dSFMT.h
- dsfmt\_genrand\_uint32()
  : dSFMT.h
- dsfmt\_get\_idstring()
  : dSFMT.h
  , dSFMT.c
- dsfmt\_get\_min\_array\_size()
  : dSFMT.h
  , dSFMT.c
- dsfmt\_gv\_fill\_array\_close1\_open2()
  : dSFMT.h
- dsfmt\_gv\_fill\_array\_close\_open()
  : dSFMT.h
- dsfmt\_gv\_fill\_array\_open\_close()
  : dSFMT.h
- dsfmt\_gv\_fill\_array\_open\_open()
  : dSFMT.h
- dsfmt\_gv\_genrand\_close1\_open2()
  : dSFMT.h
- dsfmt\_gv\_genrand\_close\_open()
  : dSFMT.h
- dsfmt\_gv\_genrand\_open\_close()
  : dSFMT.h
- dsfmt\_gv\_genrand\_open\_open()
  : dSFMT.h
- dsfmt\_gv\_genrand\_uint32()
  : dSFMT.h
- dsfmt\_gv\_init\_by\_array()
  : dSFMT.h
- dsfmt\_gv\_init\_gen\_rand()
  : dSFMT.h
- dsfmt\_init\_by\_array()
  : dSFMT.h
- dsfmt\_init\_gen\_rand()
  : dSFMT.h

### - f -

- fill\_array\_close1\_open2()
  : dSFMT.h
- fill\_array\_close\_open()
  : dSFMT.h
- fill\_array\_open\_close()
  : dSFMT.h
- fill\_array\_open\_open()
  : dSFMT.h

### - g -

- gen\_rand\_array\_c0o1()
  : dSFMT.c
- gen\_rand\_array\_c1o2()
  : dSFMT.c
- gen\_rand\_array\_o0c1()
  : dSFMT.c
- gen\_rand\_array\_o0o1()
  : dSFMT.c
- genrand\_close1\_open2()
  : dSFMT.h
- genrand\_close\_open()
  : dSFMT.h
- genrand\_open\_close()
  : dSFMT.h
- genrand\_open\_open()
  : dSFMT.h
- get\_idstring()
  : dSFMT.h
- get\_min\_array\_size()
  : dSFMT.h

### - i -

- idxof()
  : dSFMT.c
- ini\_func1()
  : dSFMT.c
- ini\_func2()
  : dSFMT.c
- init\_by\_array()
  : dSFMT.h
- init\_gen\_rand()
  : dSFMT.h
- initial\_mask()
  : dSFMT.c

### - p -

- period\_certification()
  : dSFMT.c


---

Generated on Fri Jun 29 2012 16:17:32 for dSFMT by  

 1.8.0
