# Supplementary material for: Radiative transfer with reciprocal transactions: Numerical method and its implementation
Source: PLoS One. 2019 Jan 8;14(1):e0210155. doi: 10.1371/journal.pone.0210155 (PMC6324827; doi:10.1371/journal.pone.0210155)
Supplement: S1 Source Code — A link to the latest version: https://bitbucket.org/planetarysystemresearch/r2t2_pub. (ZIP) [file pone.0210155.s001.zip › r2t2_pub/src/dsfmt/dsfmt/html/files.html]

dSFMT: File List


|  |
| --- |
| dSFMT  2.2 |

- Main Page
- Data Structures
- Files

- File List
- Globals

File List

Here is a list of all files with brief descriptions:

|  |  |
| --- | --- |
| dSFMT-common.h [code] | SIMD oriented Fast Mersenne Twister(SFMT) pseudorandom number generator with jump function |
| dSFMT.c | Double precision SIMD-oriented Fast Mersenne Twister (dSFMT) based on IEEE 754 format |
| dSFMT.h [code] | Double precision SIMD oriented Fast Mersenne Twister(dSFMT) pseudorandom number generator based on IEEE 754 format |


---

Generated on Fri Jun 29 2012 16:17:32 for dSFMT by  

 1.8.0
