# Supplementary material for: Radiative transfer with reciprocal transactions: Numerical method and its implementation
Source: PLoS One. 2019 Jan 8;14(1):e0210155. doi: 10.1371/journal.pone.0210155 (PMC6324827; doi:10.1371/journal.pone.0210155)
Supplement: S1 Source Code — A link to the latest version: https://bitbucket.org/planetarysystemresearch/r2t2_pub. (ZIP) [file pone.0210155.s001.zip › r2t2_pub/src/dsfmt/dsfmt/html/d_s_f_m_t_8h_source.html]

dSFMT: dSFMT.h Source File


|  |
| --- |
| dSFMT  2.2 |

- Main Page
- Data Structures
- Files

- File List
- Globals

dSFMT.h

Go to the documentation of this file.

```
00001 #pragma once
00002 
00035 #ifndef DSFMT_H
00036 #define DSFMT_H
00037 #if defined(__cplusplus)
00038 extern "C" {
00039 #endif
00040 
00041 #include <stdio.h>
00042 #include <assert.h>
00043 
00044 #if !defined(DSFMT_MEXP)
00045 #ifdef __GNUC__
00046   #warning "DSFMT_MEXP is not defined. I assume DSFMT_MEXP is 19937."
00047 #endif
00048   #define DSFMT_MEXP 19937
00049 #endif
00050 /*-----------------
00051   BASIC DEFINITIONS
00052   -----------------*/
00053 /* Mersenne Exponent. The period of the sequence
00054  *  is a multiple of 2^DSFMT_MEXP-1.
00055  * #define DSFMT_MEXP 19937 */
00058 #define DSFMT_N ((DSFMT_MEXP - 128) / 104 + 1)
00059 
00061 #define DSFMT_N32 (DSFMT_N * 4)
00062 
00064 #define DSFMT_N64 (DSFMT_N * 2)
00065 
00066 #if !defined(DSFMT_BIG_ENDIAN)
00067 #  if defined(__BYTE_ORDER) && defined(__BIG_ENDIAN)
00068 #    if __BYTE_ORDER == __BIG_ENDIAN
00069 #      define DSFMT_BIG_ENDIAN 1
00070 #    endif
00071 #  elif defined(_BYTE_ORDER) && defined(_BIG_ENDIAN)
00072 #    if _BYTE_ORDER == _BIG_ENDIAN
00073 #      define DSFMT_BIG_ENDIAN 1
00074 #    endif
00075 #  elif defined(__BYTE_ORDER__) && defined(__BIG_ENDIAN__)
00076 #    if __BYTE_ORDER__ == __BIG_ENDIAN__
00077 #      define DSFMT_BIG_ENDIAN 1
00078 #    endif
00079 #  elif defined(BYTE_ORDER) && defined(BIG_ENDIAN)
00080 #    if BYTE_ORDER == BIG_ENDIAN
00081 #      define DSFMT_BIG_ENDIAN 1
00082 #    endif
00083 #  elif defined(__BIG_ENDIAN) || defined(_BIG_ENDIAN) \
00084     || defined(__BIG_ENDIAN__) || defined(BIG_ENDIAN)
00085 #      define DSFMT_BIG_ENDIAN 1
00086 #  endif
00087 #endif
00088 
00089 #if defined(DSFMT_BIG_ENDIAN) && defined(__amd64)
00090 #  undef DSFMT_BIG_ENDIAN
00091 #endif
00092 
00093 #if defined(__STDC_VERSION__) && (__STDC_VERSION__ >= 199901L)
00094 #  include <inttypes.h>
00095 #elif defined(_MSC_VER) || defined(__BORLANDC__)
00096 #  if !defined(DSFMT_UINT32_DEFINED) && !defined(SFMT_UINT32_DEFINED)
00097 typedef unsigned int uint32_t;
00098 typedef unsigned __int64 uint64_t;
00099 #    define UINT64_C(v) (v ## ui64)
00100 #    define DSFMT_UINT32_DEFINED
00101 #    if !defined(inline)
00102 #      define inline __inline
00103 #    endif
00104 #  endif
00105 #else
00106 #  include <inttypes.h>
00107 #  if !defined(inline)
00108 #    if defined(__GNUC__)
00109 #      define inline __inline__
00110 #    else
00111 #      define inline
00112 #    endif
00113 #  endif
00114 #endif
00115 
00116 #ifndef PRIu64
00117 #  if defined(_MSC_VER) || defined(__BORLANDC__)
00118 #    define PRIu64 "I64u"
00119 #    define PRIx64 "I64x"
00120 #  else
00121 #    define PRIu64 "llu"
00122 #    define PRIx64 "llx"
00123 #  endif
00124 #endif
00125 
00126 #ifndef UINT64_C
00127 #  define UINT64_C(v) (v ## ULL)
00128 #endif
00129 
00130 /*------------------------------------------
00131   128-bit SIMD like data type for standard C
00132   ------------------------------------------*/
00133 #if defined(HAVE_ALTIVEC)
00134 #  if !defined(__APPLE__)
00135 #    include <altivec.h>
00136 #  endif
00137 
00138 union W128_T {
00139     vector unsigned int s;
00140     uint64_t u[2];
00141     uint32_t u32[4];
00142     double d[2];
00143 };
00144 
00145 #elif defined(HAVE_SSE2)
00146 #  include <emmintrin.h>
00147 
00149 union W128_T {
00150     __m128i si;
00151     __m128d sd;
00152     uint64_t u[2];
00153     uint32_t u32[4];
00154     double d[2];
00155 };
00156 #else  /* standard C */
00157 
00158 union W128_T {
00159     uint64_t u[2];
00160     uint32_t u32[4];
00161     double d[2];
00162 };
00163 #endif
00164 
00166 typedef union W128_T w128_t;
00167 
00169 struct DSFMT_T {
00170     w128_t status[DSFMT_N + 1];
00171     int idx;
00172 };
00173 typedef struct DSFMT_T dsfmt_t;
00174 
00176 extern dsfmt_t dsfmt_global_data;
00178 extern const int dsfmt_global_mexp;
00179 
00180 void dsfmt_gen_rand_all(dsfmt_t *dsfmt);
00181 void dsfmt_fill_array_open_close(dsfmt_t *dsfmt, double array[], int size);
00182 void dsfmt_fill_array_close_open(dsfmt_t *dsfmt, double array[], int size);
00183 void dsfmt_fill_array_open_open(dsfmt_t *dsfmt, double array[], int size);
00184 void dsfmt_fill_array_close1_open2(dsfmt_t *dsfmt, double array[], int size);
00185 void dsfmt_chk_init_gen_rand(dsfmt_t *dsfmt, uint32_t seed, int mexp);
00186 void dsfmt_chk_init_by_array(dsfmt_t *dsfmt, uint32_t init_key[],
00187                              int key_length, int mexp);
00188 const char *dsfmt_get_idstring(void);
00189 int dsfmt_get_min_array_size(void);
00190 
00191 #if defined(__GNUC__)
00192 #  define DSFMT_PRE_INLINE inline static
00193 #  define DSFMT_PST_INLINE __attribute__((always_inline))
00194 #elif defined(_MSC_VER) && _MSC_VER >= 1200
00195 #  define DSFMT_PRE_INLINE __forceinline static
00196 #  define DSFMT_PST_INLINE
00197 #else
00198 #  define DSFMT_PRE_INLINE inline static
00199 #  define DSFMT_PST_INLINE
00200 #endif
00201 DSFMT_PRE_INLINE uint32_t dsfmt_genrand_uint32(dsfmt_t *dsfmt) DSFMT_PST_INLINE;
00202 DSFMT_PRE_INLINE double dsfmt_genrand_close1_open2(dsfmt_t *dsfmt)
00203     DSFMT_PST_INLINE;
00204 DSFMT_PRE_INLINE double dsfmt_genrand_close_open(dsfmt_t *dsfmt)
00205     DSFMT_PST_INLINE;
00206 DSFMT_PRE_INLINE double dsfmt_genrand_open_close(dsfmt_t *dsfmt)
00207     DSFMT_PST_INLINE;
00208 DSFMT_PRE_INLINE double dsfmt_genrand_open_open(dsfmt_t *dsfmt)
00209     DSFMT_PST_INLINE;
00210 DSFMT_PRE_INLINE uint32_t dsfmt_gv_genrand_uint32(void) DSFMT_PST_INLINE;
00211 DSFMT_PRE_INLINE double dsfmt_gv_genrand_close1_open2(void) DSFMT_PST_INLINE;
00212 DSFMT_PRE_INLINE double dsfmt_gv_genrand_close_open(void) DSFMT_PST_INLINE;
00213 DSFMT_PRE_INLINE double dsfmt_gv_genrand_open_close(void) DSFMT_PST_INLINE;
00214 DSFMT_PRE_INLINE double dsfmt_gv_genrand_open_open(void) DSFMT_PST_INLINE;
00215 DSFMT_PRE_INLINE void dsfmt_gv_fill_array_open_close(double array[], int size)
00216     DSFMT_PST_INLINE;
00217 DSFMT_PRE_INLINE void dsfmt_gv_fill_array_close_open(double array[], int size)
00218     DSFMT_PST_INLINE;
00219 DSFMT_PRE_INLINE void dsfmt_gv_fill_array_open_open(double array[], int size)
00220     DSFMT_PST_INLINE;
00221 DSFMT_PRE_INLINE void dsfmt_gv_fill_array_close1_open2(double array[], int size)
00222     DSFMT_PST_INLINE;
00223 DSFMT_PRE_INLINE void dsfmt_gv_init_gen_rand(uint32_t seed) DSFMT_PST_INLINE;
00224 DSFMT_PRE_INLINE void dsfmt_gv_init_by_array(uint32_t init_key[],
00225                                              int key_length) DSFMT_PST_INLINE;
00226 DSFMT_PRE_INLINE void dsfmt_init_gen_rand(dsfmt_t *dsfmt, uint32_t seed)
00227     DSFMT_PST_INLINE;
00228 DSFMT_PRE_INLINE void dsfmt_init_by_array(dsfmt_t *dsfmt, uint32_t init_key[],
00229                                           int key_length) DSFMT_PST_INLINE;
00230 
00239 inline static uint32_t dsfmt_genrand_uint32(dsfmt_t *dsfmt) {
00240     uint32_t r;
00241     uint64_t *psfmt64 = &dsfmt->status[0].u[0];
00242 
00243     if (dsfmt->idx >= DSFMT_N64) {
00244         dsfmt_gen_rand_all(dsfmt);
00245         dsfmt->idx = 0;
00246     }
00247     r = psfmt64[dsfmt->idx++] & 0xffffffffU;
00248     return r;
00249 }
00250 
00260 inline static double dsfmt_genrand_close1_open2(dsfmt_t *dsfmt) {
00261     double r;
00262     double *psfmt64 = &dsfmt->status[0].d[0];
00263 
00264     if (dsfmt->idx >= DSFMT_N64) {
00265         dsfmt_gen_rand_all(dsfmt);
00266         dsfmt->idx = 0;
00267     }
00268     r = psfmt64[dsfmt->idx++];
00269     return r;
00270 }
00271 
00279 inline static uint32_t dsfmt_gv_genrand_uint32(void) {
00280     return dsfmt_genrand_uint32(&dsfmt_global_data);
00281 }
00282 
00290 inline static double dsfmt_gv_genrand_close1_open2(void) {
00291     return dsfmt_genrand_close1_open2(&dsfmt_global_data);
00292 }
00293 
00302 inline static double dsfmt_genrand_close_open(dsfmt_t *dsfmt) {
00303     return dsfmt_genrand_close1_open2(dsfmt) - 1.0;
00304 }
00305 
00313 inline static double dsfmt_gv_genrand_close_open(void) {
00314     return dsfmt_gv_genrand_close1_open2() - 1.0;
00315 }
00316 
00325 inline static double dsfmt_genrand_open_close(dsfmt_t *dsfmt) {
00326     return 2.0 - dsfmt_genrand_close1_open2(dsfmt);
00327 }
00328 
00336 inline static double dsfmt_gv_genrand_open_close(void) {
00337     return 2.0 - dsfmt_gv_genrand_close1_open2();
00338 }
00339 
00348 inline static double dsfmt_genrand_open_open(dsfmt_t *dsfmt) {
00349     double *dsfmt64 = &dsfmt->status[0].d[0];
00350     union {
00351         double d;
00352         uint64_t u;
00353     } r;
00354 
00355     if (dsfmt->idx >= DSFMT_N64) {
00356         dsfmt_gen_rand_all(dsfmt);
00357         dsfmt->idx = 0;
00358     }
00359     r.d = dsfmt64[dsfmt->idx++];
00360     r.u |= 1;
00361     return r.d - 1.0;
00362 }
00363 
00371 inline static double dsfmt_gv_genrand_open_open(void) {
00372     return dsfmt_genrand_open_open(&dsfmt_global_data);
00373 }
00374 
00386 inline static void dsfmt_gv_fill_array_close1_open2(double array[], int size) {
00387     dsfmt_fill_array_close1_open2(&dsfmt_global_data, array, size);
00388 }
00389 
00402 inline static void dsfmt_gv_fill_array_open_close(double array[], int size) {
00403     dsfmt_fill_array_open_close(&dsfmt_global_data, array, size);
00404 }
00405 
00418 inline static void dsfmt_gv_fill_array_close_open(double array[], int size) {
00419     dsfmt_fill_array_close_open(&dsfmt_global_data, array, size);
00420 }
00421 
00434 inline static void dsfmt_gv_fill_array_open_open(double array[], int size) {
00435     dsfmt_fill_array_open_open(&dsfmt_global_data, array, size);
00436 }
00437 
00444 inline static void dsfmt_init_gen_rand(dsfmt_t *dsfmt, uint32_t seed) {
00445     dsfmt_chk_init_gen_rand(dsfmt, seed, DSFMT_MEXP);
00446 }
00447 
00454 inline static void dsfmt_gv_init_gen_rand(uint32_t seed) {
00455     dsfmt_init_gen_rand(&dsfmt_global_data, seed);
00456 }
00457 
00465 inline static void dsfmt_init_by_array(dsfmt_t *dsfmt, uint32_t init_key[],
00466                                        int key_length) {
00467     dsfmt_chk_init_by_array(dsfmt, init_key, key_length, DSFMT_MEXP);
00468 }
00469 
00478 inline static void dsfmt_gv_init_by_array(uint32_t init_key[], int key_length) {
00479     dsfmt_init_by_array(&dsfmt_global_data, init_key, key_length);
00480 }
00481 
00482 #if !defined(DSFMT_DO_NOT_USE_OLD_NAMES)
00483 DSFMT_PRE_INLINE const char *get_idstring(void) DSFMT_PST_INLINE;
00484 DSFMT_PRE_INLINE int get_min_array_size(void) DSFMT_PST_INLINE;
00485 DSFMT_PRE_INLINE void init_gen_rand(uint32_t seed) DSFMT_PST_INLINE;
00486 DSFMT_PRE_INLINE void init_by_array(uint32_t init_key[], int key_length)
00487     DSFMT_PST_INLINE;
00488 DSFMT_PRE_INLINE double genrand_close1_open2(void) DSFMT_PST_INLINE;
00489 DSFMT_PRE_INLINE double genrand_close_open(void) DSFMT_PST_INLINE;
00490 DSFMT_PRE_INLINE double genrand_open_close(void) DSFMT_PST_INLINE;
00491 DSFMT_PRE_INLINE double genrand_open_open(void) DSFMT_PST_INLINE;
00492 DSFMT_PRE_INLINE void fill_array_open_close(double array[], int size)
00493     DSFMT_PST_INLINE;
00494 DSFMT_PRE_INLINE void fill_array_close_open(double array[], int size)
00495     DSFMT_PST_INLINE;
00496 DSFMT_PRE_INLINE void fill_array_open_open(double array[], int size)
00497     DSFMT_PST_INLINE;
00498 DSFMT_PRE_INLINE void fill_array_close1_open2(double array[], int size)
00499     DSFMT_PST_INLINE;
00500 
00506 inline static const char *get_idstring(void) {
00507     return dsfmt_get_idstring();
00508 }
00509 
00515 inline static int get_min_array_size(void) {
00516     return dsfmt_get_min_array_size();
00517 }
00518 
00524 inline static void init_gen_rand(uint32_t seed) {
00525     dsfmt_gv_init_gen_rand(seed);
00526 }
00527 
00534 inline static void init_by_array(uint32_t init_key[], int key_length) {
00535     dsfmt_gv_init_by_array(init_key, key_length);
00536 }
00537 
00544 inline static double genrand_close1_open2(void) {
00545     return dsfmt_gv_genrand_close1_open2();
00546 }
00547 
00554 inline static double genrand_close_open(void) {
00555     return dsfmt_gv_genrand_close_open();
00556 }
00557 
00564 inline static double genrand_open_close(void) {
00565     return dsfmt_gv_genrand_open_close();
00566 }
00567 
00574 inline static double genrand_open_open(void) {
00575     return dsfmt_gv_genrand_open_open();
00576 }
00577 
00587 inline static void fill_array_open_close(double array[], int size) {
00588     dsfmt_gv_fill_array_open_close(array, size);
00589 }
00590 
00600 inline static void fill_array_close_open(double array[], int size) {
00601     dsfmt_gv_fill_array_close_open(array, size);
00602 }
00603 
00613 inline static void fill_array_open_open(double array[], int size) {
00614     dsfmt_gv_fill_array_open_open(array, size);
00615 }
00616 
00625 inline static void fill_array_close1_open2(double array[], int size) {
00626     dsfmt_gv_fill_array_close1_open2(array, size);
00627 }
00628 #endif /* DSFMT_DO_NOT_USE_OLD_NAMES */
00629 
00630 #if defined(__cplusplus)
00631 }
00632 #endif
00633 
00634 #endif /* DSFMT_H */
```


---

Generated on Fri Jun 29 2012 16:17:32 for dSFMT by  

 1.8.0
