# Supplementary material for: Radiative transfer with reciprocal transactions: Numerical method and its implementation
Source: PLoS One. 2019 Jan 8;14(1):e0210155. doi: 10.1371/journal.pone.0210155 (PMC6324827; doi:10.1371/journal.pone.0210155)
Supplement: S1 Source Code — A link to the latest version: https://bitbucket.org/planetarysystemresearch/r2t2_pub. (ZIP) [file pone.0210155.s001.zip › r2t2_pub/src/dsfmt/dsfmt/html/d_s_f_m_t_8h.html]

dSFMT: dSFMT.h File Reference


|  |
| --- |
| dSFMT  2.2 |

- Main Page
- Data Structures
- Files

- File List
- Globals

Data Structures |
Defines |
Typedefs |
Functions |
Variables

dSFMT.h File Reference

double precision SIMD oriented Fast Mersenne Twister(dSFMT) pseudorandom number generator based on IEEE 754 format.
More...

`#include <stdio.h>`  
`#include <assert.h>`  
`#include <inttypes.h>`

Go to the source code of this file.

|  |  |
| --- | --- |
| Data Structures | |
| union | W128\_T |
|  | 128-bit data structure More... |
| struct | DSFMT\_T |
|  | the 128-bit internal state array More... |
| Defines | |
| #define | DSFMT\_MEXP   19937 |
| #define | DSFMT\_N   ((DSFMT\_MEXP - 128) / 104 + 1) |
|  | DSFMT generator has an internal state array of 128-bit integers, and N is its size. |
| #define | DSFMT\_N32   (DSFMT\_N \* 4) |
|  | N32 is the size of internal state array when regarded as an array of 32-bit integers. |
| #define | DSFMT\_N64   (DSFMT\_N \* 2) |
|  | N64 is the size of internal state array when regarded as an array of 64-bit integers. |
| #define | inline |
| #define | PRIu64   "llu" |
| #define | PRIx64   "llx" |
| #define | UINT64\_C(v)   (v ## ULL) |
| #define | DSFMT\_PRE\_INLINE   inline static |
| #define | DSFMT\_PST\_INLINE |
| Typedefs | |
| typedef union W128\_T | w128\_t |
|  | 128-bit data type |
| typedef struct DSFMT\_T | dsfmt\_t |
| Functions | |
| void | dsfmt\_gen\_rand\_all (dsfmt\_t \*dsfmt) |
|  | This function fills the internal state array with double precision floating point pseudorandom numbers of the IEEE 754 format. |
| void | dsfmt\_fill\_array\_open\_close (dsfmt\_t \*dsfmt, double array[], int size) |
|  | This function generates double precision floating point pseudorandom numbers which distribute in the range (0, 1] to the specified array[] by one call. |
| void | dsfmt\_fill\_array\_close\_open (dsfmt\_t \*dsfmt, double array[], int size) |
|  | This function generates double precision floating point pseudorandom numbers which distribute in the range [0, 1) to the specified array[] by one call. |
| void | dsfmt\_fill\_array\_open\_open (dsfmt\_t \*dsfmt, double array[], int size) |
|  | This function generates double precision floating point pseudorandom numbers which distribute in the range (0, 1) to the specified array[] by one call. |
| void | dsfmt\_fill\_array\_close1\_open2 (dsfmt\_t \*dsfmt, double array[], int size) |
|  | This function generates double precision floating point pseudorandom numbers which distribute in the range [1, 2) to the specified array[] by one call. |
| void | dsfmt\_chk\_init\_gen\_rand (dsfmt\_t \*dsfmt, uint32\_t seed, int mexp) |
|  | This function initializes the internal state array with a 32-bit integer seed. |
| void | dsfmt\_chk\_init\_by\_array (dsfmt\_t \*dsfmt, uint32\_t init\_key[], int key\_length, int mexp) |
|  | This function initializes the internal state array, with an array of 32-bit integers used as the seeds. |
| const char \* | dsfmt\_get\_idstring (void) |
|  | This function returns the identification string. |
| int | dsfmt\_get\_min\_array\_size (void) |
|  | This function returns the minimum size of array used for **fill\_array** functions. |
| DSFMT\_PRE\_INLINE uint32\_t | dsfmt\_genrand\_uint32 (dsfmt\_t \*dsfmt) |
|  | This function generates and returns unsigned 32-bit integer. |
| DSFMT\_PRE\_INLINE double | dsfmt\_genrand\_close1\_open2 (dsfmt\_t \*dsfmt) |
|  | This function generates and returns double precision pseudorandom number which distributes uniformly in the range [1, 2). |
| DSFMT\_PRE\_INLINE double | dsfmt\_genrand\_close\_open (dsfmt\_t \*dsfmt) |
|  | This function generates and returns double precision pseudorandom number which distributes uniformly in the range [0, 1). |
| DSFMT\_PRE\_INLINE double | dsfmt\_genrand\_open\_close (dsfmt\_t \*dsfmt) |
|  | This function generates and returns double precision pseudorandom number which distributes uniformly in the range (0, 1]. |
| DSFMT\_PRE\_INLINE double | dsfmt\_genrand\_open\_open (dsfmt\_t \*dsfmt) |
|  | This function generates and returns double precision pseudorandom number which distributes uniformly in the range (0, 1). |
| DSFMT\_PRE\_INLINE uint32\_t | dsfmt\_gv\_genrand\_uint32 (void) |
|  | This function generates and returns unsigned 32-bit integer. |
| DSFMT\_PRE\_INLINE double | dsfmt\_gv\_genrand\_close1\_open2 (void) |
|  | This function generates and returns double precision pseudorandom number which distributes uniformly in the range [1, 2). |
| DSFMT\_PRE\_INLINE double | dsfmt\_gv\_genrand\_close\_open (void) |
|  | This function generates and returns double precision pseudorandom number which distributes uniformly in the range [0, 1). |
| DSFMT\_PRE\_INLINE double | dsfmt\_gv\_genrand\_open\_close (void) |
|  | This function generates and returns double precision pseudorandom number which distributes uniformly in the range (0, 1]. |
| DSFMT\_PRE\_INLINE double | dsfmt\_gv\_genrand\_open\_open (void) |
|  | This function generates and returns double precision pseudorandom number which distributes uniformly in the range (0, 1). |
| DSFMT\_PRE\_INLINE void | dsfmt\_gv\_fill\_array\_open\_close (double array[], int size) |
|  | This function generates double precision floating point pseudorandom numbers which distribute in the range (0, 1] to the specified array[] by one call. |
| DSFMT\_PRE\_INLINE void | dsfmt\_gv\_fill\_array\_close\_open (double array[], int size) |
|  | This function generates double precision floating point pseudorandom numbers which distribute in the range [0, 1) to the specified array[] by one call. |
| DSFMT\_PRE\_INLINE void | dsfmt\_gv\_fill\_array\_open\_open (double array[], int size) |
|  | This function generates double precision floating point pseudorandom numbers which distribute in the range (0, 1) to the specified array[] by one call. |
| DSFMT\_PRE\_INLINE void | dsfmt\_gv\_fill\_array\_close1\_open2 (double array[], int size) |
|  | This function generates double precision floating point pseudorandom numbers which distribute in the range [1, 2) to the specified array[] by one call. |
| DSFMT\_PRE\_INLINE void | dsfmt\_gv\_init\_gen\_rand (uint32\_t seed) |
|  | This function initializes the internal state array with a 32-bit integer seed. |
| DSFMT\_PRE\_INLINE void | dsfmt\_gv\_init\_by\_array (uint32\_t init\_key[], int key\_length) |
|  | This function initializes the internal state array, with an array of 32-bit integers used as the seeds. |
| DSFMT\_PRE\_INLINE void | dsfmt\_init\_gen\_rand (dsfmt\_t \*dsfmt, uint32\_t seed) |
|  | This function initializes the internal state array with a 32-bit integer seed. |
| DSFMT\_PRE\_INLINE void | dsfmt\_init\_by\_array (dsfmt\_t \*dsfmt, uint32\_t init\_key[], int key\_length) |
|  | This function initializes the internal state array, with an array of 32-bit integers used as the seeds. |
| DSFMT\_PRE\_INLINE const char \* | get\_idstring (void) |
|  | This function is just the same as dsfmt\_get\_idstring(). |
| DSFMT\_PRE\_INLINE int | get\_min\_array\_size (void) |
|  | This function is just the same as dsfmt\_get\_min\_array\_size(). |
| DSFMT\_PRE\_INLINE void | init\_gen\_rand (uint32\_t seed) |
|  | This function is just the same as dsfmt\_gv\_init\_gen\_rand(). |
| DSFMT\_PRE\_INLINE void | init\_by\_array (uint32\_t init\_key[], int key\_length) |
|  | This function is just the same as dsfmt\_gv\_init\_by\_array(). |
| DSFMT\_PRE\_INLINE double | genrand\_close1\_open2 (void) |
|  | This function is just the same as dsfmt\_gv\_genrand\_close1\_open2(). |
| DSFMT\_PRE\_INLINE double | genrand\_close\_open (void) |
|  | This function is just the same as dsfmt\_gv\_genrand\_close\_open(). |
| DSFMT\_PRE\_INLINE double | genrand\_open\_close (void) |
|  | This function is just the same as dsfmt\_gv\_genrand\_open\_close(). |
| DSFMT\_PRE\_INLINE double | genrand\_open\_open (void) |
|  | This function is just the same as dsfmt\_gv\_genrand\_open\_open(). |
| DSFMT\_PRE\_INLINE void | fill\_array\_open\_close (double array[], int size) |
|  | This function is juset the same as dsfmt\_gv\_fill\_array\_open\_close(). |
| DSFMT\_PRE\_INLINE void | fill\_array\_close\_open (double array[], int size) |
|  | This function is juset the same as dsfmt\_gv\_fill\_array\_close\_open(). |
| DSFMT\_PRE\_INLINE void | fill\_array\_open\_open (double array[], int size) |
|  | This function is juset the same as dsfmt\_gv\_fill\_array\_open\_open(). |
| DSFMT\_PRE\_INLINE void | fill\_array\_close1\_open2 (double array[], int size) |
|  | This function is juset the same as dsfmt\_gv\_fill\_array\_close1\_open2(). |
| Variables | |
| dsfmt\_t | dsfmt\_global\_data |
|  | dsfmt internal state vector |
| const int | dsfmt\_global\_mexp |
|  | dsfmt mexp for check |

---

## Detailed Description

double precision SIMD oriented Fast Mersenne Twister(dSFMT) pseudorandom number generator based on IEEE 754 format.

Author:
:   Mutsuo Saito (Hiroshima University)
:   Makoto Matsumoto (Hiroshima University)

Copyright (C) 2007, 2008 Mutsuo Saito, Makoto Matsumoto and Hiroshima University. All rights reserved. Copyright (C) 2012 Mutsuo Saito, Makoto Matsumoto, Hiroshima University and The University of Tokyo. All rights reserved.

The new BSD License is applied to this software. see LICENSE.txt

Note:
:   We assume that your system has inttypes.h. If your system doesn't have inttypes.h, you have to typedef uint32\_t and uint64\_t, and you have to define PRIu64 and PRIx64 in this file as follows:

    ```
     typedef unsigned int uint32_t
     typedef unsigned long long uint64_t
     #define PRIu64 "llu"
     #define PRIx64 "llx"
    ```

    uint32\_t must be exactly 32-bit unsigned integer type (no more, no less), and uint64\_t must be exactly 64-bit unsigned integer type. PRIu64 and PRIx64 are used for printf function to print 64-bit unsigned int and 64-bit unsigned int in hexadecimal format.

---

## Define Documentation

|  |
| --- |
| #define DSFMT\_MEXP   19937 |

Referenced by dsfmt\_init\_by\_array(), and dsfmt\_init\_gen\_rand().

|  |
| --- |
| #define DSFMT\_N   ((DSFMT\_MEXP - 128) / 104 + 1) |

DSFMT generator has an internal state array of 128-bit integers, and N is its size.

Referenced by dsfmt\_chk\_init\_by\_array(), dsfmt\_chk\_init\_gen\_rand(), dsfmt\_gen\_rand\_all(), gen\_rand\_array\_c0o1(), gen\_rand\_array\_c1o2(), gen\_rand\_array\_o0c1(), gen\_rand\_array\_o0o1(), initial\_mask(), and period\_certification().

|  |
| --- |
| #define DSFMT\_N32   (DSFMT\_N \* 4) |

N32 is the size of internal state array when regarded as an array of 32-bit integers.

|  |
| --- |
| #define DSFMT\_N64   (DSFMT\_N \* 2) |

N64 is the size of internal state array when regarded as an array of 64-bit integers.

Referenced by dsfmt\_chk\_init\_by\_array(), dsfmt\_chk\_init\_gen\_rand(), dsfmt\_fill\_array\_close1\_open2(), dsfmt\_fill\_array\_close\_open(), dsfmt\_fill\_array\_open\_close(), dsfmt\_fill\_array\_open\_open(), dsfmt\_genrand\_close1\_open2(), dsfmt\_genrand\_open\_open(), dsfmt\_genrand\_uint32(), and dsfmt\_get\_min\_array\_size().

|  |
| --- |
| #define DSFMT\_PRE\_INLINE   inline static |

|  |
| --- |
| #define DSFMT\_PST\_INLINE |

|  |
| --- |
| #define inline |

|  |
| --- |
| #define PRIu64   "llu" |

|  |
| --- |
| #define PRIx64   "llx" |

|  |  |  |  |  |  |
| --- | --- | --- | --- | --- | --- |
| #define UINT64\_C | ( |  | v | ) | (v ## ULL) |

---

## Typedef Documentation

|  |
| --- |
| typedef struct DSFMT\_T dsfmt\_t |

|  |
| --- |
| typedef union W128\_T w128\_t |

128-bit data type

---

## Function Documentation

|  |  |  |  |
| --- | --- | --- | --- |
| void dsfmt\_chk\_init\_by\_array | ( | dsfmt\_t \* | *dsfmt*, |
|  |  | uint32\_t | *init\_key*[], |
|  |  | int | *key\_length*, |
|  |  | int | *mexp* |
|  | ) |  |  |

This function initializes the internal state array, with an array of 32-bit integers used as the seeds.

**Parameters:**
:   |  |  |
    | --- | --- |
    | dsfmt | dsfmt state vector. |
    | init\_key | the array of 32-bit integers, used as a seed. |
    | key\_length | the length of init\_key. |
    | mexp | caller's mersenne expornent |

References DSFMT\_N, DSFMT\_N64, DSFMT\_T::idx, idxof(), ini\_func1(), ini\_func2(), initial\_mask(), period\_certification(), DSFMT\_T::status, and W128\_T::u32.

Referenced by dsfmt\_init\_by\_array().

|  |  |  |  |
| --- | --- | --- | --- |
| void dsfmt\_chk\_init\_gen\_rand | ( | dsfmt\_t \* | *dsfmt*, |
|  |  | uint32\_t | *seed*, |
|  |  | int | *mexp* |
|  | ) |  |  |

This function initializes the internal state array with a 32-bit integer seed.

**Parameters:**
:   |  |  |
    | --- | --- |
    | dsfmt | dsfmt state vector. |
    | seed | a 32-bit integer used as the seed. |
    | mexp | caller's mersenne expornent |

References DSFMT\_N, DSFMT\_N64, DSFMT\_T::idx, idxof(), initial\_mask(), period\_certification(), DSFMT\_T::status, and W128\_T::u32.

Referenced by dsfmt\_init\_gen\_rand().

|  |  |  |  |
| --- | --- | --- | --- |
| void dsfmt\_fill\_array\_close1\_open2 | ( | dsfmt\_t \* | *dsfmt*, |
|  |  | double | *array*[], |
|  |  | int | *size* |
|  | ) |  |  |

This function generates double precision floating point pseudorandom numbers which distribute in the range [1, 2) to the specified array[] by one call.

The number of pseudorandom numbers is specified by the argument **size**, which must be at least (SFMT\_MEXP / 128) \* 2 and a multiple of two. The function get\_min\_array\_size() returns this minimum size. The generation by this function is much faster than the following fill\_array\_xxx functions.

For initialization, init\_gen\_rand() or init\_by\_array() must be called before the first call of this function. This function can not be used after calling genrand\_xxx functions, without initialization.

**Parameters:**
:   |  |  |
    | --- | --- |
    | dsfmt | dsfmt state vector. |
    | array | an array where pseudorandom numbers are filled by this function. The pointer to the array must be "aligned" (namely, must be a multiple of 16) in the SIMD version, since it refers to the address of a 128-bit integer. In the standard C version, the pointer is arbitrary. |
    | size | the number of 64-bit pseudorandom integers to be generated. size must be a multiple of 2, and greater than or equal to (SFMT\_MEXP / 128) \* 2. |

Note:
:   **memalign** or **posix\_memalign** is available to get aligned memory. Mac OSX doesn't have these functions, but **malloc** of OSX returns the pointer to the aligned memory block.

References DSFMT\_N64, and gen\_rand\_array\_c1o2().

Referenced by dsfmt\_gv\_fill\_array\_close1\_open2().

|  |  |  |  |
| --- | --- | --- | --- |
| void dsfmt\_fill\_array\_close\_open | ( | dsfmt\_t \* | *dsfmt*, |
|  |  | double | *array*[], |
|  |  | int | *size* |
|  | ) |  |  |

This function generates double precision floating point pseudorandom numbers which distribute in the range [0, 1) to the specified array[] by one call.

This function is the same as fill\_array\_close1\_open2() except the distribution range.

**Parameters:**
:   |  |  |
    | --- | --- |
    | array | an array where pseudorandom numbers are filled by this function. |
    | dsfmt | dsfmt state vector. |
    | size | the number of pseudorandom numbers to be generated. see also |

See also:
:   fill\_array\_close1\_open2()

References DSFMT\_N64, and gen\_rand\_array\_c0o1().

Referenced by dsfmt\_gv\_fill\_array\_close\_open().

|  |  |  |  |
| --- | --- | --- | --- |
| void dsfmt\_fill\_array\_open\_close | ( | dsfmt\_t \* | *dsfmt*, |
|  |  | double | *array*[], |
|  |  | int | *size* |
|  | ) |  |  |

This function generates double precision floating point pseudorandom numbers which distribute in the range (0, 1] to the specified array[] by one call.

This function is the same as fill\_array\_close1\_open2() except the distribution range.

**Parameters:**
:   |  |  |
    | --- | --- |
    | dsfmt | dsfmt state vector. |
    | array | an array where pseudorandom numbers are filled by this function. |
    | size | the number of pseudorandom numbers to be generated. see also |

See also:
:   fill\_array\_close1\_open2()

References DSFMT\_N64, and gen\_rand\_array\_o0c1().

Referenced by dsfmt\_gv\_fill\_array\_open\_close().

|  |  |  |  |
| --- | --- | --- | --- |
| void dsfmt\_fill\_array\_open\_open | ( | dsfmt\_t \* | *dsfmt*, |
|  |  | double | *array*[], |
|  |  | int | *size* |
|  | ) |  |  |

This function generates double precision floating point pseudorandom numbers which distribute in the range (0, 1) to the specified array[] by one call.

This function is the same as fill\_array\_close1\_open2() except the distribution range.

**Parameters:**
:   |  |  |
    | --- | --- |
    | dsfmt | dsfmt state vector. |
    | array | an array where pseudorandom numbers are filled by this function. |
    | size | the number of pseudorandom numbers to be generated. see also |

See also:
:   fill\_array\_close1\_open2()

References DSFMT\_N64, and gen\_rand\_array\_o0o1().

Referenced by dsfmt\_gv\_fill\_array\_open\_open().

|  |  |  |  |  |  |
| --- | --- | --- | --- | --- | --- |
| void dsfmt\_gen\_rand\_all | ( | dsfmt\_t \* | *dsfmt* | ) |  |

This function fills the internal state array with double precision floating point pseudorandom numbers of the IEEE 754 format.

**Parameters:**
:   |  |  |
    | --- | --- |
    | dsfmt | dsfmt state vector. |

References do\_recursion(), DSFMT\_N, and DSFMT\_T::status.

Referenced by dsfmt\_genrand\_close1\_open2(), dsfmt\_genrand\_open\_open(), and dsfmt\_genrand\_uint32().

|  |  |  |  |  |  |
| --- | --- | --- | --- | --- | --- |
| static double dsfmt\_genrand\_close1\_open2 | ( | dsfmt\_t \* | *dsfmt* | ) | `[inline]` |

This function generates and returns double precision pseudorandom number which distributes uniformly in the range [1, 2).

This is the primitive and faster than generating numbers in other ranges. dsfmt\_init\_gen\_rand() or dsfmt\_init\_by\_array() must be called before this function.

**Parameters:**
:   |  |  |
    | --- | --- |
    | dsfmt | dsfmt internal state date |

Returns:
:   double precision floating point pseudorandom number

References W128\_T::d, dsfmt\_gen\_rand\_all(), DSFMT\_N64, DSFMT\_T::idx, and DSFMT\_T::status.

Referenced by dsfmt\_genrand\_close\_open(), dsfmt\_genrand\_open\_close(), and dsfmt\_gv\_genrand\_close1\_open2().

|  |  |  |  |  |  |
| --- | --- | --- | --- | --- | --- |
| static double dsfmt\_genrand\_close\_open | ( | dsfmt\_t \* | *dsfmt* | ) | `[inline]` |

This function generates and returns double precision pseudorandom number which distributes uniformly in the range [0, 1).

dsfmt\_init\_gen\_rand() or dsfmt\_init\_by\_array() must be called before this function.

**Parameters:**
:   |  |  |
    | --- | --- |
    | dsfmt | dsfmt internal state date |

Returns:
:   double precision floating point pseudorandom number

References dsfmt\_genrand\_close1\_open2().

|  |  |  |  |  |  |
| --- | --- | --- | --- | --- | --- |
| static double dsfmt\_genrand\_open\_close | ( | dsfmt\_t \* | *dsfmt* | ) | `[inline]` |

This function generates and returns double precision pseudorandom number which distributes uniformly in the range (0, 1].

dsfmt\_init\_gen\_rand() or dsfmt\_init\_by\_array() must be called before this function.

**Parameters:**
:   |  |  |
    | --- | --- |
    | dsfmt | dsfmt internal state date |

Returns:
:   double precision floating point pseudorandom number

References dsfmt\_genrand\_close1\_open2().

|  |  |  |  |  |  |
| --- | --- | --- | --- | --- | --- |
| static double dsfmt\_genrand\_open\_open | ( | dsfmt\_t \* | *dsfmt* | ) | `[inline]` |

This function generates and returns double precision pseudorandom number which distributes uniformly in the range (0, 1).

dsfmt\_init\_gen\_rand() or dsfmt\_init\_by\_array() must be called before this function.

**Parameters:**
:   |  |  |
    | --- | --- |
    | dsfmt | dsfmt internal state date |

Returns:
:   double precision floating point pseudorandom number

References W128\_T::d, dsfmt\_gen\_rand\_all(), DSFMT\_N64, DSFMT\_T::idx, and DSFMT\_T::status.

Referenced by dsfmt\_gv\_genrand\_open\_open().

|  |  |  |  |  |  |
| --- | --- | --- | --- | --- | --- |
| static uint32\_t dsfmt\_genrand\_uint32 | ( | dsfmt\_t \* | *dsfmt* | ) | `[inline]` |

This function generates and returns unsigned 32-bit integer.

This is slower than SFMT, only for convenience usage. dsfmt\_init\_gen\_rand() or dsfmt\_init\_by\_array() must be called before this function.

**Parameters:**
:   |  |  |
    | --- | --- |
    | dsfmt | dsfmt internal state date |

Returns:
:   double precision floating point pseudorandom number

References dsfmt\_gen\_rand\_all(), and DSFMT\_N64.

Referenced by dsfmt\_gv\_genrand\_uint32().

|  |  |  |  |  |  |
| --- | --- | --- | --- | --- | --- |
| const char\* dsfmt\_get\_idstring | ( | void |  | ) |  |

This function returns the identification string.

The string shows the Mersenne exponent, and all parameters of this generator.

Returns:
:   id string.

Referenced by get\_idstring().

|  |  |  |  |  |  |
| --- | --- | --- | --- | --- | --- |
| int dsfmt\_get\_min\_array\_size | ( | void |  | ) |  |

This function returns the minimum size of array used for **fill\_array** functions.

Returns:
:   minimum size of array used for fill\_array functions.

References DSFMT\_N64.

Referenced by get\_min\_array\_size().

|  |  |  |  |
| --- | --- | --- | --- |
| static void dsfmt\_gv\_fill\_array\_close1\_open2 | ( | double | *array*[], |
|  |  | int | *size* |
|  | ) |  | `[inline]` |

This function generates double precision floating point pseudorandom numbers which distribute in the range [1, 2) to the specified array[] by one call.

This function is the same as dsfmt\_fill\_array\_close1\_open2() except that this function uses **global** variables.

**Parameters:**
:   |  |  |
    | --- | --- |
    | array | an array where pseudorandom numbers are filled by this function. |
    | size | the number of pseudorandom numbers to be generated. see also |

See also:
:   dsfmt\_fill\_array\_close1\_open2()

References dsfmt\_fill\_array\_close1\_open2().

Referenced by fill\_array\_close1\_open2().

|  |  |  |  |
| --- | --- | --- | --- |
| static void dsfmt\_gv\_fill\_array\_close\_open | ( | double | *array*[], |
|  |  | int | *size* |
|  | ) |  | `[inline]` |

This function generates double precision floating point pseudorandom numbers which distribute in the range [0, 1) to the specified array[] by one call.

This function is the same as dsfmt\_gv\_fill\_array\_close1\_open2() except the distribution range. This function uses **global** variables.

**Parameters:**
:   |  |  |
    | --- | --- |
    | array | an array where pseudorandom numbers are filled by this function. |
    | size | the number of pseudorandom numbers to be generated. see also |

See also:
:   dsfmt\_fill\_array\_close1\_open2()
:   dsfmt\_gv\_fill\_array\_close1\_open2()

References dsfmt\_fill\_array\_close\_open().

Referenced by fill\_array\_close\_open().

|  |  |  |  |
| --- | --- | --- | --- |
| static void dsfmt\_gv\_fill\_array\_open\_close | ( | double | *array*[], |
|  |  | int | *size* |
|  | ) |  | `[inline]` |

This function generates double precision floating point pseudorandom numbers which distribute in the range (0, 1] to the specified array[] by one call.

This function is the same as dsfmt\_gv\_fill\_array\_close1\_open2() except the distribution range. This function uses **global** variables.

**Parameters:**
:   |  |  |
    | --- | --- |
    | array | an array where pseudorandom numbers are filled by this function. |
    | size | the number of pseudorandom numbers to be generated. see also |

See also:
:   dsfmt\_fill\_array\_close1\_open2() and
:   dsfmt\_gv\_fill\_array\_close1\_open2()

References dsfmt\_fill\_array\_open\_close().

Referenced by fill\_array\_open\_close().

|  |  |  |  |
| --- | --- | --- | --- |
| static void dsfmt\_gv\_fill\_array\_open\_open | ( | double | *array*[], |
|  |  | int | *size* |
|  | ) |  | `[inline]` |

This function generates double precision floating point pseudorandom numbers which distribute in the range (0, 1) to the specified array[] by one call.

This function is the same as dsfmt\_gv\_fill\_array\_close1\_open2() except the distribution range. This function uses **global** variables.

**Parameters:**
:   |  |  |
    | --- | --- |
    | array | an array where pseudorandom numbers are filled by this function. |
    | size | the number of pseudorandom numbers to be generated. see also |

See also:
:   dsfmt\_fill\_array\_close1\_open2()
:   dsfmt\_gv\_fill\_array\_close1\_open2()

References dsfmt\_fill\_array\_open\_open().

Referenced by fill\_array\_open\_open().

|  |  |  |  |  |  |
| --- | --- | --- | --- | --- | --- |
| static double dsfmt\_gv\_genrand\_close1\_open2 | ( | void |  | ) | `[inline]` |

This function generates and returns double precision pseudorandom number which distributes uniformly in the range [1, 2).

dsfmt\_gv\_init\_gen\_rand() or dsfmt\_gv\_init\_by\_array() must be called before this function. This function uses **global** variables.

Returns:
:   double precision floating point pseudorandom number

References dsfmt\_genrand\_close1\_open2().

Referenced by dsfmt\_gv\_genrand\_close\_open(), dsfmt\_gv\_genrand\_open\_close(), and genrand\_close1\_open2().

|  |  |  |  |  |  |
| --- | --- | --- | --- | --- | --- |
| static double dsfmt\_gv\_genrand\_close\_open | ( | void |  | ) | `[inline]` |

This function generates and returns double precision pseudorandom number which distributes uniformly in the range [0, 1).

dsfmt\_gv\_init\_gen\_rand() or dsfmt\_gv\_init\_by\_array() must be called before this function. This function uses **global** variables.

Returns:
:   double precision floating point pseudorandom number

References dsfmt\_gv\_genrand\_close1\_open2().

Referenced by genrand\_close\_open().

|  |  |  |  |  |  |
| --- | --- | --- | --- | --- | --- |
| static double dsfmt\_gv\_genrand\_open\_close | ( | void |  | ) | `[inline]` |

This function generates and returns double precision pseudorandom number which distributes uniformly in the range (0, 1].

dsfmt\_gv\_init\_gen\_rand() or dsfmt\_gv\_init\_by\_array() must be called before this function. This function uses **global** variables.

Returns:
:   double precision floating point pseudorandom number

References dsfmt\_gv\_genrand\_close1\_open2().

Referenced by genrand\_open\_close().

|  |  |  |  |  |  |
| --- | --- | --- | --- | --- | --- |
| static double dsfmt\_gv\_genrand\_open\_open | ( | void |  | ) | `[inline]` |

This function generates and returns double precision pseudorandom number which distributes uniformly in the range (0, 1).

dsfmt\_gv\_init\_gen\_rand() or dsfmt\_gv\_init\_by\_array() must be called before this function. This function uses **global** variables.

Returns:
:   double precision floating point pseudorandom number

References dsfmt\_genrand\_open\_open().

Referenced by genrand\_open\_open().

|  |  |  |  |  |  |
| --- | --- | --- | --- | --- | --- |
| static uint32\_t dsfmt\_gv\_genrand\_uint32 | ( | void |  | ) | `[inline]` |

This function generates and returns unsigned 32-bit integer.

This is slower than SFMT, only for convenience usage. dsfmt\_gv\_init\_gen\_rand() or dsfmt\_gv\_init\_by\_array() must be called before this function. This function uses **global** variables.

Returns:
:   double precision floating point pseudorandom number

References dsfmt\_genrand\_uint32().

|  |  |  |  |
| --- | --- | --- | --- |
| static void dsfmt\_gv\_init\_by\_array | ( | uint32\_t | *init\_key*[], |
|  |  | int | *key\_length* |
|  | ) |  | `[inline]` |

This function initializes the internal state array, with an array of 32-bit integers used as the seeds.

This function uses **global** variables.

**Parameters:**
:   |  |  |
    | --- | --- |
    | init\_key | the array of 32-bit integers, used as a seed. |
    | key\_length | the length of init\_key. see also |

See also:
:   dsfmt\_init\_by\_array()

References dsfmt\_init\_by\_array().

Referenced by init\_by\_array().

|  |  |  |  |  |  |
| --- | --- | --- | --- | --- | --- |
| static void dsfmt\_gv\_init\_gen\_rand | ( | uint32\_t | *seed* | ) | `[inline]` |

This function initializes the internal state array with a 32-bit integer seed.

This function uses **global** variables.

**Parameters:**
:   |  |  |
    | --- | --- |
    | seed | a 32-bit integer used as the seed. see also |

See also:
:   dsfmt\_init\_gen\_rand()

References dsfmt\_init\_gen\_rand().

Referenced by init\_gen\_rand().

|  |  |  |  |
| --- | --- | --- | --- |
| static void dsfmt\_init\_by\_array | ( | dsfmt\_t \* | *dsfmt*, |
|  |  | uint32\_t | *init\_key*[], |
|  |  | int | *key\_length* |
|  | ) |  | `[inline]` |

This function initializes the internal state array, with an array of 32-bit integers used as the seeds.

**Parameters:**
:   |  |  |
    | --- | --- |
    | dsfmt | dsfmt state vector |
    | init\_key | the array of 32-bit integers, used as a seed. |
    | key\_length | the length of init\_key. |

References dsfmt\_chk\_init\_by\_array(), and DSFMT\_MEXP.

Referenced by dsfmt\_gv\_init\_by\_array().

|  |  |  |  |
| --- | --- | --- | --- |
| static void dsfmt\_init\_gen\_rand | ( | dsfmt\_t \* | *dsfmt*, |
|  |  | uint32\_t | *seed* |
|  | ) |  | `[inline]` |

This function initializes the internal state array with a 32-bit integer seed.

**Parameters:**
:   |  |  |
    | --- | --- |
    | dsfmt | dsfmt state vector. |
    | seed | a 32-bit integer used as the seed. |

References dsfmt\_chk\_init\_gen\_rand(), and DSFMT\_MEXP.

Referenced by dsfmt\_gv\_init\_gen\_rand().

|  |  |  |  |
| --- | --- | --- | --- |
| static void fill\_array\_close1\_open2 | ( | double | *array*[], |
|  |  | int | *size* |
|  | ) |  | `[inline]` |

This function is juset the same as dsfmt\_gv\_fill\_array\_close1\_open2().

**Parameters:**
:   |  |  |
    | --- | --- |
    | array | an array where pseudorandom numbers are filled by this function. |
    | size | the number of pseudorandom numbers to be generated. see also |

See also:
:   dsfmt\_fill\_array\_close1\_open2(),
:   dsfmt\_gv\_fill\_array\_close1\_open2()

References dsfmt\_gv\_fill\_array\_close1\_open2().

|  |  |  |  |
| --- | --- | --- | --- |
| static void fill\_array\_close\_open | ( | double | *array*[], |
|  |  | int | *size* |
|  | ) |  | `[inline]` |

This function is juset the same as dsfmt\_gv\_fill\_array\_close\_open().

**Parameters:**
:   |  |  |
    | --- | --- |
    | array | an array where pseudorandom numbers are filled by this function. |
    | size | the number of pseudorandom numbers to be generated. see also |

See also:
:   dsfmt\_gv\_fill\_array\_close\_open(),
:   dsfmt\_fill\_array\_close1\_open2(),
:   dsfmt\_gv\_fill\_array\_close1\_open2()

References dsfmt\_gv\_fill\_array\_close\_open().

|  |  |  |  |
| --- | --- | --- | --- |
| static void fill\_array\_open\_close | ( | double | *array*[], |
|  |  | int | *size* |
|  | ) |  | `[inline]` |

This function is juset the same as dsfmt\_gv\_fill\_array\_open\_close().

**Parameters:**
:   |  |  |
    | --- | --- |
    | array | an array where pseudorandom numbers are filled by this function. |
    | size | the number of pseudorandom numbers to be generated. see also |

See also:
:   dsfmt\_gv\_fill\_array\_open\_close(),
:   dsfmt\_fill\_array\_close1\_open2(),
:   dsfmt\_gv\_fill\_array\_close1\_open2()

References dsfmt\_gv\_fill\_array\_open\_close().

|  |  |  |  |
| --- | --- | --- | --- |
| static void fill\_array\_open\_open | ( | double | *array*[], |
|  |  | int | *size* |
|  | ) |  | `[inline]` |

This function is juset the same as dsfmt\_gv\_fill\_array\_open\_open().

**Parameters:**
:   |  |  |
    | --- | --- |
    | array | an array where pseudorandom numbers are filled by this function. |
    | size | the number of pseudorandom numbers to be generated. see also |

See also:
:   dsfmt\_gv\_fill\_array\_open\_open(),
:   dsfmt\_fill\_array\_close1\_open2(),
:   dsfmt\_gv\_fill\_array\_close1\_open2()

References dsfmt\_gv\_fill\_array\_open\_open().

|  |  |  |  |  |  |
| --- | --- | --- | --- | --- | --- |
| static double genrand\_close1\_open2 | ( | void |  | ) | `[inline]` |

This function is just the same as dsfmt\_gv\_genrand\_close1\_open2().

Returns:
:   double precision floating point number. see also

See also:
:   dsfmt\_genrand\_close1\_open2()
:   dsfmt\_gv\_genrand\_close1\_open2()

References dsfmt\_gv\_genrand\_close1\_open2().

|  |  |  |  |  |  |
| --- | --- | --- | --- | --- | --- |
| static double genrand\_close\_open | ( | void |  | ) | `[inline]` |

This function is just the same as dsfmt\_gv\_genrand\_close\_open().

Returns:
:   double precision floating point number. see also

See also:
:   dsfmt\_genrand\_close\_open()
:   dsfmt\_gv\_genrand\_close\_open()

References dsfmt\_gv\_genrand\_close\_open().

|  |  |  |  |  |  |
| --- | --- | --- | --- | --- | --- |
| static double genrand\_open\_close | ( | void |  | ) | `[inline]` |

This function is just the same as dsfmt\_gv\_genrand\_open\_close().

Returns:
:   double precision floating point number. see also

See also:
:   dsfmt\_genrand\_open\_close()
:   dsfmt\_gv\_genrand\_open\_close()

References dsfmt\_gv\_genrand\_open\_close().

|  |  |  |  |  |  |
| --- | --- | --- | --- | --- | --- |
| static double genrand\_open\_open | ( | void |  | ) | `[inline]` |

This function is just the same as dsfmt\_gv\_genrand\_open\_open().

Returns:
:   double precision floating point number. see also

See also:
:   dsfmt\_genrand\_open\_open()
:   dsfmt\_gv\_genrand\_open\_open()

References dsfmt\_gv\_genrand\_open\_open().

|  |  |  |  |  |  |
| --- | --- | --- | --- | --- | --- |
| static const char \* get\_idstring | ( | void |  | ) | `[inline]` |

This function is just the same as dsfmt\_get\_idstring().

Returns:
:   id string. see also

See also:
:   dsfmt\_get\_idstring()

References dsfmt\_get\_idstring().

|  |  |  |  |  |  |
| --- | --- | --- | --- | --- | --- |
| static int get\_min\_array\_size | ( | void |  | ) | `[inline]` |

This function is just the same as dsfmt\_get\_min\_array\_size().

Returns:
:   minimum size of array used for fill\_array functions. see also

See also:
:   dsfmt\_get\_min\_array\_size()

References dsfmt\_get\_min\_array\_size().

|  |  |  |  |
| --- | --- | --- | --- |
| static void init\_by\_array | ( | uint32\_t | *init\_key*[], |
|  |  | int | *key\_length* |
|  | ) |  | `[inline]` |

This function is just the same as dsfmt\_gv\_init\_by\_array().

**Parameters:**
:   |  |  |
    | --- | --- |
    | init\_key | the array of 32-bit integers, used as a seed. |
    | key\_length | the length of init\_key. see also |

See also:
:   dsfmt\_gv\_init\_by\_array(),
:   dsfmt\_init\_by\_array().

References dsfmt\_gv\_init\_by\_array().

|  |  |  |  |  |  |
| --- | --- | --- | --- | --- | --- |
| static void init\_gen\_rand | ( | uint32\_t | *seed* | ) | `[inline]` |

This function is just the same as dsfmt\_gv\_init\_gen\_rand().

**Parameters:**
:   |  |  |
    | --- | --- |
    | seed | a 32-bit integer used as the seed. see also |

See also:
:   dsfmt\_gv\_init\_gen\_rand(),
:   dsfmt\_init\_gen\_rand().

References dsfmt\_gv\_init\_gen\_rand().

---

## Variable Documentation

|  |
| --- |
| dsfmt\_t dsfmt\_global\_data |

dsfmt internal state vector

|  |
| --- |
| const int dsfmt\_global\_mexp |

dsfmt mexp for check


---

Generated on Fri Jun 29 2012 16:17:32 for dSFMT by  

 1.8.0
