# Supplementary material for: Radiative transfer with reciprocal transactions: Numerical method and its implementation
Source: PLoS One. 2019 Jan 8;14(1):e0210155. doi: 10.1371/journal.pone.0210155 (PMC6324827; doi:10.1371/journal.pone.0210155)
Supplement: S1 Source Code — A link to the latest version: https://bitbucket.org/planetarysystemresearch/r2t2_pub. (ZIP) [file pone.0210155.s001.zip › r2t2_pub/src/dsfmt/dsfmt/html/d_s_f_m_t_8c.html]

dSFMT: dSFMT.c File Reference


|  |
| --- |
| dSFMT  2.2 |

- Main Page
- Data Structures
- Files

- File List
- Globals

Functions |
Variables

dSFMT.c File Reference

double precision SIMD-oriented Fast Mersenne Twister (dSFMT) based on IEEE 754 format.
More...

`#include <stdio.h>`  
`#include <string.h>`  
`#include <stdlib.h>`  
`#include "dSFMT-params.h"`  
`#include "dSFMT-common.h"`

|  |  |
| --- | --- |
| Functions | |
| static uint32\_t | ini\_func1 (uint32\_t x) |
|  | This function represents a function used in the initialization by init\_by\_array. |
| static uint32\_t | ini\_func2 (uint32\_t x) |
|  | This function represents a function used in the initialization by init\_by\_array. |
| static void | gen\_rand\_array\_c1o2 (dsfmt\_t \*dsfmt, w128\_t \*array, int size) |
|  | This function fills the user-specified array with double precision floating point pseudorandom numbers of the IEEE 754 format. |
| static void | gen\_rand\_array\_c0o1 (dsfmt\_t \*dsfmt, w128\_t \*array, int size) |
|  | This function fills the user-specified array with double precision floating point pseudorandom numbers of the IEEE 754 format. |
| static void | gen\_rand\_array\_o0c1 (dsfmt\_t \*dsfmt, w128\_t \*array, int size) |
|  | This function fills the user-specified array with double precision floating point pseudorandom numbers of the IEEE 754 format. |
| static void | gen\_rand\_array\_o0o1 (dsfmt\_t \*dsfmt, w128\_t \*array, int size) |
|  | This function fills the user-specified array with double precision floating point pseudorandom numbers of the IEEE 754 format. |
| static int | idxof (int i) |
|  | This function simulate a 32-bit array index overlapped to 64-bit array of LITTLE ENDIAN in BIG ENDIAN machine. |
| static void | initial\_mask (dsfmt\_t \*dsfmt) |
|  | This function initializes the internal state array to fit the IEEE 754 format. |
| static void | period\_certification (dsfmt\_t \*dsfmt) |
|  | This function certificate the period of 2^{SFMT\_MEXP}-1. |
| static void | convert\_c0o1 (w128\_t \*w) |
|  | This function converts the double precision floating point numbers which distribute uniformly in the range [1, 2) to those which distribute uniformly in the range [0, 1). |
| static void | convert\_o0c1 (w128\_t \*w) |
|  | This function converts the double precision floating point numbers which distribute uniformly in the range [1, 2) to those which distribute uniformly in the range (0, 1]. |
| static void | convert\_o0o1 (w128\_t \*w) |
|  | This function converts the double precision floating point numbers which distribute uniformly in the range [1, 2) to those which distribute uniformly in the range (0, 1). |
| const char \* | dsfmt\_get\_idstring (void) |
|  | This function returns the identification string. |
| int | dsfmt\_get\_min\_array\_size (void) |
|  | This function returns the minimum size of array used for **fill\_array** functions. |
| void | dsfmt\_gen\_rand\_all (dsfmt\_t \*dsfmt) |
|  | This function fills the internal state array with double precision floating point pseudorandom numbers of the IEEE 754 format. |
| void | dsfmt\_fill\_array\_close1\_open2 (dsfmt\_t \*dsfmt, double array[], int size) |
|  | This function generates double precision floating point pseudorandom numbers which distribute in the range [1, 2) to the specified array[] by one call. |
| void | dsfmt\_fill\_array\_open\_close (dsfmt\_t \*dsfmt, double array[], int size) |
|  | This function generates double precision floating point pseudorandom numbers which distribute in the range (0, 1] to the specified array[] by one call. |
| void | dsfmt\_fill\_array\_close\_open (dsfmt\_t \*dsfmt, double array[], int size) |
|  | This function generates double precision floating point pseudorandom numbers which distribute in the range [0, 1) to the specified array[] by one call. |
| void | dsfmt\_fill\_array\_open\_open (dsfmt\_t \*dsfmt, double array[], int size) |
|  | This function generates double precision floating point pseudorandom numbers which distribute in the range (0, 1) to the specified array[] by one call. |
| void | dsfmt\_chk\_init\_gen\_rand (dsfmt\_t \*dsfmt, uint32\_t seed, int mexp) |
|  | This function initializes the internal state array with a 32-bit integer seed. |
| void | dsfmt\_chk\_init\_by\_array (dsfmt\_t \*dsfmt, uint32\_t init\_key[], int key\_length, int mexp) |
|  | This function initializes the internal state array, with an array of 32-bit integers used as the seeds. |
| Variables | |
| dsfmt\_t | dsfmt\_global\_data |
|  | dsfmt internal state vector |
| static const int | dsfmt\_mexp = DSFMT\_MEXP |
|  | dsfmt mexp for check |

---

## Detailed Description

double precision SIMD-oriented Fast Mersenne Twister (dSFMT) based on IEEE 754 format.

Author:
:   Mutsuo Saito (Hiroshima University)
:   Makoto Matsumoto (Hiroshima University)

Copyright (C) 2007,2008 Mutsuo Saito, Makoto Matsumoto and Hiroshima University. All rights reserved.

The new BSD License is applied to this software, see LICENSE.txt

---

## Function Documentation

|  |  |  |  |  |  |
| --- | --- | --- | --- | --- | --- |
| static void convert\_c0o1 | ( | w128\_t \* | *w* | ) | `[inline, static]` |

This function converts the double precision floating point numbers which distribute uniformly in the range [1, 2) to those which distribute uniformly in the range [0, 1).

**Parameters:**
:   |  |  |
    | --- | --- |
    | w | 128bit stracture of double precision floating point numbers (I/O) |

References W128\_T::d.

Referenced by gen\_rand\_array\_c0o1().

|  |  |  |  |  |  |
| --- | --- | --- | --- | --- | --- |
| static void convert\_o0c1 | ( | w128\_t \* | *w* | ) | `[inline, static]` |

This function converts the double precision floating point numbers which distribute uniformly in the range [1, 2) to those which distribute uniformly in the range (0, 1].

**Parameters:**
:   |  |  |
    | --- | --- |
    | w | 128bit stracture of double precision floating point numbers (I/O) |

References W128\_T::d.

Referenced by gen\_rand\_array\_o0c1().

|  |  |  |  |  |  |
| --- | --- | --- | --- | --- | --- |
| static void convert\_o0o1 | ( | w128\_t \* | *w* | ) | `[inline, static]` |

This function converts the double precision floating point numbers which distribute uniformly in the range [1, 2) to those which distribute uniformly in the range (0, 1).

**Parameters:**
:   |  |  |
    | --- | --- |
    | w | 128bit stracture of double precision floating point numbers (I/O) |

References W128\_T::d, and W128\_T::u.

Referenced by gen\_rand\_array\_o0o1().

|  |  |  |  |
| --- | --- | --- | --- |
| void dsfmt\_chk\_init\_by\_array | ( | dsfmt\_t \* | *dsfmt*, |
|  |  | uint32\_t | *init\_key*[], |
|  |  | int | *key\_length*, |
|  |  | int | *mexp* |
|  | ) |  |  |

This function initializes the internal state array, with an array of 32-bit integers used as the seeds.

**Parameters:**
:   |  |  |
    | --- | --- |
    | dsfmt | dsfmt state vector. |
    | init\_key | the array of 32-bit integers, used as a seed. |
    | key\_length | the length of init\_key. |
    | mexp | caller's mersenne expornent |

References DSFMT\_N, DSFMT\_N64, DSFMT\_T::idx, idxof(), ini\_func1(), ini\_func2(), initial\_mask(), period\_certification(), DSFMT\_T::status, and W128\_T::u32.

Referenced by dsfmt\_init\_by\_array().

|  |  |  |  |
| --- | --- | --- | --- |
| void dsfmt\_chk\_init\_gen\_rand | ( | dsfmt\_t \* | *dsfmt*, |
|  |  | uint32\_t | *seed*, |
|  |  | int | *mexp* |
|  | ) |  |  |

This function initializes the internal state array with a 32-bit integer seed.

**Parameters:**
:   |  |  |
    | --- | --- |
    | dsfmt | dsfmt state vector. |
    | seed | a 32-bit integer used as the seed. |
    | mexp | caller's mersenne expornent |

References DSFMT\_N, DSFMT\_N64, DSFMT\_T::idx, idxof(), initial\_mask(), period\_certification(), DSFMT\_T::status, and W128\_T::u32.

Referenced by dsfmt\_init\_gen\_rand().

|  |  |  |  |
| --- | --- | --- | --- |
| void dsfmt\_fill\_array\_close1\_open2 | ( | dsfmt\_t \* | *dsfmt*, |
|  |  | double | *array*[], |
|  |  | int | *size* |
|  | ) |  |  |

This function generates double precision floating point pseudorandom numbers which distribute in the range [1, 2) to the specified array[] by one call.

The number of pseudorandom numbers is specified by the argument **size**, which must be at least (SFMT\_MEXP / 128) \* 2 and a multiple of two. The function get\_min\_array\_size() returns this minimum size. The generation by this function is much faster than the following fill\_array\_xxx functions.

For initialization, init\_gen\_rand() or init\_by\_array() must be called before the first call of this function. This function can not be used after calling genrand\_xxx functions, without initialization.

**Parameters:**
:   |  |  |
    | --- | --- |
    | dsfmt | dsfmt state vector. |
    | array | an array where pseudorandom numbers are filled by this function. The pointer to the array must be "aligned" (namely, must be a multiple of 16) in the SIMD version, since it refers to the address of a 128-bit integer. In the standard C version, the pointer is arbitrary. |
    | size | the number of 64-bit pseudorandom integers to be generated. size must be a multiple of 2, and greater than or equal to (SFMT\_MEXP / 128) \* 2. |

Note:
:   **memalign** or **posix\_memalign** is available to get aligned memory. Mac OSX doesn't have these functions, but **malloc** of OSX returns the pointer to the aligned memory block.

References DSFMT\_N64, and gen\_rand\_array\_c1o2().

Referenced by dsfmt\_gv\_fill\_array\_close1\_open2().

|  |  |  |  |
| --- | --- | --- | --- |
| void dsfmt\_fill\_array\_close\_open | ( | dsfmt\_t \* | *dsfmt*, |
|  |  | double | *array*[], |
|  |  | int | *size* |
|  | ) |  |  |

This function generates double precision floating point pseudorandom numbers which distribute in the range [0, 1) to the specified array[] by one call.

This function is the same as fill\_array\_close1\_open2() except the distribution range.

**Parameters:**
:   |  |  |
    | --- | --- |
    | array | an array where pseudorandom numbers are filled by this function. |
    | dsfmt | dsfmt state vector. |
    | size | the number of pseudorandom numbers to be generated. see also |

See also:
:   fill\_array\_close1\_open2()

References DSFMT\_N64, and gen\_rand\_array\_c0o1().

Referenced by dsfmt\_gv\_fill\_array\_close\_open().

|  |  |  |  |
| --- | --- | --- | --- |
| void dsfmt\_fill\_array\_open\_close | ( | dsfmt\_t \* | *dsfmt*, |
|  |  | double | *array*[], |
|  |  | int | *size* |
|  | ) |  |  |

This function generates double precision floating point pseudorandom numbers which distribute in the range (0, 1] to the specified array[] by one call.

This function is the same as fill\_array\_close1\_open2() except the distribution range.

**Parameters:**
:   |  |  |
    | --- | --- |
    | dsfmt | dsfmt state vector. |
    | array | an array where pseudorandom numbers are filled by this function. |
    | size | the number of pseudorandom numbers to be generated. see also |

See also:
:   fill\_array\_close1\_open2()

References DSFMT\_N64, and gen\_rand\_array\_o0c1().

Referenced by dsfmt\_gv\_fill\_array\_open\_close().

|  |  |  |  |
| --- | --- | --- | --- |
| void dsfmt\_fill\_array\_open\_open | ( | dsfmt\_t \* | *dsfmt*, |
|  |  | double | *array*[], |
|  |  | int | *size* |
|  | ) |  |  |

This function generates double precision floating point pseudorandom numbers which distribute in the range (0, 1) to the specified array[] by one call.

This function is the same as fill\_array\_close1\_open2() except the distribution range.

**Parameters:**
:   |  |  |
    | --- | --- |
    | dsfmt | dsfmt state vector. |
    | array | an array where pseudorandom numbers are filled by this function. |
    | size | the number of pseudorandom numbers to be generated. see also |

See also:
:   fill\_array\_close1\_open2()

References DSFMT\_N64, and gen\_rand\_array\_o0o1().

Referenced by dsfmt\_gv\_fill\_array\_open\_open().

|  |  |  |  |  |  |
| --- | --- | --- | --- | --- | --- |
| void dsfmt\_gen\_rand\_all | ( | dsfmt\_t \* | *dsfmt* | ) |  |

This function fills the internal state array with double precision floating point pseudorandom numbers of the IEEE 754 format.

**Parameters:**
:   |  |  |
    | --- | --- |
    | dsfmt | dsfmt state vector. |

References do\_recursion(), DSFMT\_N, and DSFMT\_T::status.

Referenced by dsfmt\_genrand\_close1\_open2(), dsfmt\_genrand\_open\_open(), and dsfmt\_genrand\_uint32().

|  |  |  |  |  |  |
| --- | --- | --- | --- | --- | --- |
| const char\* dsfmt\_get\_idstring | ( | void |  | ) |  |

This function returns the identification string.

The string shows the Mersenne exponent, and all parameters of this generator.

Returns:
:   id string.

Referenced by get\_idstring().

|  |  |  |  |  |  |
| --- | --- | --- | --- | --- | --- |
| int dsfmt\_get\_min\_array\_size | ( | void |  | ) |  |

This function returns the minimum size of array used for **fill\_array** functions.

Returns:
:   minimum size of array used for fill\_array functions.

References DSFMT\_N64.

Referenced by get\_min\_array\_size().

|  |  |  |  |
| --- | --- | --- | --- |
| static void gen\_rand\_array\_c0o1 | ( | dsfmt\_t \* | *dsfmt*, |
|  |  | w128\_t \* | *array*, |
|  |  | int | *size* |
|  | ) |  | `[inline, static]` |

This function fills the user-specified array with double precision floating point pseudorandom numbers of the IEEE 754 format.

**Parameters:**
:   |  |  |
    | --- | --- |
    | dsfmt | dsfmt state vector. |
    | array | an 128-bit array to be filled by pseudorandom numbers. |
    | size | number of 128-bit pseudorandom numbers to be generated. |

References convert\_c0o1(), do\_recursion(), DSFMT\_N, and DSFMT\_T::status.

Referenced by dsfmt\_fill\_array\_close\_open().

|  |  |  |  |
| --- | --- | --- | --- |
| static void gen\_rand\_array\_c1o2 | ( | dsfmt\_t \* | *dsfmt*, |
|  |  | w128\_t \* | *array*, |
|  |  | int | *size* |
|  | ) |  | `[inline, static]` |

This function fills the user-specified array with double precision floating point pseudorandom numbers of the IEEE 754 format.

**Parameters:**
:   |  |  |
    | --- | --- |
    | dsfmt | dsfmt state vector. |
    | array | an 128-bit array to be filled by pseudorandom numbers. |
    | size | number of 128-bit pseudorandom numbers to be generated. |

References do\_recursion(), DSFMT\_N, and DSFMT\_T::status.

Referenced by dsfmt\_fill\_array\_close1\_open2().

|  |  |  |  |
| --- | --- | --- | --- |
| static void gen\_rand\_array\_o0c1 | ( | dsfmt\_t \* | *dsfmt*, |
|  |  | w128\_t \* | *array*, |
|  |  | int | *size* |
|  | ) |  | `[inline, static]` |

This function fills the user-specified array with double precision floating point pseudorandom numbers of the IEEE 754 format.

**Parameters:**
:   |  |  |
    | --- | --- |
    | dsfmt | dsfmt state vector. |
    | array | an 128-bit array to be filled by pseudorandom numbers. |
    | size | number of 128-bit pseudorandom numbers to be generated. |

References convert\_o0c1(), do\_recursion(), DSFMT\_N, and DSFMT\_T::status.

Referenced by dsfmt\_fill\_array\_open\_close().

|  |  |  |  |
| --- | --- | --- | --- |
| static void gen\_rand\_array\_o0o1 | ( | dsfmt\_t \* | *dsfmt*, |
|  |  | w128\_t \* | *array*, |
|  |  | int | *size* |
|  | ) |  | `[inline, static]` |

This function fills the user-specified array with double precision floating point pseudorandom numbers of the IEEE 754 format.

**Parameters:**
:   |  |  |
    | --- | --- |
    | dsfmt | dsfmt state vector. |
    | array | an 128-bit array to be filled by pseudorandom numbers. |
    | size | number of 128-bit pseudorandom numbers to be generated. |

References convert\_o0o1(), do\_recursion(), DSFMT\_N, and DSFMT\_T::status.

Referenced by dsfmt\_fill\_array\_open\_open().

|  |  |  |  |  |  |
| --- | --- | --- | --- | --- | --- |
| static int idxof | ( | int | *i* | ) | `[inline, static]` |

This function simulate a 32-bit array index overlapped to 64-bit array of LITTLE ENDIAN in BIG ENDIAN machine.

Referenced by dsfmt\_chk\_init\_by\_array(), and dsfmt\_chk\_init\_gen\_rand().

|  |  |  |  |  |  |
| --- | --- | --- | --- | --- | --- |
| static uint32\_t ini\_func1 | ( | uint32\_t | *x* | ) | `[inline, static]` |

This function represents a function used in the initialization by init\_by\_array.

**Parameters:**
:   |  |  |
    | --- | --- |
    | x | 32-bit integer |

Returns:
:   32-bit integer

Referenced by dsfmt\_chk\_init\_by\_array().

|  |  |  |  |  |  |
| --- | --- | --- | --- | --- | --- |
| static uint32\_t ini\_func2 | ( | uint32\_t | *x* | ) | `[inline, static]` |

This function represents a function used in the initialization by init\_by\_array.

**Parameters:**
:   |  |  |
    | --- | --- |
    | x | 32-bit integer |

Returns:
:   32-bit integer

Referenced by dsfmt\_chk\_init\_by\_array().

|  |  |  |  |  |  |
| --- | --- | --- | --- | --- | --- |
| static void initial\_mask | ( | dsfmt\_t \* | *dsfmt* | ) | `[static]` |

This function initializes the internal state array to fit the IEEE 754 format.

**Parameters:**
:   |  |  |
    | --- | --- |
    | dsfmt | dsfmt state vector. |

References DSFMT\_N, DSFMT\_T::status, and W128\_T::u.

Referenced by dsfmt\_chk\_init\_by\_array(), and dsfmt\_chk\_init\_gen\_rand().

|  |  |  |  |  |  |
| --- | --- | --- | --- | --- | --- |
| static void period\_certification | ( | dsfmt\_t \* | *dsfmt* | ) | `[static]` |

This function certificate the period of 2^{SFMT\_MEXP}-1.

**Parameters:**
:   |  |  |
    | --- | --- |
    | dsfmt | dsfmt state vector. |

References DSFMT\_N, DSFMT\_T::status, and W128\_T::u.

Referenced by dsfmt\_chk\_init\_by\_array(), and dsfmt\_chk\_init\_gen\_rand().

---

## Variable Documentation

|  |
| --- |
| dsfmt\_t dsfmt\_global\_data |

dsfmt internal state vector

|  |
| --- |
| const int dsfmt\_mexp = DSFMT\_MEXP `[static]` |

dsfmt mexp for check


---

Generated on Fri Jun 29 2012 16:17:32 for dSFMT by  

 1.8.0
