# Supplementary material for: Radiative transfer with reciprocal transactions: Numerical method and its implementation
Source: PLoS One. 2019 Jan 8;14(1):e0210155. doi: 10.1371/journal.pone.0210155 (PMC6324827; doi:10.1371/journal.pone.0210155)
Supplement: S1 Source Code — A link to the latest version: https://bitbucket.org/planetarysystemresearch/r2t2_pub. (ZIP) [file pone.0210155.s001.zip › r2t2_pub/src/dsfmt/dsfmt/html/d_s_f_m_t-common_8h_source.html]

dSFMT: dSFMT-common.h Source File


|  |
| --- |
| dSFMT  2.2 |

- Main Page
- Data Structures
- Files

- File List
- Globals

dSFMT-common.h

Go to the documentation of this file.

```
00001 #pragma once
00002 
00021 #ifndef DSFMT_COMMON_H
00022 #define DSFMT_COMMON_H
00023 
00024 #include "dSFMT.h"
00025 
00026 #if defined(HAVE_SSE2)
00027 #  include <emmintrin.h>
00028 union X128I_T {
00029     uint64_t u[2];
00030     __m128i  i128;
00031 };
00032 union X128D_T {
00033     double d[2];
00034     __m128d d128;
00035 };
00037 static const union X128I_T sse2_param_mask = {{DSFMT_MSK1, DSFMT_MSK2}};
00038 #endif
00039 
00040 #if defined(HAVE_ALTIVEC)
00041 inline static void do_recursion(w128_t *r, w128_t *a, w128_t * b,
00042                                 w128_t *lung) {
00043     const vector unsigned char sl1 = ALTI_SL1;
00044     const vector unsigned char sl1_perm = ALTI_SL1_PERM;
00045     const vector unsigned int sl1_msk = ALTI_SL1_MSK;
00046     const vector unsigned char sr1 = ALTI_SR;
00047     const vector unsigned char sr1_perm = ALTI_SR_PERM;
00048     const vector unsigned int sr1_msk = ALTI_SR_MSK;
00049     const vector unsigned char perm = ALTI_PERM;
00050     const vector unsigned int msk1 = ALTI_MSK;
00051     vector unsigned int w, x, y, z;
00052 
00053     z = a->s;
00054     w = lung->s;
00055     x = vec_perm(w, (vector unsigned int)perm, perm);
00056     y = vec_perm(z, sl1_perm, sl1_perm);
00057     y = vec_sll(y, sl1);
00058     y = vec_and(y, sl1_msk);
00059     w = vec_xor(x, b->s);
00060     w = vec_xor(w, y);
00061     x = vec_perm(w, (vector unsigned int)sr1_perm, sr1_perm);
00062     x = vec_srl(x, sr1);
00063     x = vec_and(x, sr1_msk);
00064     y = vec_and(w, msk1);
00065     z = vec_xor(z, y);
00066     r->s = vec_xor(z, x);
00067     lung->s = w;
00068 }
00069 #elif defined(HAVE_SSE2)
00070 
00077 inline static void do_recursion(w128_t *r, w128_t *a, w128_t *b, w128_t *u) {
00078     __m128i v, w, x, y, z;
00079 
00080     x = a->si;
00081     z = _mm_slli_epi64(x, DSFMT_SL1);
00082     y = _mm_shuffle_epi32(u->si, SSE2_SHUFF);
00083     z = _mm_xor_si128(z, b->si);
00084     y = _mm_xor_si128(y, z);
00085 
00086     v = _mm_srli_epi64(y, DSFMT_SR);
00087     w = _mm_and_si128(y, sse2_param_mask.i128);
00088     v = _mm_xor_si128(v, x);
00089     v = _mm_xor_si128(v, w);
00090     r->si = v;
00091     u->si = y;
00092 }
00093 #else
00094 
00101 inline static void do_recursion(w128_t *r, w128_t *a, w128_t * b,
00102                                 w128_t *lung) {
00103     uint64_t t0, t1, L0, L1;
00104 
00105     t0 = a->u[0];
00106     t1 = a->u[1];
00107     L0 = lung->u[0];
00108     L1 = lung->u[1];
00109     lung->u[0] = (t0 << DSFMT_SL1) ^ (L1 >> 32) ^ (L1 << 32) ^ b->u[0];
00110     lung->u[1] = (t1 << DSFMT_SL1) ^ (L0 >> 32) ^ (L0 << 32) ^ b->u[1];
00111     r->u[0] = (lung->u[0] >> DSFMT_SR) ^ (lung->u[0] & DSFMT_MSK1) ^ t0;
00112     r->u[1] = (lung->u[1] >> DSFMT_SR) ^ (lung->u[1] & DSFMT_MSK2) ^ t1;
00113 }
00114 #endif
00115 #endif
```


---

Generated on Fri Jun 29 2012 16:17:32 for dSFMT by  

 1.8.0
