# Supplementary material for: Radiative transfer with reciprocal transactions: Numerical method and its implementation
Source: PLoS One. 2019 Jan 8;14(1):e0210155. doi: 10.1371/journal.pone.0210155 (PMC6324827; doi:10.1371/journal.pone.0210155)
Supplement: S1 Source Code — A link to the latest version: https://bitbucket.org/planetarysystemresearch/r2t2_pub. (ZIP) [file pone.0210155.s001.zip › r2t2_pub/src/dsfmt/dsfmt/html/d_s_f_m_t-common_8h.html]

dSFMT: dSFMT-common.h File Reference


|  |
| --- |
| dSFMT  2.2 |

- Main Page
- Data Structures
- Files

- File List
- Globals

Functions

dSFMT-common.h File Reference

SIMD oriented Fast Mersenne Twister(SFMT) pseudorandom number generator with jump function.
More...

`#include "dSFMT.h"`

Go to the source code of this file.

|  |  |
| --- | --- |
| Functions | |
| static void | do\_recursion (w128\_t \*r, w128\_t \*a, w128\_t \*b, w128\_t \*lung) |
|  | This function represents the recursion formula. |

---

## Detailed Description

SIMD oriented Fast Mersenne Twister(SFMT) pseudorandom number generator with jump function.

This file includes common functions used in random number generation and jump.

Author:
:   Mutsuo Saito (Hiroshima University)
:   Makoto Matsumoto (The University of Tokyo)

Copyright (C) 2006, 2007 Mutsuo Saito, Makoto Matsumoto and Hiroshima University. Copyright (C) 2012 Mutsuo Saito, Makoto Matsumoto, Hiroshima University and The University of Tokyo. All rights reserved.

The 3-clause BSD License is applied to this software, see LICENSE.txt

---

## Function Documentation

|  |  |  |  |
| --- | --- | --- | --- |
| static void do\_recursion | ( | w128\_t \* | *r*, |
|  |  | w128\_t \* | *a*, |
|  |  | w128\_t \* | *b*, |
|  |  | w128\_t \* | *lung* |
|  | ) |  | `[inline, static]` |

This function represents the recursion formula.

**Parameters:**
:   |  |  |
    | --- | --- |
    | r | output 128-bit |
    | a | a 128-bit part of the internal state array |
    | b | a 128-bit part of the internal state array |
    | lung | a 128-bit part of the internal state array (I/O) |

References W128\_T::u.

Referenced by dsfmt\_gen\_rand\_all(), gen\_rand\_array\_c0o1(), gen\_rand\_array\_c1o2(), gen\_rand\_array\_o0c1(), and gen\_rand\_array\_o0o1().


---

Generated on Fri Jun 29 2012 16:17:32 for dSFMT by  

 1.8.0
