# Supplementary material for: Radiative transfer with reciprocal transactions: Numerical method and its implementation
Source: PLoS One. 2019 Jan 8;14(1):e0210155. doi: 10.1371/journal.pone.0210155 (PMC6324827; doi:10.1371/journal.pone.0210155)
Supplement: S1 Source Code — A link to the latest version: https://bitbucket.org/planetarysystemresearch/r2t2_pub. (ZIP) [file pone.0210155.s001.zip › r2t2_pub/src/dsfmt/dsfmt/html/classes.html]

dSFMT: Data Structure Index


|  |
| --- |
| dSFMT  2.2 |

- Main Page
- Data Structures
- Files

- Data Structures
- Data Fields

Data Structure Index

D | W

|  |  |  |  |  |
| --- | --- | --- | --- | --- |
| |  | | --- | | D | | |  | | --- | | W | |  |
|  |
| DSFMT\_T | W128\_T |  |
|  |  |  |

D | W


---

Generated on Fri Jun 29 2012 16:17:32 for dSFMT by  

 1.8.0
